# Effectiveness of interventions to address different types of vulnerabilities in community‐dwelling older adults: An umbrella review

**DOI:** 10.1002/cl2.1323

**Published:** 2023-05-09

**Authors:** Rhéda Adekpedjou, Pascale Léon, Omar Dewidar, Ali Al‐Zubaidi, Jalila Jbilou, Janusz Kaczorowski, John Muscedere, John Hirdes, George Heckman, Magali Girard, Paul C. Hébert

**Affiliations:** ^1^ Carrefour de l'innovation et de l'évaluation en santé Centre Hospitalier de l'Université de Montréal Montréal Canada; ^2^ Bruyère Research Institute University of Ottawa Ottawa Ontario Canada; ^3^ Centre de formation médicale du Nouveau‐Brunswick et École de psychologie, Faculté des sciences de la santé et des services communautaires Université de Moncton Nouveau‐Brunswick Canada; ^4^ Department of Critical Care Medicine Queens University and Canadian Frailty Network Kingston Ontario Canada; ^5^ School of Public Health and Health Systems University of Waterloo Waterloo Canada; ^6^ Schlegel Research Institute for Aging Waterloo Canada; ^7^ Bureau de Recherche Développement Valorisation Université de Montréal Montréal Canada

## Abstract

**Background:**

Frailty, social isolation, loneliness, and poverty may render older adults vulnerable to social or health stressors. It is imperative to identify effective interventions to address them especially in the context of COVID‐19 pandemic.

**Objective:**

To identify effective community‐based interventions to address frailty, social isolation, loneliness, and poverty among community‐dwelling older adults.

**Design:**

Umbrella review.

**Data Source:**

We systematically searched PubMed, Ovid MEDLINE, Embase, Cochrane CENTRAL, EBM‐Reviews, CINAHL via EBSCO, and APA PsycInfo via Ovid from January 2009 to December 2022.

**Eligibility Criteria:**

We included systematic reviews or quantitative reviews of non‐pharmacologic interventions targeting community‐dwelling older adults.

**Data Selection, Extraction, and Management:**

Two review authors independently screened the titles and abstracts, performed data extraction and appraised the methodological quality of the reviews. We used a narrative synthesis approach to summarize and interpret the findings. We assessed the methodological quality of the studies using AMSTAR 2.0 tool.

**Results:**

We identified 27 reviews incorporating 372 unique primary studies that met our inclusion criteria. Ten of the reviews included studies conducted in low‐middle‐income countries. Twelve reviews (46%, 12/26) included interventions that addressed frailty. Seventeen reviews (65%, 17/26) included interventions that addressed either social isolation or loneliness. Eighteen reviews included studies with single component interventions, while 23 reviews included studies with multi‐component interventions. Interventions including protein supplementation combined with physical activity may improve outcomes including frailty status, grip strength, and body weight. Physical activity alone or in combination with diet may prevent frailty. Additionally, physical activity may improve social functioning and interventions using digital technologies may decrease social isolation and loneliness. We did not find any review of interventions addressing poverty among older adults. We also noted that few reviews addressed multiple vulnerabilities within the same study, specifically addressed vulnerability among ethnic and sexual minority groups, or examined interventions that engaged communities and adapted programs to local needs.

**Conclusion:**

Evidence from reviews support diets, physical activity, and digital technologies to improve frailty, social isolation or loneliness. However, interventions examined were primarily conducted under optimal conditions. There is a need for further interventions in community settings and conducted under real world settings in older adults living with multiple vulnerabilities.

## 1 PLAIN LANGUAGE SUMMARY

### 1.1 Diets, exercise and digital approaches may improve frailty, social isolation or loneliness in older adults

Exercise alone and in combination with diet may prevent frailty and improve social functioning in community‐dwelling older adults. Similarly, digital tools like connecting with others through the Internet may be effective in reducing loneliness. However, there is no clear evidence that such programmes are effective in groups including the LGBTQ2 + community and ethnic minorities.

### 1.2 What is this review of reviews about?

Social isolation, loneliness and frailty are a serious public health risks that that affect many older adults, specifically people living in their own homes. Several interventions have been proposed to reduce these vulnerabilities. However, the effectiveness of these interventions is inconsistent in the general population, and unknown in specific populations.

**What is the aim of this review?**

This review of reviews summarises evidence on the effectiveness of interventions aimed at improving social isolation, loneliness and frailty among older adults. It also identifies gaps in evidence where further systematic review evidence is needed.

### 1.3 What studies are included?

We included 27 reviews that were comprised of 372 unique primary studies. The vast majority of the reviews included studies that were conducted in high‐income countries. Most reviews talked about either social isolation or loneliness.

Half of the reviews included studies with simple interventions, while the other half were more complicated, with many components.

Many of the studies had important weaknesses.

### 1.4 What are the main findings of this review?

Systematic reviews suggest that exercise combined with nutritional supplementation have the highest odds of decreasing frailty, compared to nutritional supplementation of proteins alone at 3‐4 months of follow‐up.

Similarly, grip strength significantly improves when participants exercise and take protein supplements. Physical activity interventions also improve social functioning and reduce social isolation and loneliness.

There is a lot of conflicting evidence and inadequate reporting of results to determine effectiveness.

We were unable to find studies that looked at minority groups.

### 1.5 What do the findings of this review mean?

Even though there is evidence in support of some interventions, only a small number of reviews systematically compared effects of interventions on social isolation and loneliness.

More studies are needed addressing other vulnerable groups or older adults living with vulnerabilities. This would allow for more definitive recommendations regarding the effectiveness of interventions for reducing frailty, social isolation and loneliness.

### 1.6 How up‐to‐date is this review?

The search was conducted up to December 2022.

## 2 BACKGROUND

Population ageing is increasingly prevalent across the globe, starting in high‐income countries, and now in low‐and middle‐income countries (LMICs) (World Health Organization, 2021). By 2050, two‐thirds of the world's population over 60 years of age will live in LMICs (WHO, 2022). Ageing results in biological changes that gradually deteriorate physical and mental capacities that increases the risk of disease and mortality. In turn, older age is characterized by the emergence of several complex health states commonly called geriatric syndromes. They are often the consequence of multiple underlying factors including frailty, urinary incontinence, falls, and pressure ulcers. These conditions decline the quality of life and the ability of older adults to contribute to their families and communities.

Adverse health outcomes in older adults are influenced not just by biomedical factors but also by vulnerabilities that could manifest in multiple ways (Department of Economic and Social Affairs programme on ageing, 2020; Chen & Schulz, 2016; Hoogendijk et al., 2019; Luanaigh & Lawlor, 2008; Waddell et al., 2018). Frailty defined as a state which affects individuals who experiences an accumulation of deficits in physical, psychological, and social domains leading to worsened adverse outcomes such as mortality and disability (Rockwood & Mitnitski, 2007). Similarly, social isolation, which is known as the subjective feeling of isolation, lack of belonging (Ekwall et al., 2005), loneliness, which is the subjective perception of feeling alone and absence of social network (Prohaska et al., 2020; World Health Organization, 2023), and minimal social engagement are prevalent among older adults and are all associated with a broad range of adverse outcomes. Financial vulnerability could further impede the ability of older adults to manage their illnesses and further threaten their wellbeing. Furthermore, infection prevention measures such as physical distancing and lockdowns implemented during the COVID‐19 pandemic have further increased the likelihood of older adults experiencing new vulnerabilities or amplified pre‐existing ones (Sepúlveda‐Loyola et al., 2020).

Social isolation, loneliness, poverty, and frailty are independent concepts but related to each other (Yanguas et al., 2018). Social isolation and loneliness are associated with an increased likelihood of physical frailty among older adults (Gale et al., 2018; Makizako et al., 2018). Evidence suggest a bidirectional relationship between social isolation, loneliness and frailty (Mehrabi & Béland, 2020). Systematic review evidence also suggests a strong relationship between poverty and frailty among older adults (Hayajneh & Rababa, 2021). Individuals whom have experienced a transition to poverty and remained in poverty were at a higher risk of frailty (Watts et al., 2019; Youn et al., 2020). In addition, social isolation among other psychological factors, may explain the relationship between poverty and frailty, supporting the notion of poverty as a complex of interlinked risk factors (Stolz et al., 2017). Furthermore, community‐dwelling older adults are more likely to experience poverty and other types of vulnerabilities, especially with advancing age (Adepoju et al., 2021; Kaplan & Berkman, 2019).

There is strong evidence for the ability of individual physical activity, psychosocial and educational interventions to improve health outcomes in older adults (Sepúlveda‐Loyola et al., 2020). Physical activity programs have been shown to improve reduce falls, improve strength, walking performance, and balance outcomes among frail older adults (Brady et al., 2020; Di Lorito et al., 2021). Social engagement and group activity programs have shown improvements in mental health and emotional well‐being (Fancourt & Steptoe, 2018; Thomson et al., 2018; Noice et al., 2014). However, the effectiveness of the intervention varies according to their characteristics and theory underlying their intervention design (Di Lorito et al., 2021; Dickens et al., 2011). In contrast, the effectiveness of home visiting and befriending schemes is unclear (Cattan et al., 2005). The wide range of interventions and inconsistent findings on their effectiveness indicate a need for a comprehensive summarization and synthesis of the literature. Furthermore, given the interplay between different vulnerabilities, identifying strategies that address at least two of the three vulnerabilities (social isolation, loneliness, frailty) will help develop multi‐component programs that mitigate their effects among older adults.

In conducting a rapid scan of the literature and review of the Campbell and Cochrane databases, we were unable to identify reviews of reviews focused on interventions to mitigate the consequences or alleviate vulnerabilities affecting older adults. Therefore, the objectives of this umbrella review are to (1) identify available systematic review evidence on the effects of interventions in improve social isolation, loneliness, and frailty among community‐dwelling older adults, (2) identify differences in effects of interventions across gender and ethnic minorities, (3) assess gaps in evidence where further systematic review evidence is needed.

## 3 METHODS

This study is part of the Connection New Brunswick project, a project that aims to improve health and social well‐being of vulnerable seniors in four New Brunswick communities through targeted co‐designed programs based on identified vulnerabilities. We created an interdisciplinary committee to lead the review process, meetings (five formal meetings) were conducted to define the key domains of vulnerability, to establish a rigorous method and to validate the analytical approach. We report this review according to the Joanna Briggs Institute's guidance for the conduct and reporting of an umbrella review (Aromataris et al., 2015). We registered the protocol in PROSPERO under the number CRD42020190419.

### 3.1 Eligibility criteria

We based our eligibility criteria on the characteristics of the participants of the primary studies included in the reviews, the nature of the interventions, the type of review and the reported outcomes.

#### 3.1.1 Participants

We included reviews where participants of the primary studies have the following characteristics: (a) at least 50% of the participants were 65 years or over, OR the mean age of the participants was at least 65 years, OR the age cut‐off was not mentioned but the review referred to older adults as the target population, OR the age cut‐off was 50 years and above but the review referred to older adults as the target population; (b) participants were community‐dwelling older adults, (c) participants were healthy or had health conditions including chronic diseases and comorbidities.

We included reviews that provided findings for community‐dwelling adults separately from older adults living in institutional settings. We excluded reviews where the setting of the older adults was not clear.

#### 3.1.2 Intervention(s), exposure(s)

Non‐pharmacologic community‐based interventions that aimed to address frailty, social isolation, loneliness or poverty among older adults. We excluded reviews studying pharmacotherapy only.

#### 3.1.3 Comparator(s)/control

The comparator was any other non‐pharmacological intervention, usual care, no intervention, or placebo intervention.

#### 3.1.4 Types of review

We included any review (e.g., systematic, scoping, rapid) with or without a meta‐analysis that met the following definition (Krnic Martinic et al., 2019): (a) aims to synthesize all the empirical evidence that meets pre‐specified eligibility criteria to answer a specific research question (b) used systematic methods to identify, appraise and synthesize empirical evidence, (c) provided an explicit and reproducible search strategy of at least one or more bibliographic databases (d) synthesized the findings descriptively or quantitatively. Reviews must have included at least one randomized controlled trial (RCT) or non‐randomized study of interventions (NRS) (e.g., quasi‐experimental studies, controlled clinical trials, before and after studies/pre–post design, Interrupted Time Series).

We excluded narrative reviews developed without systematic literature search, review protocols and reviews that were not in English or French given the language capacity of the team members.

#### 3.1.5 Outcomes

We included reviews where frailty status, social isolation or loneliness were identified as primary or secondary outcomes regardless of how they were measured (validated or non‐validated instruments). We also included reviews that reported at least three of the five criteria of Fried's frailty phenotype (weakness, slow gait speed, low physical activity, exhaustion, unintentional weight loss). For social isolation we considered social isolation and its related dimensions such as social functioning, social participation, social activities, social support, social network size, social connectedness.

For this umbrella review, our primary outcomes were measures of improvement in frailty status, Fried's frailty phenotype components (weakness, slow gait speed, low physical activity, exhaustion, and unintentional weight loss), social isolation, loneliness, and poverty. We have selected those outcomes due to evidence of their interrelationship (Yanguas et al., 2018). Client‐centered outcomes examined were improvements or the decrease in risk of adverse health outcomes (e.g., depression, falls, death), health service use (e.g., hospitalization, premature admission to long‐term care) and costs. These outcomes reflect longer‐term real‐world consequences of interventions.

### 3.2 Search strategy

An information scientist from the Centre Hospitalier de l'Université de Montréal library broadly searched in the following electronic databases/sources for published reviews: PubMed, Ovid MEDLINE, Embase, Cochrane CENTRAL, EBM‐Reviews, CINAHL via EBSCO, and APA PsychInfo via Ovid from January 2009 to December 2022. The search was validated by another information scientist experienced in medical research methods (Tamara Rader). We limited the search to studies published from 2009 to 2019 to summarize the most recent available evidence in the last decade according to the most recent definitions of social isolation and loneliness. We later reran the search in December 2022 to update the findings of the review. The final search strategies, which were developed using an iterative process to minimize false positives and optimize results, are described in Supporting Information 1. We searched for relevant systematic reviews in the reference list of the included systematic reviews.

### 3.3 Study selection

Two review authors independently screened the titles and abstracts generated by the search against the inclusion criteria. We retrieved and screened the full‐text reports for all titles that appeared to meet the inclusion criteria in duplicate and independently. We resolved disagreement through discussion or by seeking the opinion of another review author. We documented the reasons for excluding reviews. Neither of the review authors was blind to the journal information, the study authors, or institutions.

### 3.4 Data extraction

Using a standardized data extraction form (in Excel sheets), two reviewers extracted data from the reviews independently. To ensure consistency across reviewers, we conducted calibration exercises before starting the review to develop an extraction guide. Data abstracted included first author's name, year of publication, review's objective (s), type of included studies, the number of included studies, details on participants (sample sizes, age, gender, disease or condition), details on intervention (name, type of deliverer, setting, modes of delivery, duration), details on comparison (name, type of deliverer), outcome measures (frailty, social isolation, loneliness, poverty), type of analysis (qualitative synthesis, meta‐analysis), main results, name of the interventions that have a positive effect, duration of follow‐up, the assessment of risk of bias of the primary studies as reported by the review authors and the evaluation of the certainty of evidence as reported by the review authors. Reviewers resolved disagreements by discussion, or by a third reviewer. When the information provided in the reviews was not clear, we went back to the primary studies included in the selected review to extract these data.

### 3.5 Methodological quality assessment

Two independent reviewers appraised the methodological quality of the retained reviews using the AMSTAR 2 (Assessing the Methodological Quality of Systematic Reviews) critical appraisal tool (Shea et al., 2017). Items covered by this appraisal are presented in Box 1. Disagreements were resolved first by discussion and then by consulting one of the team members for arbitration.

Box 1 **Checklist for critical appraisal of the methodological quality of systematic reviews (AMSTAR 2) (Shea et al., 2017)**

1. Did the research questions and inclusion criteria for the review include the components of PICO?
2. Did the report of the review contain an explicit statement that the review methods were established prior to the conduct of the review and did the report justify any significant deviations from the protocol?
3. Did the review authors explain their selection of the study designs for inclusion in the review?
4. Did the review authors use a comprehensive literature search strategy?
5. Did the review authors perform study selection in duplicate?
6. Did the review authors perform data extraction in duplicate?
7. Did the review authors provide a list of excluded studies and justify the exclusions?
8. Did the review authors describe the included studies in adequate detail?
9. Did the review authors use a satisfactory technique for assessing the risk of bias (RoB) in individual studies that were included in the review?
10. Did the review authors report on the sources of funding for the studies included in the review?
11. If meta‐analysis was performed did the review authors use appropriate methods for statistical combination of results?
12. If meta‐analysis was performed, did the review authors assess the potential impact of RoB in individual studies on the results of the meta‐analysis or other evidence synthesis?
13. Did the review authors account for RoB in individual studies when interpreting/discussing the results of the review?
14. Did the review authors provide a satisfactory explanation for, and discussion of, any heterogeneity observed in the results of the review?
15. If they performed quantitative synthesis did the review authors carry out an adequate investigation of publication bias (small study bias) and discuss its likely impact on the results of the review?
16. Did the review authors report any potential sources of conflict of interest, including any funding they received for conducting the review?

We considered seven methodological quality items as critical. These included item 2 (Protocol registered before commencement of the review), item 4 (Adequacy of the literature search), item 7 (Justification for excluding individual studies), item 9 (Risk of bias from individual studies being included in the review), item 11 (Appropriateness of meta‐analytical methods), item 13 (Consideration of risk of bias when interpreting the results of the review), item 15 (Assessment of presence and likely impact of publication bias). We considered the remaining items as noncritical. We judged not meeting a critical methodological quality item as a *critical flaw*. We judged not meeting a non‐critical methodological quality item as a *non‐critical weakness*. Based on the approach proposed by Shea and colleagues, we judged the overall confidence in the result of a review as *high* (no or one non‐critical weakness), *moderate* (more than one non‐critical weakness), *low* (one critical flaw with or without non‐critical weaknesses) or *critically low* (more than one critical flaw with or without non‐critical weaknesses) (Shea et al., 2017). We did not evaluate the certainty of evidence of the overview using GRADE because it has not been adapted to reviews of reviews. Instead, we collected and reported the certainty of evidence evaluated by the authors of the included reviews.

### 3.6 Strategy for data synthesis

We tabulated data related to characteristics of included reviews. We tabulated the description of the interventions identified in included reviews, the number of participants (from included primary studies), the effect of the interventions and their magnitude as reported in the published reports. We summarized the effects of the interventions descriptively using tables. We also tabulated the assessment of risk of bias of the included primary studies and the evaluation of the quality of the evidence as reported by the review authors. Finally, we mapped the overlapping primary studies contained within the included reviews.

We explored the effect of the interventions within the following subgroups: interventions primarily focusing on either frailty, social isolation, loneliness, adverse health outcomes or costs separately. Reviews that reported multiple outcomes of interest were presented accordingly. We presented separately the results of systematic reviews from other type of reviews (e.g., scoping reviews). We also presented the results of unimodal interventions (e.g., physical activity alone) and multimodal interventions (e.g., interventions combining diet and physical activity) separately. Within each intervention, we distinguished the results of randomized controlled trials from the results of other study designs. We planned to explore the effectiveness of the interventions within the following subgroups: high‐income versus LMICs, urban versus rural setting, men versus women.

## 4 RESULTS

### 4.1 Results of the search

**Figure 1:**
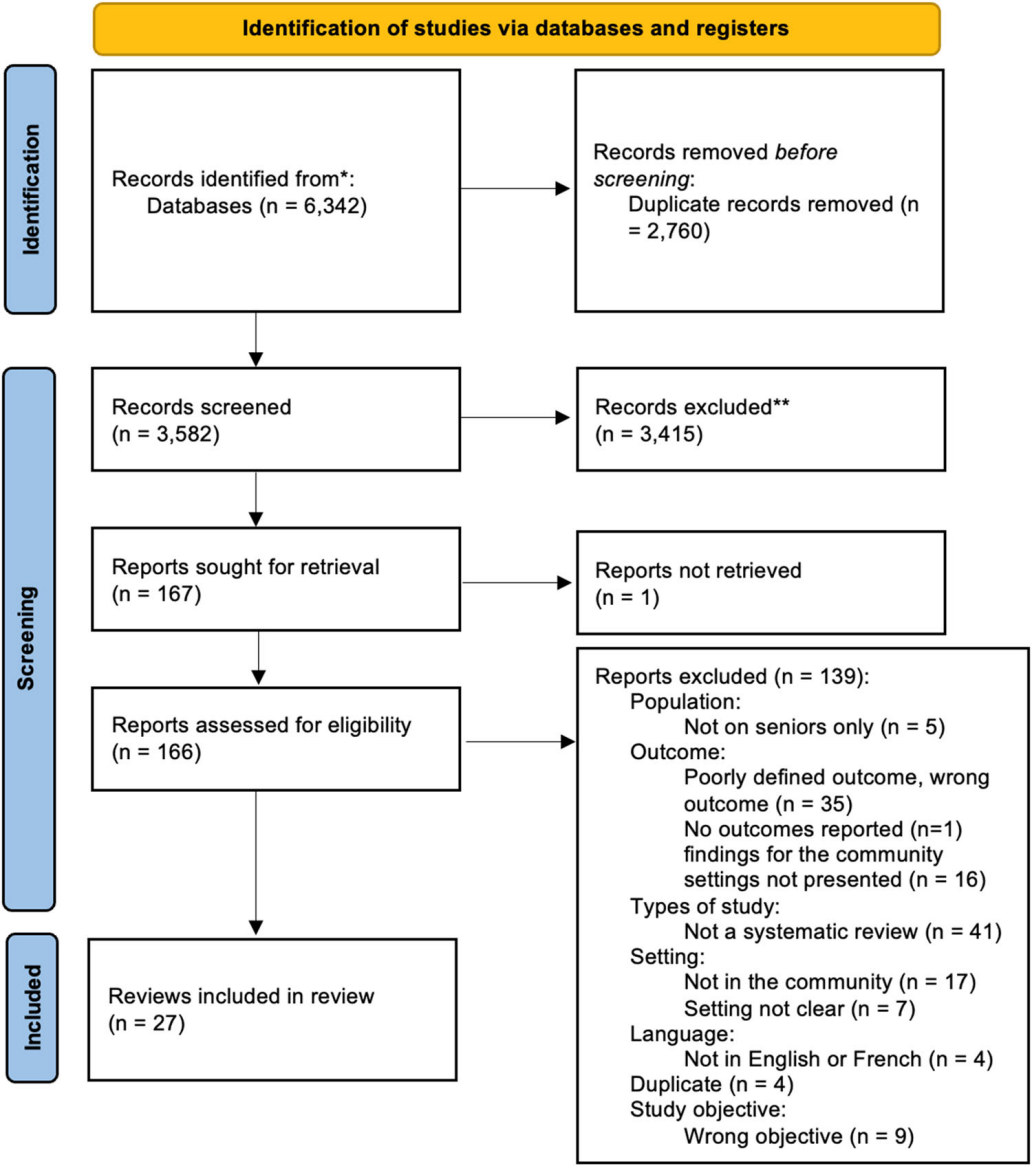
PRISMA flow chart of study selection and inclusion.

The literature search identified 6,342 titles, all from bibliometric databases (Figure 1). After removing duplicates (*n* = 2760), we independently screened the titles and abstracts of 3582 records and we excluded 3415 records as irrelevant to the umbrella review. We reviewed the full reports of the remaining 166 studies for eligibility, of which we included 27 reviews. No additional eligible reviews were identified through reference searching of included reviews.

### 4.2 Characteristics of included reviews

Twenty of the included reviews were systematic reviews and seven were narrative reviews with systematic search or scoping reviews (Anton et al., 2018; Hagan et al., 2014; Ibrahim et al., 2022; Kelaiditi et al., 2014; Pool et al., 2017; Puts et al., 2017; Wister et al., 2021). Seven of the systematic reviews conducted a meta‐analysis (Burton et al., 2019; Frost et al., 2017; Fu et al., 2022; Li et al., 2022; Liao et al., 2018; Shvedko et al., 2018; Walters et al., 2017). Walters and colleagues meta‐analyzed RCTs and NRSs assessing home‐based health interventions separately (Walters et al., 2017). Frost et al. (2017). meta‐analyzed the same set of RCTs. Therefore, to avoid duplicate reporting of findings, we only reported the meta‐analysis findings of the NRS from Walters et al., not their RCT findings (Walters et al., 2017). A Systematic review on social prescribing for older adults conducted by Smith et al. (2019) did not find any eligible studies but was included because it met the eligibility criteria. The 26 reviews included 372 unique primary studies. A total of 64 primary studies were included in multiple reviews (Supporting Information 1). Ten of the reviews included studies conducted in LMICs according to the World bank classification.

### 4.3 Characteristics of the primary studies included in the reviews

The number of eligible RCTs per review ranged from 3 (Pool et al., 2017) to 47 (Theou et al., 2011). The number of eligible non‐randomized studies (NRS) per review ranged from 2 (Puts et al., 2017) to 24 (Khosravi et al., 2016). RCTs included parallel RCTs, pragmatic RCTs, cluster RCTs, randomized crossover trials. NRSs included a variety of designs: single group pre‐ and post‐test studies/before‐and‐after studies, quasi‐experimental pre‐ and post‐test studies with control group, pilot/feasibility/exploratory studies, evaluation/post‐hoc evaluation studies, cohort studies, case‐control studies, cross‐sectional studies, and case studies. Twenty‐five reviews reported on studies sample sizes. The sample size ranged from 3 (Khosravi et al., 2016) to 3689 (Looman et al., 2019) (Table 1). Countries of origin of the primary studies reported in the reviews were in North America (USA, Canada, Mexico), South America (Brazil), Europe (Norway, UK, Germany, The Netherlands, Finland, Spain, Austria, Poland, Denmark, France, Sweden, Hungary, Holland, Italy, Greece), Asia (Taiwan, Japan, China, Korea, Singapore, Malaysia), West Asia (Israel, Turkey), and Oceania (Australia, New Zealand). Of the listed countries, most of the primary studies were conducted in the USA (55%, 16/29), followed by Australia (52%, 15/29), UK (31%, 9/29) and China (28%, 8/29). Three reviews did not report on the geographical settings of the primary studies (Anton et al., 2018; Kelaiditi et al., 2014; Arantes et al., 2009) (Table 1).

**Table 1:** Characteristics of included reviews.

Authors	Review	Primary studies	Interventions	Outcomes
Anton et al. (2017) Anton et al. (2018)	Objective: to review findings from studies that have tested dietary and/or exercise interventions to improve one or more of some key components of sarcopenia and/or frailty: (1) body compositions, (2) walking speed, (3) leg and grip strength Type: Review Method used for synthesis: qualitative synthesis	Design: 19 RCT Sample size (min‐max): 10–196^a^ Medical context: frail, pre‐frail, sarcopenic Mean‐median age (min‐max): 60–84 years % of female (min‐max): unclear Geographical settings: NR	Name: Dietary and/or exercise interventions Mode of delivery: Individual dietary counseling. Group‐based exercise interventions. Individual and class trainer Duration in weeks/months (min–max): 12–52 weeks	Frailty components: weight loss, gait/walking speed, grip strength
Arantes et al. (2009)	Objective: to carry out a systematic review of the literature on physical therapy interventions and their effect on frail community‐dwelling elders Type: systematic review Method used for synthesis: qualitative synthesis	Design: 18 RCT Sample size (min‐max): 11–243 Medical context: frail Mean‐median age (min–max): 73–84 years % of female (min‐max): NR Geographical settings: NR	Name: physical therapy interventions Mode of delivery: Individual and group exercises Duration in weeks/months (min–max): 10 weeks–9 months	Frailty components: weight loss, gait speed, grip strength Adverse health outcomes: depression, pain, falls, institutionalization healthcare cost
Burton et al. (2019)	Objective: to determine the effectiveness of physical activity/exercise programs delivered specifically to older adults receiving home care services Type: systematic review Method used for synthesis: qualitative synthesis and meta‐analysis	Design: 10 RCT, 8 NRS Sample size (min–max): 8–228 Medical context: older adults receiving home care services Mean‐median age (min–max): 74.5–85.4 years % of female (min‐max): 53.8%–83% Geographical settings: Australia, USA, New Zealand, Norway, Hong Kong	Name: physical activity/exercise programs Mode of delivery: Home care, individual (personal care or person‐centered approach) Duration in weeks/months (min–max): 8 weeks–6 months	Frailty status Frailty components: grip strength, gait speed Adverse health outcomes: fall, pain, visits to a general or clinical practice health professional, emergency department/hospital Cost
Franck et al. (2016)	Objective: to ascertain the effectiveness of interventions that address social isolation and depression aged care services clients in rural settings Type: systematic review Method used for synthesis: qualitative synthesis	Design: 2 experimental studies, 1 quasi experimental study pre‐ and post‐test with control group, 2 quasi experimental studies pre‐ and post‐test without control group Sample size (min‐max): 26–113 Medical context: participants receiving aged care services (community or residential) Mean–median age (min–max): 77.42–85.23 years % of female (min‐max): 0%–88.8% Geographical settings: Australia, USA, UK, Hong Kong, Taiwan	Name: Reminiscence therapy, Gender‐based social clubs, Playing Nintendo Wii, Indoor gardening program, Radio program Mode of delivery: Individual, duo activity and group‐based activities Duration in weeks/months (min–max): 8 weeks–3 months	Social isolation Loneliness Adverse health outcomes: depression, anxiety
Frost et al. (2017)	Objective: to synthetize RCTs evaluating home and community‐based health promotion for older people with mild/pre‐frailty Type: systematic review Method used for synthesis: qualitative synthesis and meta‐analysis	Design: 7 RCT Sample size (min–max): 23–194 Medical context: prefrail, frail Mean‐median age (min–max): 72–83 years % of female (min–max): unclear Geographical settings: USA, Germany, Japan, Brazil	Name: home and community‐based health promotion: Balance and strength exercises; Wii fit exercise + seated exercise; Power training + strength training; Strength and balance training + nutrition; Strength and balance training alone; Resistance exercise and Telemonitoring. Mode of delivery: Individual and group exercises Duration in weeks/months (min–max): 10 weeks–12 months	Frailty status Frailty components: handgrip strength, gait speed
Hagan et al. (2014)	Objective: to investigate the effectiveness of recent therapeutic social interventions in reducing loneliness or social isolation in older people and to make recommendations as to the choice of interventions for practice Type: systematic search and narrative review Method used for synthesis: qualitative synthesis	Design: 9 controlled trials, 3 before and after studies, 3 evaluation/post‐hoc evaluation, 2 pilot/exploratory studies Sample size (min–max): 26–1217 Medical context: NR Mean‐median age (min‐max): 63–82 years % of female (min‐max): 54%–100% Geographical settings: UK, USA, Australia, Taiwan, Israel, Holland, Finland	Name: Therapeutic social interventions Mode of delivery: Community‐based group interventions, Supported living group interventions and One‐to‐one interventions. Duration in weeks/months (min–max): NR	Social isolation Loneliness
Kelaiditi et al. (2014)	Objective: to provide a comprehensive review of the research published within the previous 12 months on the role of nutrition and exercise in Frailty Comprehensive review of the literature Method used for synthesis: qualitative synthesis	Design: 5 RCT, 7 NRS Sample size (min–max): 23–4731 Medical context: Disability, cancer and geriatric syndromes (Parkinson's disease). Pre‐frailty and frailty. Mean–median age (min–max): NR % of female (min–max): unclear Geographical settings: NR	Name: nutrition and exercise Mode of delivery: in group and individual. Duration in weeks/months (min–max): 12 weeks–12 months	Frailty status
Khosravi et al. (2016)	Objective: to identify interventions that ascertain the effectiveness of technological interventions in reducing social in older adults Type: systematic literature review Method used for synthesis: qualitative synthesis	Design: 10 RCT, 24 NRS Sample size (min–max): 3–3075 Medical context: Anxiety, depression, handicap and chronic diseases unspecified Mean‐median age (min–max): 54–98 years % of female (min–max): unclear Geographical settings: North America and Canada (60%), Europe (20%), Australia (8%) and other countries (12%)	Name: technological interventions Mode of delivery: group and individual intervention Duration in weeks/months (min–max): 1 week–6 months	Social isolation Loneliness
Liao et al. (2018)	Objective: to analyze the effectiveness of protein supplementation and resistance exercise training or multicomponent exercise training on frailty Type: Systematic review and meta‐analysis Method used for synthesis: meta‐analysis	Design: 22 RCT Sample size (min–max): 24–191 Mean–median age (min–max): 75.7–89.2 years Medical context: frailty % of female (min–max): 36.5%–100% Geographical settings: Canada, Japan, Spain, Austria, Poland, Denmark, France, Sweden and Netherlands and Australia.	Name: protein supplementation and resistance exercise training or multicomponent exercise training Mode of delivery: NR Duration in weeks/months (min–max): 4 weeks–9 months	Frailty status Frailty components: body weight, handgrip strength, gait speed, physical activity, exhaustion
Looman et al. (2019)	Objective: to ascertain the effectiveness and cost‐effectiveness of preventive, integrated care for community‐dwelling frailer older adults, and to ascertain their components and levels of integration Type: systematic review Method used for synthesis: qualitative synthesis	Design: 18 RCT, 3 cluster‐RCT, 1 stepped‐wedge cluster‐RCT, 6 before‐and‐after studies, 1 case‐control study Sample size (min–max): 36–3689 Medical context: frail Mean‐median age (min–max): NR % of female (min–max): NR Geographical settings: Canada, United States, the Netherlands, Sweden, Australia, Finland, France, Hong Kong, Japan and New Zealand	Name: integrated care interventions Mode of delivery: Individual though healthcare services, unclear^a^ Duration in weeks/months (min–max): NR	Frailty status Social isolation: social functioning, social support Adverse health outcomes: pain, fall, mortality, healthcare utilization, costs
Pool et al. (2017)	Objective: to identify interventions that improve social participation and reduce social isolation and loneliness in community‐dwelling older adults Type: review Method used for synthesis: narrative synthesis	Deisgn: 3 RCT, 3 NRS Sample size (min–max): 39–702 Medical context: community‐dwelling older adults of ethnic minority Mean–median age (min–max): 66–73 years % of female (min–max): NR Geographical settings: USA	Name: volunteering activity, educational activity, physical activity, physical activity combined with educational activity Mode of delivery: Group and individual intervention, Group Enhanced with individual feedback Duration in weeks/months (min–max): 12 weeks–24 months	Social isolation: social support, social provision, social activities Loneliness
Shvedko et al. (2018)	Objective: to examine physical activity intervention effects on loneliness, social isolation of low social support in community‐dwelling older adults Type: systematic review and meta‐analysis Method used for synthesis: descriptive summary and meta‐analysis	Design: 38 RCT Sample size (min–max): 17–830 Medical context: community‐dwelling older adults healthy or with a comorbidity but mobile, without dementia or moderate to severe cognitive dysfunction Mean–median age (min–max): 51–82 years % of female (min–max): unclear Geographical settings: USA, UK, Japan, Australia, Taiwan, China, Canada, the Netherlands, New Zealand, Korea, Sweden, Denmark, Finland, Turkey, Spain, Brazil and Hungary	Name: physical activity interventions Mode of delivery: group setting, individual and mixed design settings Duration in weeks/months (min–max): 6 weeks–12 months	Social isolation: social isolation, social support, social functioning, social networks Loneliness
Sims‐Gould et al. (2017)	Objective: to systematically review the impact of reablement, reactivation, rehabilitation, and restorative (4R) programs for older adults in receipt of home care services Type: systematic review Method used for synthesis: narrative, qualitative synthesis	Design: 15 RCT Sample size (min–max): 29–750 Medical context: community dwelling older adults in receipt of home care services Mean–median age (min–max): 76–83 years % of female (min‐max): 51.9%–83% Geographical settings: USA, Norway, New Zealand, Australia, UK and Denmark.	Name: 4R interventions (rehabilitation, restorative, reablement, reactivation) exclusively delivered in the home Mode of delivery: Individual Duration in weeks/months (min–max): 4 weeks–6 months	Frailty status Social isolation: social support Loneliness Adverse health outcomes: health care service usage, falls, pain, and depression
Puts et al. (2017)	Objective: to identify peer‐reviewed and gray literature on policies to prevent/or delay frailty in community‐dwelling older adults Type: scoping review Method used for synthesis: qualitative synthesis	Design: 12 RCT, 2 NRS Sample size (min–max): 51–610 Medical context: prefrail, frail Mean–median age (min–max): 70–86 years % of female (min‐max): 48%–100% Geographical settings: Singapore, Taiwan, Japan, Spain, Sweden, USA, Australia	Name: Multiple interventions Mode of delivery: face‐to‐face (individual), group sessions, home visits Duration in weeks/months (min‐max): 4 weeks–12 months	Frailty status
Dedeyne et al. (2017)	Objective: to provide an overview of the effects of controlled multi‐domain interventions in (pre)frail people aged 65 years or older on frailty status, and score, cognition, muscle mass strength and power, and functional and social outcomes Type: systematic review Method used for synthesis: qualitative synthesis	Design: 12 RCT Sample size (min–max): 31–246 Medical context: prefrail, frail Mean–median age (min–max): 70–83.3 years % of female (min–max): unclear Geographical settings: Europe, USA, Asia	Name: Multi‐domain interventions Mode of delivery: group, individual Duration in weeks/months (min–max): 12 weeks–9 months	Frailty status Social involvement Adverse health outcomes: depression^b^, fall
Coll‐Planas et al. (2017)	Objective: to assess the impact on health outcomes and use of health‐related resources of interventions that promote social capital or its components among older people Type: systematic review Method used for synthesis: narrative synthesis	Design: 36 RCT Sample size (min–max): 30–532 Medical context: older adults with physical chronic diseases, mental health conditions, social conditions and caregivers Mean–median age (min–max): 70–80 years % of female (min‐max): 0%–100% Geographical settings: Southern, Central and Northern Europe, Northern America, Southern America. Asia, Oceania	Name: Social capital interventions Mode of delivery: Individual intervention, group intervention and combined individual with group‐based intervention Duration in weeks/months (min–max): 6 weeks to +12 months	Loneliness Adverse health outcomes: anxiety, depression, mortality
Snowden et al. (2014)	Objective: to identify interventions to promote emotional health of older adults aged 50 years or older Type: systematic literature review Method used for synthesis: qualitative synthesis	Design: 3 RCT, 1 before‐and‐after study Sample size (min–max): NR Medical context: diverse health conditions Mean‐median age (min–max): NR % of female (min‐max): NR Geographical settings: USA and Canada, Europe, Australia, New Zealand, Asia.	Name: social support, strength/resistance Mode of delivery: individual, group interventions Duration in weeks/months (min–max): 6 weeks–12 months	Loneliness Adverse health outcomes: anxiety, stress, distress^b^
Cohen‐Mansfield and Perach (2015)	Objective: to determine the utility of loneliness interventions among older adults, and to ascertain which are effective for specific subgroups Type: systematic review Method used for synthesis: qualitative synthesis	12 RCT, 6 not randomized controlled trials, 4 NRS Sample size (min–max): 9–708 Medical context: older people cognitively intact, receiving care, healthy and ambulatory person and older people with health and social conditions Mean–median age (min–max): NR % of female (min–max): unclear Geographical settings: USA, The Netherlands, Australia, England/UK, Taiwan and Sweden.	Name: Interventions for Alleviating Loneliness Among Older Persons Mode of delivery: group interventions and one‐on‐one intervention Duration in weeks/months (min–max): 2 weeks–36 months	Social isolation: social participation, satisfaction with socialization, daily activities and social contact, social interactions Loneliness
Theou et al. (2011)	Objective: to consider the use of the term frailty in relation to exercise interventions and to examine the effectiveness of exercise interventions on the management of frailty Type: systematic review Method used for synthesis: qualitative synthesis	47 RCT Sample size (min–max): 13–551 Medical context: mild to moderate and severe frailty, inactivity, at risk for major mobility disability, at risk for fall Mean–median age (min–max): 71–83 years % of female (min–max): 0%–100% Geographical settings: USA, Canada, Europe, Japan, New Zealand, Australia.	Name: exercise interventions (multicomponent training, comprehensive training, resistance training, horse riding simulator training) Mode of delivery: supervised group interventions, home‐based programs, simulator training Duration in weeks/months (min–max): 4 weeks–18 months	Frailty status Frailty components: body weight, gait speed, physical activity
Walters et al. (2017)	Objective: to systematically review observational studies of people with mild or pre‐frailty that identify modifiable risk factors for adverse outcomes (e.g., transitioning to frailty, worsening functioning) that could be potential new targets for intervention Type: systematic review Method used for analysis: qualitative synthesis and meta‐analysis^c^	Design: 4 NRS Sample size (min–max): 128–3018 Medical context: prefrailty, mild frailty Mean–median age (min–max): NR % of female (min–max): NR Geographical settings: Italy—Chianti study, Hong Kong, Australia‐ Concord Health and Ageing in Men project.	Name: N/A Mode of delivery: N/A Duration in weeks/months (min–max): N/A	Frailty status
Tricco et al. (2022)	Objective: identify the effective interventions to prevent or mitigate social isolation and/or loneliness in older adults who experienced a fall. Type: Systematic review Method used for analysis: Narrative synthesis	Design: 1 RCT, 3 NRS Sample size (min–max): 21–2325 Medical context: Experienced a fall Mean–median age (min–max): 76%–79.6% of female (min‐max): 60.8–91 Geographical settings: North America, Europe	Name: Any intervention for social isolation Mode of delivery: Combinations of participant homes, community setting and primary care Duration in weeks/months (min–max): 6 months–12 months	Social isolation Loneliness
Li et al. (2022)	Objective: Explore the effectiveness of current interventions in reducing loneliness in Chinese older adults. Type: Systematic review Method used for analysis: Network meta‐analysis	Design: 3 RCT, 15 NRS Sample size (min–max): 44–208 Medical context: None Mean–median age (min–max): NR % of female (min–max): NR Geographical settings: China	Name: Physical activity and psychosocial interventions Mode of delivery: Group, Individual, or Group and Individual Duration in weeks/months (min‐max): 6 weeks–18 months	Loneliness
Smith et al. (2019)	Objective: determine the current evidence on the effectiveness of social prescribing programmes to delay or reduce frailty in frail older adults living in the community. Type: Systematic review Method used for analysis: Narrative synthesis	No primary studies were found	No primary studies were found	No primary studies were found
Ibrahim et al. (2022)	Objective: evaluate health status associated with social interventions to enhance participation among community‐dwelling older people Type: Systematic review Method used for analysis: Qualitative synthesis	Design: 9 RCT, 16 NRS Sample size (min–max): 23–916 Medical context: None Mean‐median age (min–max): NR % of female (min–max): NR Geographical settings: UK, Finland, China, USA, Netherlands, Spain, Japan, Canada, Sweden	Name: Social interventions Mode of delivery: Group, Individual, or Group and Individual Duration in weeks/months (min‐max): 2 weeks–120 months	Social isolation Loneliness
Heins et al. (2021)	Objective: evaluate the effects of technological interventions that target social participation in community‐dwelling older adults with and without dementia Type: Systematic review Method used for analysis: Narrative synthesis	Design: 8 RCT, 28 NRS Sample size (min–max): 5–300 Medical context: Cognitive impairment Mean–median age (min–max): NR % of female (min–max): NR Geographical settings: USA, Netherlands, Malaysia, Canada, Greece, Germany, Finland, UK, Mexico, France, Brazil, Australia	Name: Social networking technology and ICT training programs Mode of delivery: Technology, face‐to‐face Duration in weeks/months (min–max): 6 weeks–8 months	Social isolation Loneliness
Fu et al. (2022)	Objective: loneliness reduction obtained by remotely delivered intervention for loneliness in older adults. Type: Systematic review Method used for analysis: Meta‐analysis	Design: 4 RCT Sample size (min–max): 35–241 Medical context: None Mean‐median age (min–max): 70%–82% of female (min–max): 66‐100 Geographical settings: UK, China, USA	Name: Remotely delivered intervention Mode of delivery: Telephone, Internet Duration in weeks/months (min–max): 2 weeks–30 weeks	Loneliness
Wister et al. (2021)	Objective: Evaluate technological interventions that have been developed to reduce loneliness and/or social isolation for community‐dwelling older adults. Type: Scoping review Method used for analysis: Qualitative synthesis	Design: 10 RCT, 16 NRS Sample size (min–max): 4–300 Medical context: None Mean‐median age (min–max): 60%–80% of female (min‐max): Geographical settings: Europe, USA, UK, Australia, Canada, Asia	Name: Technological interventions Mode of delivery: Individual, group, Individual and group Duration in weeks/months (min–max): 1 month–18 months	Loneliness Social isolation

Abbreviations: N/A, not applicable; NR, not reported; NRS, non‐randomized studies (include non‐randomized trials and observational studies with or without control group); RCT, randomized controlled trials.

a Control‐placebo.

^b^ For these outcomes, the results were not presented by the review authors in such a way that we would be able to extract information from primary studies.

^c^ This was meta‐analysis of RCT, not observational studies. Since these RCT were also included in Frost et al. (2017); we only considered the observational studies to avoid duplicate.

### 4.4 Characteristics of the interventions in the included reviews

Twenty‐four reviews included interventions that were used to address more than one vulnerability but not in the same primary study. Fourteen reviews (54%, 14/26) included physical activity interventions and technologies such as telemonitoring/tele‐care were used to address frailty, social isolation, and loneliness (Anton et al., 2018; Arantes et al., 2009; Burton et al., 2019; Dedeyne et al., 2017; Frost et al., 2017; Heins et al., 2021; Kelaiditi et al., 2014; Khosravi et al., 2016; Li et al., 2022; Pool et al., 2017; Puts et al., 2017; Shvedko et al., 2018; Snowden et al., 2014; Theou et al., 2011; Wister et al., 2021). Ten reviews included (38%) Interventions combining diet and physical activity, interventions combining diet, physical activity and nutritional advise, and preventive integrated care interventions were deployed to address frailty and social isolation (Anton et al., 2018; Dedeyne et al., 2017; Frost et al., 2017; Ibrahim et al., 2022; Kelaiditi et al., 2014; Liao et al., 2018; Looman et al., 2019; Puts et al., 2017; Theou et al., 2011; Tricco et al., 2022) (Tables 1, 2, 3).

**Table 2:** Effect of non‐pharmacological strategies to address frailty status among community‐dwelling older adults.

Interventions	Author and year	Effect on frailty status	Magnitude of the effect
*Systematic reviews*			
Dietary interventions	Walters et al. (2017)	1 observational study (*n* = 352): non‐significant association between lower vitamin D (serum 25(OH)D) concentration and frailty state transitions	‐
Physical activity interventions	Theou et al. (2011)	1 RCT (*n* = 21): no significant between groups differences	‐
	Frost et al. (2017)	1 RCT (*n* = 69): **Significant differences in Short Physical Performance Battery (SPPB) score changes at 12 weeks between each exercise intervention and control**, but effects not maintained at 24 or 36 weeks.	Not reported
	Burton et al. (2019)	2 trials (*n* = 63)	Not reported
		‐ 1 RCT: no significant between groups difference	
		‐ 1 non‐randomized CT: **intervention significantly improved SPPB score**	
Dietary and physical activity interventions	Dedeyne et al. (2017)	1 RCT (*n* = 131)	
		‐ Postintervention: [**Exercise** + **nutritional supplementation of milk fat globule membrane] significantly improved frailty status compared to nutritional supplementation of proteins**.	OR = 3.12, CI = 1.13; 8.60
		‐ At 3–4 months of follow‐up: [**Exercise** + **nutritional supplementation of proteins]**	OR = 3.64, CI = 1.12; 11.85
		**significantly improved frailty status compared nutritional supplementation of proteins alone**.	OR = 4.67, CI = 1.45; 15.08
		‐ At 3–4 months of follow‐up: [**Exercise** + **nutritional supplementation of milk fat globule membrane] significantly improved frailty status compared to nutritional supplementation of proteins**.	
	Liao et al. (2018)	1 RCT (*n* = 33): **significant difference in favor of the intervention group following protein supplementation and exercise training**	SMD = 0.62, 95% CI = 0.21; 1.03, *p* = 0.003, level of evidence: moderate
Telemonitoring	Frost et al. (2017)	1 RCT (*n* = 87): Comparing telemonitoring to usual care, slightly fewer people transitioned from pre‐frail to non‐frail (9/35 (26%) vs. 12/38 (32%)) or to frail (3/35 (9%) vs. 6/38 (16%)) over 6 months	Not reported
Preventive, integrated care interventions	Looman et al. (2019)	1 RCT (*n* = 241): **Significant improvement in favor of the intervention group**	Not reported
Reablement, Reactivation, Rehabilitation and Restorative Interventions	Sims‐Gould et al. (2017)	1 RCT (*n* = 205): **Significant improvement in favor of the intervention group**	Not reported
Cognitive interventions	Dedeyne et al. (2017)	1 RCT (*n* = 246): **At 9 months follow‐up, intervention significantly improved frailty status compared to control**.	OR = 2.89, CI = 1.07; 7.82 (*p* < 0.01)
Dietary and physical activity and cognitive interventions	Dedeyne et al. (2017)	1 RCT (*n* = 246): **At 9 months follow‐up, [exercise** + **nutritional supplementation of proteins** + **nutritional supplementation of vitamins and minerals** + **cognitive intervention] significantly improved frailty status compared to control**.	OR = 5.00, CI = 1.88; 13.3 (*p* < 0.01)
Physical activity and nutritional advise	Dedeyne et al. (2017)	2 trials (*n* = 197)	45% vs. 27% (*p* < 0.01)
		‐ 1 RCT: [**Exercise** + **nutritional advise] significantly improved frailty status compared to control group**.	
		‐ 1 RCT: no significant between groups differences	
Physical activity and nutritional advise and psychosocial interventions	Dedeyne et al. (2017)	1 RCT (*n* = 117): [**Exercise** + **nutritional advise** + **psychosocial intervention] significantly improved frailty status compared to control group**.	45% vs. 27% (*p* < 0.01)
Dietary and physical activity intervention and nutritional advise	Dedeyne et al. (2017)	1 RCT (*n* = 100): [**Exercise** + **nutritional advise** + **nutritional supplementation of vitamins and minerals] significantly Improved frailty status compared to [nutritional advise** + **nutritional supplementation of vitamins and minerals]**.	‐ Fried frailty criteria:
			1.6 ± 0.9 vs. 3.8 ± 0.3 (*p* < 0.001)
			‐ Edmonton frailty scale: 7.7 ± 2.0 vs 9.3 ± 2.3 (*p* < 0.001)
Medication	Walters et al. (2017)	1 observational study (*n* = 1705):	‐
		‐ Number of medications had no effect on transitions from pre‐frailty to other states (HR pre‐frail‐robust 0.99, pre‐frail‐frail 1.06, pre‐frail‐death 1.02).	
		‐ Drug Burden Index had no effect on transitions from pre‐frailty to other states (HR pre‐frail‐robust 0.90, pre‐frail‐frail 1.03, pre‐frail‐death 1.18)	
BMI	Walters et al. (2017)	1 observational study (*n* = 3018)	
		‐ Pre‐frail men: **normal and overweight BMI was protective against worsening frailty compared with underweight [OR normal 0.47 (95% CI 0.23 to 0.99), overweight 0.36 (95% CI 0.16 to 0.81)]**, but no effect on odds of improving to robust. Obesity had no effect	OR normal vs underweight = 0.47, 95% CI = 0.23; 0.99
		‐ Pre‐frail women: BMI had no effect on frailty transitions	OR overweight vs underweight = 0.36, 95% CI = 0.16; 0.81
Smoking	Walters et al. (2017)	1 observational study (*n* = 3018)	‐
		‐ Pre‐frail men: smoking had no effects on frailty transitions.	
		‐ Pre‐frail women: smoking had no effect on frailty transitions	
Mini Mental State Examination (MMSE)	Walters et al. (2017)	1 observational study (*n* = 3018)	
		‐ Prefrail men: **Higher Mini Mental State Examination (MMSE) score predicted greater likelihood of improvement from prefrail to robust**	OR = 1.10, CI = 1.02; 1.18
		‐ Pre‐frail women: MMSE scores had no effect on frailty transitions	
		‐ In multiple stepwise regression models (male: adjusted for age and stroke, female: adjusted for age, hospital admissions and stroke), **higher MMSE score was a protective factor in both gender**	
*Other reviews*			
Dietary interventions	Kelaiditi et al. (2014)	4 observational studies (*n* = 5819)	
		‐ 1 longitudinal study: **Higher Mediterranean diet (MED) score at baseline was associated with lower risk of developing frailty at the end of the follow‐up**	OR = 0.30, 95% CI = 0.14; 0.66
		‐ 1 cross‐sectional study: **Low risk of being frail in the highest MED score quartile**	OR = 0.26, 95% CI = 0.07; 0.98
		‐ 1 cross‐sectional study: **12 of the 18 mini nutritional assessment items associated with frailty**	Not reported
		‐ 1 cross‐sectional study:	Likelihood of frailty: adjusted OR = 4.7, 95% CI = 1.7; 12.7
		**food insufficiency (albumin, selenium, and carotenoids) associated with higher likelihood of frailty or prefrailty**;	Likelihood of prefrailty: adjusted OR = 2.1, 95% CI = 0.8; 5.8
			Frailty: OR = 1.67, 95% CI = 1.00; 2.82
		**Low 25(OH)D (**<**49.5 nmol/l vs. >84.1 nmol/l) was associated with frailty and prefrailty**.	Prefrailty: OR = 1.54, 95% CI 1.10; 2.15
Physical activity interventions	Kelaiditi et al. (2014)	2 observational studies (n = 246)	
		‐ 1 cross‐sectional study: **Physical activity decreased risk of frailty**.	OR = 0.99, (*p* < 0.001)
		‐ 1 cross‐sectional study: No physical activity measure was associated with frailty	‐
	Puts et al. (2017)	2 trials (n = 543)	
		‐ 1 RCT: **the intervention group compared to control group had significantly greater improvements in 2 of the 3 measures of frailty (the modified PPT, the peak oxygen uptake, but not in ADL function)**.	Not reported
		‐ 1 RCT:	
		**At 12 months the prevalence of frailty in the intervention group was significantly lower than the control group**.	10% vs. 19.1% (*p* < 0.05)
		**At 12 months the mean number of frailty markers decreased significantly more in the intervention group compared to the control group**.	0.48 vs. 0.21 (*p* < 0.05)
		1 cohort study (*n* = 610):	
		**The total frailty score after intervention was lower in the exercise group compared to control group**.	7.1 (SD 4.0) vs. 8.0 (SD 4.8) (*p* < 0.001)
		**During the year, the percentage of older adults newly certified for LTCI (considered frail) was lower in the intervention group compared to control group**.	8% vs 18% (*p* < 0.05)
Dietary and physical activity interventions	Kelaiditi et al. (2014)	1 RCT (*n* = 117): **The intervention group showed higher improvement of frailty compared to the control group after 3 months of intervention**, but not at 6 and 12 months.	45 vs. 27% (*p* = 0.008)
	Puts et al. (2017)	3 trials (n = 348)	
		‐ 1 RCT: **reported at 3 months a significant difference between intervention and**	Not reported
		**control group in the reduction in the number of frail persons**.	
		‐ 1 RCT:	58% (Exercise + milk fat globule membrane) vs. 28% (milk fat globule membrane) (*p* < 0.05)
		**The percentage of non‐frail participants at postintervention was significantly higher in the [Exercise** + **milk fat globule membrane group] than in the milk fat globule membrane group or placebo**.	58% (Exercise + milk fat globule membrane) vs. 30% (placebo) (*p* < 0.05)
		**At 7 months follow‐up it was also significantly greater in the [Exercise** + **milk fat globule membrane group] (46%), and [Exercise** + **placebo] (39%) compared to placebo (15%)**	46% (Exercise+ milk fat globule membrane) vs. 15% (placebo) (*p* < 0.05)
			39% (Exercise + placebo) vs. 15% (placebo) (*p* < 0.05)
		‐ 1 RCT: **the frailty score significantly improved in the intervention group (*p*** < **0.001), 31.4% had a reversal of frailty and none in the control group**.	
		**Similarly, the mean Edmonton Frail scale score also significantly improved in intervention group compared to the control group)**	7.7 vs. 9.3
Home modification	Puts et al. (2017)	1 cohort study (*n* = 547): Non‐significant results of home modifications on frailty prevalence	‐
Prehabilitation physical therapy (physical therapy and exercise and home modification)	Puts et al. (2017)	1 RCT cross‐over trial (*n* = 188): At 7 and 12 months statistically significant differences between IG and CG (decline in the frailty marker in control group)	Not reported
Geriatric assessment	Puts et al. (2017)	3 trials (*n* = 1010)	
		‐ 1 RCT: **Significant reduction in the prevalence of frailty and the number of frailty markers in the intervention group (*p*** < **0.05) at 12 months**	Not reported
		‐ 2 RCT: no significant between groups differences	‐

*Note*: Significant between‐group differences are shown in bold.

Abbreviations: CI, confidence interval; HR, hazard rate; OR, odds ratio; RCT, randomized controlled trial; SMD, standardized mean difference.

Twelve reviews (46%, 12/26) included interventions that addressed frailty through the following approaches: diet (Anton et al., 2018; Dedeyne et al., 2017; Kelaiditi et al., 2014; Walters et al., 2017), reablement, reactivation, rehabilitation and restorative interventions (4R) (Sims‐Gould et al., 2017), cognitive interventions (Dedeyne et al., 2017), interventions combining diet, physical activity and cognitive strategies (Dedeyne et al., 2017; Puts et al., 2017), home modification (Puts et al., 2017), prehabilitation physical therapy (Puts et al., 2017; Theou et al., 2011), interventions combining physical activity and nutritional advise (Dedeyne et al., 2017; Theou et al., 2011), interventions combining physical activity, nutritional advise and psychosocial strategies (Dedeyne et al., 2017), and interventions combining diet, physical activity and hormones (Dedeyne et al., 2017) (Tables 1 and 2, Supporting Information 2). Ten reviews assessed overall frailty status while six reviews addressed specific components of frailty (Fried phenotype).

Seventeen reviews (65%, 17/26) included interventions that addressed either social isolation or loneliness through the following types of interventions: reminiscence therapy (Franck et al., 2016), indoor gardening program (Franck et al., 2016), radio program (Franck et al., 2016), therapeutic social interventions (Hagan et al., 2014), social support (Snowden et al., 2014), social capital intervention (Coll‐Planas et al., 2017), technology (Cohen‐Mansfield & Perach, 2015; Franck et al., 2016; Heins et al., 2021; Ibrahim et al., 2022; Khosravi et al., 2016), volunteering activity (Pool et al., 2017), educational activity (Cohen‐Mansfield & Perach, 2015; Pool et al., 2017), interventions combining physical activity and educational activity (Li et al., 2022; Pool et al., 2017; Shvedko et al., 2018), problem‐based multidisciplinary interventions (Sims‐Gould et al., 2017), restorative home care (Sims‐Gould et al., 2017), shared activities (Sims‐Gould et al., 2017; Tricco et al., 2022), health enhancement program (Tricco et al., 2022) (Tables 1, 3, and 4).

**Table 3:** Effect of non‐pharmacological strategies to address social isolation among community‐dwelling older adults.

Interventions	Authors	Effect on social isolation	Magnitude of the effect
*Systematic review*			
Physical activity	Shvedko et al. (2018)	22 RCTs (*n* = 1625): **A small significant positive effect favoring the experimental condition was found for social functioning**.	SMD = 0.30; 95% CI = 0.12 to 0.49; *p* = 0.001, *I* ^2^ = 63%
		9 RCTs (*n* = 1322): The effect of physical activity for social support was non‐significant.	SMD = − 0.05; 95% CI = −0.19 to 0.10; *p* = 0.53, *I* ^2^ = 34%
		4 RCTs (*n* = 773): The effect of physical activity for social networks was non‐significant.	SMD = − 0.00; 95% CI = −0.28 to 0.27; *p* = 0.99, *I* ^2^ = 68%
		Due to the lack of available data, meta‐analysis for social isolation outcomes was not performed.	
	Heins et al. (2021)	1 RCT (*n* = 30): No significant changes in social interaction of the intervention group versus comparison group.	Not reported
Dietary and physical activity interventions	Dedeyne et al. (2017)	1 RCT (*n* = 112‐161): no significant between groups differences on social involvement.	‐
Dietary and physical activity intervention and nutritional advise	Dedeyne et al. (2017)	1 RCT (*n* = 100): [**Exercise** + **nutritional advise** + **nutritional supplementation of vitamins and minerals] significantly Improved social involvement compared to [nutritional advise** + **nutritional supplementation of vitamins and minerals]**	45.5 ± 9.3 vs. 41.2 ± 8.5
			(*p* < 0.05)
Indoor gardening program	Franck et al. (2016)	1 quasi‐experimental study (*n* = 53): significant increase in socialization for experimental group (*p* < 0.05)	Not reported
Technology: General ICT (computer and/or Internet training and/or use)	Khosravi et al. (2016)	4 trials (*n* = 412)	
		‐ 1 RCT: using Internet associated with lower levels of social isolation	Not reported
		‐ 1 RCT: Significant increase in agreement that using the Internet had made respondents feel less isolated	Not reported
		‐ 1 quasi‐experimental study: increased level of social inclusion after the intervention	Not reported
		‐ 1 RCT: no significant between groups differences	‐
		1 longitudinal survey (*n* = 20): Satisfaction in the amount of contact with others increased significantly	Not reported
		1 longitudinal survey (*n* = 3075): Internet use reduced isolation	Not reported
		1 cross‐sectional survey (*n* = 2018): Spending time behind the computer (passive activities) were not associated with social connectedness	**‐**
	Heins et al. (2021)	1 CCT (*n* = 83): No statistically significant differences in social support between groups	Not reported
		2 NRS (*n* = 73): No significant differences in any social outcome measures	
Technology: Social network sites	Khosravi et al. (2016)	1 cross‐sectional survey (*n* = 268): **Using social network sites increased social satisfaction**	Not reported
	Heins et al. (2021)	1 CCT (*n* = 43): No significant differences in social support between groups.	Not reported
Technology: Robotics	Khosravi et al. (2016)	1 Experimental study; interview (*n* = 12): **Reduction of social isolation**	Not reported
Technology: Reminiscing therapy	Heins et al. (2021)	1 RCT (*n* = 8): outcomes related to social participation significantly improved at mid‐term follow‐up. Difference not maintained at 12 weeks.	individual MM group versus the control group
			(*t* = 2.84, *p* = 0.005)
Preventive, integrated care for community‐dwellingfrail older people	Looman et al. (2019)	11 trials (*n* = 6370) on social functioning	
		‐ 3 RCTs: **significant outcome in favor of the intervention**	Not reported
		‐ 2 RCTs: no significant between groups differences	‐
		‐ 2 cluster RCTs: no significant between groups differences	‐
		‐ 4 controlled before‐and‐after studies: NS no significant between groups differences	‐
		3 trials (*n* = 829) on social support	
		‐ 2 RCTs: no significant between groups differences	‐
		‐ 1 cluster RCT: no significant between groups differences	‐
Volunteering activity	Cohen‐Mansfield and Perach (2015)	6 trials (*n* = 760)	
		‐ 1 RCT: **At postintervention, the amount of interpersonal contact significantly increased among intervention participants when compared to waitlisted controls**.	Not reported
		‐ 1 RCT: no significant between groups differences	‐
		‐ 1 matched controlled trial: no significant between groups differences	‐
		‐ 3 not randomized, not controlled: no significant between groups differences	‐
Restorative home care model	Sims‐Gould et al. (2017)	1 RCT (*n* = 205): no significant between groups differences	‐
Shared activities (group interventions)	Cohen‐Mansfield and Perach (2015)	1 RCT (*n* = 40): **Amount of social interaction, and social initiative significantly increased in the intervention group (visual art discussions) compared to the control group at postintervention and at 4‐month follow‐up**.	Not reported
Physical activity advise	Heins et al. (2021)	1 CCT (*n* = 21): Failed to demonstrate statistically significant intervention effects on social participation outcomes	Not reported
Health enhancement program?	Tricco et al. (2022)	1 RCT (*n* = 312) on social network: After 6 months of follow‐up, they found no statistically significant difference between the intervention and control groups	Mean difference: 0.038
			(95% CI = –0.25 to 0.3)
			Mean difference: 0.102
		1 RCT (*n* = 312) on social satisfaction: After 6 months of follow‐up, they found no statistically significant difference between the intervention and control groups	(95% CI = –0.35 to 0.55)
*Other reviews*			
Therapeutic social interventions	Hagan et al. (2014)	2 trials (*n* = 565)	
		‐ 1 before‐and‐after study: **following mentoring service and community activities (one‐to‐one intervention), there were significant improvements in social support at 12 months (p** = **0.02)**	Not reported
		‐ 1 controlled trial or RCT: no significant between groups differences	‐
Volunteering activity	Pool et al. (2017)	2 trials (*n* = 830) on social activity	
		‐ 1 RCT: “**Number of adults you could turn to for help” increased in intervention group and decreased in control group with a significant difference between groups**	(from 5.3 to 6.2) in intervention vs. (from 5.8 to 4.3) in control, z(*p* = 0.03)
		‐ 1 RCT: no significant between groups differences	‐
Physical activity	Pool et al. (2017)	1 quasi‐experimental study (*n* = 39): **social support increased after intervention**	Mean change (SD): 0.4 (0.7) (baseline score 4.9—score after 12 weeks 5.4 (*p* = 0.008))
Physical activity and educational activity	Pool et al. (2017)	1 RCT (*n* = 178): **Social Provision Score (social functioning) increased from 52.55 (SD; 7.90) to 59.12 (SD: 9.48) in intervention group and from 52.69 (SD: 8.63) to 54.06 (SD: 9.08) in control group**.	LS mean group differences =5.20, 95% CI = 2.12; 8.19 (*p* = 0.0008)
		1 pretest–posttest study design (*n* = 402): **significant increase of social activity score at 6‐week posttest**.	Mean difference in change on social activity score; 0.62, 6‐week posttest (*p* ≤ 0.05)
Technology: Digital storytelling	Ibrahim et al. (2022)	1 NRS (*n* = 8): Participants had increase in social connectedness and network size	‐
Shared activities (group interventions)	Ibrahim et al. (2022)	2 RCT (n = 478)	
		‐ 1 RCT (*n* = 223): Social integration increased in the experimental group.	
		‐ 1 RCT (*n* = 235): No difference in social network.	
		NRS (*n* = 488)	
		‐ 1 NRS (*n* = 168): No difference between experimental and control groups for support satisfaction and the number of social ties built with other residents of the building.	
		‐ 1 NRS (*n* = 320): No significant differences between experimental a learning control groups for social participation.	
		‐ 1 NRS (*n* = 38): Significant increase in social participation.	
		‐ 2 NRS (*n* = 103): Significant improvement in social integration after the intervention.	
		‐ 1 NRS (*n* = 120): Increase in social contact.	

*Note*: Significant between‐group differences are shown in bold. Therapeutic social interventions include: Community Connections' group, Mindfulness Based Stress Reduction Program, Day center attendance, LUSTRE 6‐week group program (social program focusing on positive self‐management and well‐being), Friendship enrichment program, Psychosocial group intervention, Senior Companion Program Befriending scheme (one‐to‐one intervention), Community‐based mentoring Service (one‐to‐one intervention), Mentoring service + community activities (one‐to‐one intervention).

Abbreviations: CI, confidence interval; CT, controlled trial; NS, no significant results; RCT, randomized controlled trial; SD, standard deviation; SMD, standardized mean difference.

Nineteen reviews included studies with single component interventions (Anton et al., 2018; Arantes et al., 2009; Burton et al., 2019; Cohen‐Mansfield & Perach, 2015; Fu et al., 2022; Franck et al., 2016; Heins et al., 2021; Ibrahim et al., 2022; Kelaiditi et al., 2014; Khosravi et al., 2016; Li et al., 2022; Liao et al., 2018; Pool et al., 2017; Puts et al., 2017; Shvedko et al., 2018; Snowden et al., 2014; Theou et al., 2011; Tricco et al., 2022; Wister et al., 2021) and 19 reviews included studies with multi‐component interventions (Anton et al., 2018; Arantes et al., 2009; Burton et al., 2019; Cohen‐Mansfield & Perach, 2015; Dedeyne et al., 2017; Franck et al., 2016; Frost et al., 2017; Hagan et al., 2014; Heins et al., 2021; Kelaiditi et al., 2014; Li et al., 2022; Liao et al., 2018; Pool et al., 2017; Puts et al., 2017; Shvedko et al., 2018; Sims‐Gould et al., 2017; Snowden et al., 2014; Theou et al., 2011; Tricco et al., 2022). Single component interventions were diet (Anton et al., 2018; Kelaiditi et al., 2014; Liao et al., 2018; Shvedko et al., 2018), physical activity intervention (Anton et al., 2018; Burton et al., 2019; Frost et al., 2017; Kelaiditi et al., 2014; Li et al., 2022; Liao et al., 2018; Shvedko et al., 2018; Snowden et al., 2014; Theou et al., 2011; Wister et al., 2021), reminiscence therapy (Li et al., 2022; Franck et al., 2016), social clubs (Franck et al., 2016; Tricco et al., 2022), playing video games (e.g., Wii) (Franck et al., 2016; Heins et al., 2021; Khosravi et al., 2016; Wister et al., 2021), radio program (Franck et al., 2016), educational activity (Anton et al., 2018; Cohen‐Mansfield & Perach, 2015; Pool et al., 2017), technology (Cohen‐Mansfield & Perach, 2015; Heins et al., 2021; Ibrahim et al., 2022; Khosravi et al., 2016; Wister et al., 2021), and home modification (Puts et al., 2017). Multicomponent interventions were multicomponent exercise programs, home care services, individualized reablement programs, individualized home exercise programs or interventions combining two or more of the following elements: diet, physical activity, pain management, gardening activities, education, psychotherapy, cognition training, volunteering activity, social interactions, social support, reablement, reactivation, rehabilitation and restorative interventions (4R), prehabilition therapy, home modification, geriatric assessment, technology, hormone, specific therapy techniques. Three reviews did not provide enough details about intervention components (Coll‐Planas et al., 2017; Hagan et al., 2014; Looman et al., 2019). Smith et al. (2019) did not find any interventions that met the eligibility of the review. More details about the components of the interventions are presented in Supporting Information 3.

Among the 26 included reviews (excluding Smith et al. (2019) that did not find any eligible studies), 24 reported on duration of interventions. the minimum duration was 1 week (Khosravi et al., 2016) and the maximum duration was 120 months (Ibrahim et al., 2022) (Table 1). Two reviews did not report on duration of interventions (Hagan et al., 2014; Looman et al., 2019).

### 4.5 Primary outcomes measures

The Fried phenotype was the most frequently (67%, 8/12) reported instrument to measure frailty status (Anton et al., 2018; Arantes et al., 2009; Burton et al., 2019; Frost et al., 2017; Liao et al., 2018; Puts et al., 2017). Other instruments included: modified Fried phenotype (Kelaiditi et al., 2014; Puts et al., 2017), Frailty Index (Liao et al., 2018), Short Physical performance Battery (SPPB) (Burton et al., 2019; Frost et al., 2017; Liao et al., 2018; Sims‐Gould et al., 2017; Theou et al., 2011), Tinetti/Gill criteria (Puts et al., 2017), Japanese Frailty checklist (Puts et al., 2017), Chinese Canadian Study of Health and Aging Clinical Frailty Scale Telephone Version (Puts et al., 2017), Edmonton Frailty Scale (Puts et al., 2017), Barthel index (Puts et al., 2017), Physical performance test (Dedeyne et al., 2017), Modified Chin A Paw frailty definition (Dedeyne et al., 2017), Frailty Instrument for Primary Care of the Survey of Health (Dedeyne et al., 2017), Ageing, and Retirement in (SHARE‐FI) (Dedeyne et al., 2017), and non‐validated definitions of frailty (Frost et al., 2017; Looman et al., 2019).

There was great variety in the measurement tools used to assess social isolation and its related dimensions. Instruments included: Lubben Social Network Scale (LSNS) (Franck et al., 2016; Heins et al., 2021), two questions on social isolation developed by authors (Franck et al., 2016), Internally developed social support scale (Hagan et al., 2014), Medical Outcomes Study (MOS) Social Support Survey (Hagan et al., 2014; Heins et al., 2021; Shvedko et al., 2018), social provisions scale (Hagan et al., 2014; Heins et al., 2021), The Multidimensional Scale of Perceived Social Support (Pool et al., 2017; Shvedko et al., 2018; Heins et al., 2021), Duke Social Support Index (Heins et al., 2021; Sims‐Gould et al., 2017; Tricco et al., 2022; Wister et al., 2021), the Patient Reported Outcome Measurement Information System (PROMIS)—Social Isolation (six‐item) (Wister et al., 2021), Social activities from the RAND Social Health Battery (one item on social participation) (Cohen‐Mansfield & Perach, 2015), Items from the Older American Resources and Services (OARS) social resource rating scale (custom item on satisfaction with socialization) (Cohen‐Mansfield & Perach, 2015), Items tapping daily activities and social contact (Cohen‐Mansfield & Perach, 2015), and Social Provisions Scale (Heins et al., 2021), Multidimensional Scale of Perceived Social Support (MSPSS) (Shvedko et al., 2018; Heins et al., 2021), Self‐report of social Interaction and social network (Cohen‐Mansfield & Perach, 2015; Heins et al., 2021).

Regarding measurement of loneliness, University of California Los Angeles Loneliness Scale (UCLA) was the most frequently reported instrument (71%, 10/14) (Cohen‐Mansfield & Perach, 2015; Coll‐Planas et al., 2017; Franck et al., 2016; Fu et al., 2022; Hagan et al., 2014; Heins et al., 2021; Khosravi et al., 2016; Pool et al., 2017; Shvedko et al., 2018; Wister et al., 2021). Other instruments included: De Jong Gierveld Loneliness scale (DJGLS) (Fu et al., 2022), De Jong and Kamphuis Loneliness Scale, Philadelphia Geriatric Center Morale Scale (PGCMS) (Cohen‐Mansfield & Perach, 2015), Lonely Dissatisfaction subscale (Cohen‐Mansfield & Perach, 2015), Three‐item loneliness scale (Heins et al., 2021) and Medical Outcome Social Support Survey (MOSS) (Heins et al., 2021), participants self‐report of the impact of the intervention on their loneliness (Cohen‐Mansfield & Perach, 2015), Emotional/Social Loneliness Inventory (15 paired items) (Cohen‐Mansfield & Perach, 2015).

Two reviews did not report on the instruments used to measure the outcomes of interest (Looman et al., 2019; Snowden et al., 2014). None of the reviews reported on the psychometric characteristics of the instruments (Supporting Information 4).

### 4.6 Effect of the interventions

#### 4.6.1 Interventions addressing frailty

In Table 2 we report the effects of the interventions on frailty status and on frailty components (gait speed, grip strength, body weight, physical activity, exhaustion) (Fried et al., 2001) (Supporting Information 2).

Regarding frailty status, a systematic review reported that exercise combined with nutritional supplementation of milk fat globule membrane had highest odds of frailty reversal compared to nutritional supplementation of proteins alone at postintervention (odds ratio [OR] = 3.12, confidence interval [CI] = 1.13‐8.60, 1 RCT, *n* = 131) and at 3‐4 months follow‐up (OR = 4.67, CI = 1.45–15.08, 1 RCT, *n* = 131). It also reported that exercise combined with nutritional supplementation of proteins had highest odds of frailty reversal compared with nutritional supplementation of proteins alone at 3–4 months follow‐up (OR = 3.64, CI = 1.12–11.85, 1 RCT, *n* = 131) (Dedeyne et al., 2017). A systematic review with meta‐analysis reported a statistically significant difference in favor of the intervention group following protein supplementation and exercise training (standardized mean difference [SMD] = 0.62, 95% CI = 0.21–1.03, *p* = 0.003, 1 RCT, *n* = 33, level of evidence: moderate) (Liao et al., 2018). A scoping review reported evidence of a positive effect of physical activity on preventing frailty, reducing level of frailty or frailty markers on the two RCTs (*n* = 543) and the cohort study (*n* = 610) that assessed this intervention. It also reported evidence of a positive effect of diet combined with physical activity on preventing frailty and reducing the level of frailty on the three RCTs (*n* = 348) that assessed this intervention (Puts et al., 2017). The other reviews did not provide enough evidence in favor of an improvement of frailty status following the other types of intervention.

Regarding factors associated with frailty status, evidence from observational studies shown that normal and overweight Body Mass Index, higher Mini Mental State Examination (MMSE) score were protective against worsening frailty or predicted greater likelihood of improvement from prefrail (prefrail being defined as presenting one or two of the five criteria of Fried's frailty phenotype) to robust among prefrail men (Walters et al., 2017). Higher Mediterranean diet (MED) score was associated with lower risk of frailty whereas food insufficiency (albumin, selenium, and carotenoids) and lower vitamin D levels were associated with higher risk of frailty or prefrailty (presenting one or two of the five criteria of Fried's frailty phenotype) (Kelaiditi et al., 2014).

Regarding walking speed, the results from systematic reviews and other type of reviews were conflicting and did not provide consistent evidence in favor of an improvement of this outcome following any type of intervention.

With respect to grip strength, a systematic review with meta‐analysis reported a significant improvement of this outcome in favor of an intervention combining protein supplementation and exercise training (SMD = 0.18, 95% CI = 0.01–0.36, *p* = 0.04, *I* ^2^ = 24%, 7 RCTs, level of evidence: strong) (Liao et al., 2018). The results from the other reviews did not provide evidence in favor of an improvement of grip strength following the other types of intervention.

For body weight, a systematic review with meta‐analysis reported a significant improvement of this outcome in favor of an intervention combining protein supplementation and exercise training (SMD = 0.58, 95% CI = 0.25–0.91, *p* = 0.0006, *I* ^2^ = 65%, 6 RCTs, level of evidence: moderate) (Liao et al., 2018). The results from the other reviews were conflicting and did not provide consistent evidence in favor of an improvement of body weight following the other types of intervention.

Regarding physical activity, a systematic review reported that the two included trials (*n* = 501) that tested physical activity interventions shown an improvement of this outcome following the intervention (Theou et al., 2011). The results from the other reviews did not provide enough evidence in favor of an improvement of physical activity following the other types of intervention.

#### 4.6.2 Interventions addressing social isolation

A systematic review with meta‐analysis reported a small significant positive effect in favor of physical activity interventions on social functioning (SMD = 0.30; 95% CI = 0.12–0.49; *p* = 0.001, *I* ^2^ = 63%, 22 RCTs, *n* = 1625) (Shvedko et al., 2018). Another systematic review reported that three of four included trials (*n* = 412) and two longitudinal surveys (*n* = 3095) shown a reduction of social isolation, an increase of social inclusion and an increase of satisfaction in the amount of contact with others following Internet use (Khosravi et al., 2016). The results from the other reviews were conflicting or did not provide enough evidence in favor of an improvement of social isolation following the other types of intervention (Table 3).

#### 4.6.3 Interventions addressing loneliness

A network meta‐analysis of 3 RCTs and 15 NRS (*n* = 1523) on physical activity and psychosocial interventions from found an insignificant improvement in loneliness (Hedge's *g* = 1.09; 95% CI = 0.57 to 1.62) (Li et al., 2022). Similarly, a meta‐analysis of four RCTs (*n* = 165) of remotely delivered interventions aimed at reducing loneliness found no significant effect (*p* > 0.05; SMD = 0.18 [95% CI = −0.03 to 0.40]; *I* ^2^ < 50%). The results from the other reviews were conflicting or did not provide enough evidence in favor of an improvement of loneliness following the other types of intervention (Table 4).

**Table 4:** Effect of non‐pharmacological strategies to address loneliness among community‐dwelling older adults.

Interventions	Authors	Effect on loneliness	Magnitude of the effect
*Systematic reviews*			
Reminiscence therapy	Franck et al. (2016)	1 RCT (*n* = 130): **significant difference between control group and experimental group on follow‐up test, with experimental group showing improvement from moderate to mild loneliness**	(*z* = −27.26, *p* < 0.0001) vs. (z = −22.75, *p* < 0.0001)
Indoor gardening program	Franck et al. (2016)	1 quasi‐experimental study (*n* = 53): **significant reduction in loneliness**	Not reported
		**for experimental group (*p*** < **0.05)**	
Technology: General ICT (computer and/or Internet training and/or use)	Khosravi et al. (2016)	7 trials (*n* = 542)	
		‐ 1 RCT: **Using Internet associated with lower levels of loneliness**	Not reported
		‐ 3 quasi‐experimental studies: **the intervention decreased feelings of loneliness**	Not reported
		‐ 1 RCT: No significant between groups differences. But there was a slightly greater tendency towards less loneliness in the intervention group	‐
		‐ 2 RCTs: no significant between groups differences	Not reported
		1 longitudinal survey (*n* = 3075): **Internet use reduced loneliness**	Not reported
		4 cross‐sectional surveys (*n* = 1568): **Internet use decreases (or correlates with less) loneliness**	
	Heins et al. (2021)	1 Controlled trial (*n* = 300): significant changes between the two groups in the domain of loneliness identified at mid‐term follow‐up. Not maintained at 6 months.	*b* = 1.72, *p* < 0.04
Technology: Social network sites	Khosravi et al. (2016)	1 longitudinal survey (*n* = 440): **Social network sites users reported more loneliness than nonusers**	Not reported
		1 cross‐sectional survey (*n* = 268): **Using social network sites reduced loneliness**	Not reported
		2 cross‐sectional surveys (*n* = 768): There was no relationship between using social network sites and loneliness	‐
		1 qualitative study (depth interviews) (*n* = 6): **Using social network sites reduced loneliness**	Not applicable
Technology: Robotics	Khosravi et al. (2016)	4 trials (*n* = 89)	
		‐ 1 RCT: **Significant decreases in loneliness over the period of the trial**	Not reported
		‐ 1 Experimental study; Longitudinal: **Participants felt a sense of companionship with the agent; using the system reduced perceived loneliness**	Not reported
		‐ 1 Intervention study: **Participants who interacted with the agent longer reported feeling less lonely at the end of the study**	Not reported
		‐ 1 quasi‐experimental study: no significant between groups differences	‐
		1 Case study; Interaction with robot in 20 sessions (*n* = 3): **Decreased of stress and loneliness**	Not applicable
Physical activity advise	Heins et al. (2021)	1 CCT (*n* = 21): Loneliness decreased statistically significant in the control group, not in the intervention group. No significant group differences.	Paired *t*(7) = 2.74, *p* < 0.05
Technology: Video game Wii	Khosravi et al. (2016)	1 RCT (*n* = 36): **Wii group had lower loneliness at post‐test, TV group higher loneliness post‐test (*F* (2,30) = 6.24, *p*** < **0.005)**	Not reported
Technology: Personal Reminder Information and Social Management System (Special software designed for seniors)	Khosravi et al. (2016)	1 RCT (*n* = 300): **Use of software reduced loneliness**	Not reported
Technology: Asynchronous peer‐led support chat room(Koffee Klatch)	Khosravi et al. (2016)	1 RCT (*n* = 183): no significant between groups differences	
Technology: Tele‐care	Khosravi et al. (2016)	2 intervention studies (*n* = 201): **Level (feeling) of loneliness (significantly) decreased after intervention**	Not reported
	Fu et al. (2022)	4 RCT (*n* = 165) (meta‐analysis): The effectiveness was not found for participants living as community dwellers.	SMD = 0.18 [95% CI = −0.03 to 0.40]; *I* ^2^ < 50%; *p* > 0.05
Technology: 3D virtual environment	Khosravi et al. (2016)	1 intervention study (*n* = 7): **Lower levels of loneliness across participants**	‐
Technology: Radio program	Franck et al. (2016)	1 quasi‐experimental study (*n* = 24): No change in loneliness (*z* = −1.27, *p* = 0.2)	‐
Technology: sensory technological aids (one‐on‐one interventions)	Cohen‐Mansfield and Perach (2015)	1 non‐randomized controlled trial (*n* = 140): The intervention group showed a non‐significant decrease in loneliness when comparing baseline to 6‐months follow‐up.	‐
Educational activity	Cohen‐Mansfield and Perach (2015)	12 trials (*n* = 2796)	
		‐ 1 randomized (for one intervention group only), controlled trial: **Both forms of intervention resulted in significantly less loneliness in a group by time interaction analysis. Follow‐ups were 2‐ and 12‐month postintervention**.	Not reported
		‐ 1 not randomized controlled trial: **At postintervention, the difference in reduction of loneliness between the experimental and control group was significant**.	Not reported
		‐ 1 not randomized, not controlled trial: **Participants experienced significant reductions in loneliness at postintervention compared to baseline**. The greatest drop in loneliness was seen in low‐income ethnic minorities and minorities with high levels of education.	Not reported
		‐ 6 RCTs: no significant between groups differences	
		‐ 2 not randomized controlled trial: no significant between groups differences	‐
		‐ 1 not randomized, not controlled trial: no significant between groups differences	‐
Physical activity interventions	Shvedko et al. (2018)	Due to the lack of available data, meta‐analysis for loneliness was not performed	‐
	Snowden et al. (2015)	1 RCT (*n* = 32): no significant between groups differences	‐
	Cohen‐Mansfield and Perach (2015)	1 RCT with 2 intervention (exercise) groups (*n* = 174): In both conditions, loneliness significantly decreased at the end of the intervention but significantly increased at 6‐month follow‐up.	‐
Physical activity and psychosocial interventions	Li et al. (2022)	3 RCT + 15 NRS (*n* = 1523) (network meta‐analysis): Group that received the intervention had Insignificant improvement in loneliness.	Hedge's *g* = 1.09 95% CI = 0.57 to 1.62
Problem‐based multidisciplinary intervention targeting vulnerable older adults at home	Sims‐Gould et al. (2017)	1 RCT (*n* = 151): no significant between groups differences	‐
Social capital intervention	Coll Planas et al. (2017)	8 trials (*n* = 1213)	
		‐ 2 RCTs: **statistically significant effects (*p*** < **0.05) favouring the social capital intervention**	Not reported
		‐ 6 RCTs: no significant between groups differences	‐
Social support	Snowden et al. (2015)	2 trials (*n* = 313)	
		‐ 1 RCT: no significant between groups differences	‐
		‐ 1 pretest‐posttest study design: no significant between groups differences	‐
Shared activities (group interventions)	Cohen‐Mansfield and Perach (2015)	2 trials (*n* = 346)	
		‐ 1 RCT: no significant between groups differences	‐
		‐ 1 not randomized CT: no significant between groups differences	‐
	Tricco et al. (2022)	3 NRS (*n* = 2031)	*F*(1,126) = 3.08; *p* = 0.08
		‐ 1 NRS (*n* = 166): lower loneliness in Chorale intervention compared to usual group after 12 months of follow up, but no statistically significant difference observed.	(Baseline mean: 8.7 [SD: 3.2] and follow‐up mean: 7.0 [SD: 3.8])
		‐ 1 NRS (*n* = 21): ElderHelp Concierge Club intervention had no significant change after 6 months of follow‐up.	Mean difference: −0.18
		‐ 1 NRS (*n* = 1844): Urban Health Centers Europe had a small positive effect on loneliness when compared with usual care.	95% CI = −0.35 to −0.02
*Other reviews*			
Therapeutic social interventions^a^	Hagan et al. (2014)	4 trials (*n* = 677)	
		‐ 1 controlled trial or RCT: **Statistically significant decreases in those who completed the Mindfulness Based Stress Reduction Program compared with a control group, who actually had a slight increase in loneliness**.	Intervention group mean reduced from 42.35 to 37.40, compared with the control group's increase from 38.40 to 40.75; (*p* = 0.008)
		‐ 3 controlled trials or RCTs: no significant between groups differences	‐
		1 pilot study (*n* = 764): this pilot study could not adequately evaluate the programme's potential impact on loneliness.	‐
		1 cross‐sectional study (*n* = 817): no statistically significant differences between 417 day‐centre attendees and 400 older people who did not attend in terms of their reported loneliness.	‐
		1 evaluation (mixed methodology) (*n* = 66): examination of a Senior Companion Programme (Befriending scheme, one‐to‐one intervention), which matched volunteers with older individuals, measured loneliness on one occasion and reported a below average scoring for the participants (a mean of 31, the author stating that a typical score for those between 60 and 80 years of age being between 32 and 37)	Not applicable
Educational activity	Pool et al. (2017)	1 pre‐experimental one‐group pretest–posttest design (*n* = 339): **loneliness decreased**.	from a mean score of 8.64 (SD 0.10) to 7.86 (SD 0.09) (*t* = 9.20, *df* = 329; *p* < 0.001)
Physical activity	Wister et al. (2021)	1 RCT (*n* = 21): Not fully effective in reducing loneliness based on an assessment of the study results (e.g., loneliness decreased initially following baseline but increased at follow‐up)	Not reported
Physical activity and psychosocial interventions	Ibrahim et al. (2022)	2 RCT (*n* = 931): No significant difference in loneliness.	‐
Technology: Tele‐care	Wister et al. (2021)	1 NRS (*n* = 130): interventions were effective in reducing loneliness	Not reported
		1 NRS (71): Not fully effective in reducing loneliness based on an assessment of the study results (e.g., loneliness decreased initially following baseline but increased at follow‐up)	Not reported
Technology: Video games	Wister et al. (2021)	1 RCT (*n* = 35): intervention was effective in reducing loneliness	Not reported
		2 NRS (*n* = 307):	
		‐ 1 NRS (*n* = 277): intervention was effective in reducing loneliness	Not reported
		‐ 1 NRS (n = 30): intervention was not effective in reducing loneliness	Not reported
Technology: General ICT (computer and/or Internet training and/or use)	Ibrahim et al. (2022)	1 RCT White (*n* = 100): No difference in loneliness	‐
	Wister et al. (2021)	5 RCT (*n* = 499): interventions were not effective in reducing loneliness	Not reported
		4 NRS (*n* = 319): interventions were effective in reducing loneliness	Not reported
		2 NRS (*n* = 51): interventions were not effective in reducing loneliness	
Social support	Ibrahim et al. (2022)	2 RCT (*n* = 631): No significant improvement in loneliness	Not reported
Technology: Personal Reminder Information and Social Management System (Special software designed for seniors)	Wister et al. (2021)	1 RCT (*n* = 300): intervention was effective in reducing loneliness.	Not reported
Shared activities (group interventions)	Ibrahim et al. (2022)	5 RCT (*n* = 1090)	Not reported
		‐ 4 RCT (*n* = 700): No difference in loneliness between intervention and control group.	
		‐ 1 RCT (*n* = 390): Significant improvements in reduced loneliness in the intervention group compared to the control group	
		3 NRS (*n* = 286)	
		‐ 2 NRS (*n* = 158): Significant decrease in loneliness.	
		‐ 1 NRS (*n* = 128): No significant difference between intervention and control groups for loneliness.	

*Note*: Significant between‐group differences are shown in bold. Therapeutic social interventions include: Community Connections' group, Mindfulness Based Stress Reduction Program, Day center attendance, LUSTRE 6‐week group program (social program focusing on positive self‐management and well‐being), Friendship enrichment program, Psychosocial group intervention, Senior Companion Program Befriending scheme (one‐to‐one intervention), Community‐based mentoring Service (one‐to‐one intervention), Mentoring service + community activities (one‐to‐one intervention).

Abbreviations: RCT, randomized controlled trial; SD, standard deviation.

#### 4.6.4 Effect of interventions on adverse health outcomes, health services use and cost

Eight reviews (Arantes et al., 2009; Burton et al., 2019; Coll‐Planas et al., 2017; Dedeyne et al., 2017; Franck et al., 2016; Ibrahim et al., 2022; Looman et al., 2019; Theou et al., 2011) reported on the effect of the interventions on adverse health outcomes and cost. The results from these reviews were conflicting or did not provide sufficient information to describe the effects (e.g., no effect size or statistical measures) of the interventions on adverse health outcomes or health services use (Supporting Information 7).

Regarding costs, a systematic review reported that a physical activity intervention helped reduce the expenditure on institutionalization and medical visits (one trial, *n* = 104) (Arantes et al., 2009). Another systematic review reported that physical activity reduced paid caregiver support (1 RCT, *n* = 186) (Burton et al., 2019). Ibrahim et al (Ibrahim et al., 2022) reported that group interventions significantly reduced healthcare costs (1 RCT, *n* = 390). There was no evidence of a positive effect of preventive, integrated care interventions on costs (Looman et al., 2019) (Supporting Information 7).

### 4.7 Subgroup analyses

We did not find any reviews that conducted subgroup analyses between participants of LMICs and high‐income countries, individuals living in urban settings versus rural settings, and men versus women.

### 4.8 Methodological quality assessment of the reviews

The quality of the reviews ranged from low to critically low indicating that all the included reviews had at least one critical flaw. All the reviews included the components of PICO in the research questions, described the inclusion criteria and the selection of the study designs for inclusion, and used a comprehensive literature search strategy. For five reviews (25%), it was not clear if the authors performed study selection with two independent researchers (Anton et al., 2018; Cohen‐Mansfield & Perach, 2015; Hagan et al., 2014; Kelaiditi et al., 2014; Snowden et al., 2014). For nine reviews (45%), it was not clear if the authors performed data extraction in duplicate (Anton et al., 2018; Arantes et al., 2009; Burton et al., 2019; Cohen‐Mansfield & Perach, 2015; Hagan et al., 2014; Kelaiditi et al., 2014; Shvedko et al., 2018; Snowden et al., 2014). None of the review authors provided a list of excluded studies and justified the exclusions. Ten reviews (37%) did not use a satisfactory technique for assessing the risk of bias in individual RCTs and NRS included in the review (Anton et al., 2018; Cohen‐Mansfield & Perach, 2015; Hagan et al., 2014; Ibrahim et al., 2022; Kelaiditi et al., 2014; Khosravi et al., 2016; Li et al., 2022; Theou et al., 2011; Walters et al., 2017; Wister et al., 2021). However, in five of the seven reviews that conducted meta‐analyses, the review authors assessed the potential impact of risk of bias in individual studies on the results of the meta‐analysis (Burton et al., 2019; Liao et al., 2018; Shvedko et al., 2018; Walters et al., 2017). For 18 reviews (67%), the review authors reported any potential sources of conflict of interest, including any funding they received for conducting the review (Supporting information 5) (Anton et al., 2018; Burton et al., 2019; Coll‐Planas et al., 2017; Dedeyne et al., 2017; Frost et al., 2017; Fu et al., 2022; Heins et al., 2021; Ibrahim et al., 2022; Kelaiditi et al., 2014; Liao et al., 2018; Pool et al., 2017; Puts et al., 2017; Shvedko et al., 2018; Snowden et al., 2014; Theou et al., 2011; Tricco et al., 2022; Walters et al., 2017; Wister et al., 2021).

### 4.9 Methodological quality of the primary studies in the reviews

Eighteen of the 21 systematic reviews assessed the methodological quality of the included studies. For two systematic reviews, it was unclear whether the authors assessed the quality or not (Cohen‐Mansfield & Perach, 2015; Khosravi et al., 2016). One systematic review did not assess the quality because there were no studies found that met the eligibility criteria (Smith et al., 2019). Two of the other type of reviews assessed the methodological quality (Pool et al., 2017; Puts et al., 2017). Cochrane risk of bias tools were the most frequently used by the reviews to assess methodological quality of the included studies (Burton et al., 2019; Coll‐Planas et al., 2017; Frost et al., 2017; Fu et al., 2022; Looman et al., 2019; Shvedko et al., 2018; Sims‐Gould et al., 2017; Tricco et al., 2022). Other tools were: the PEDro scale (Arantes et al., 2009; Liao et al., 2018), the Downs and Black checklist for methodological quality assessment of randomized and nonrandomized studies of healthcare interventions (Franck et al., 2016), the methodological index for nonrandomized studies (MINORS) (Dedeyne et al., 2017), the Jadad methodological quality criteria scale (Dedeyne et al., 2017), the Newcastle–Ottawa Scale (Pool et al., 2017; Walters et al., 2017), the Mixed Methods Appraisal Tool (MMAT) (Puts et al., 2017), and The Joanna Briggs Institute Critical Appraisal Checklist for Randomized Controlled Trials (Li et al., 2022). For most of the reviews, the quality of the included studies was rated as moderate to high (Arantes et al., 2009; Burton et al., 2019; Dedeyne et al., 2017; Franck et al., 2016; Heins et al., 2021; Liao et al., 2018; Looman et al., 2019; Pool et al., 2017; Puts et al., 2017; Shvedko et al., 2018; Tricco et al., 2022; Theou et al., 2011; Walters et al., 2017). For four reviews, most of the studies had high or unclear risk of bias (Coll‐Planas et al., 2017; Frost et al., 2017; Fu et al., 2022; Li et al., 2022) (Supporting Information 6).

### 4.10 Certainty of evidence from primary studies in the reviews

Only one systematic review graded the level of evidence for each outcome of interest. The level of evidence was moderate for frailty status, conflicting for walking speed, strong for grip strength, moderate for body weight and conflicting for physical activity (Liao et al., 2018). Snowden et al involved a panel of experts who rated the certainty in social support to reduce loneliness as insufficient due to lack of studies (Snowden et al., 2014) (Supporting Information 6).

## 5 DISCUSSION

In this umbrella review, we identified systematic reviews that suggested that programs focused on protein supplementation combined with physical activity may improve frailty status, and frailty components like grip strength and body weight. They also suggested that physical activity alone or in combination with diet may prevent frailty and may improve social functioning.

The quality of the reviews ranged from low to critically low which is comparable to other assessments of reviews of interventions addressing geriatric care (Conneely et al., 2022; Lozano‐Montoya et al., 2017). Furthermore, several evidence gaps within the reviews have also become apparent such as: effects of multi‐component interventions focused on older adults with multiple vulnerabilities, effects of community‐focused interventions adapted to local needs, or interventions that impacted client‐centered outcomes. Moreover, we did not identify reviews summarizing effects of interventions designed to mitigate the effects of poverty or focused on marginalized communities in LMICs.

The results of this umbrella review are consistent with the literature. An umbrella review of seven systematic reviews including 58 randomized trials found a benefit of resistance training and multicomponent exercise interventions that include resistance, aerobic, balance and flexibility tasks (Jadczak et al., 2018). An overview of 10 systematic reviews found a benefit of exercise combined with amino acid supplementation (Lozano‐Montoya et al., 2017). A recently published large‐scale longitudinal cohort study found that exercise therapy was associated with a greater likelihood of improvement in frailty status among older adults receiving home care services (Larsen et al., 2020). Despite its observational nature, this study involving 250,428 people from several jurisdictions provides real world evidence of potential benefit of community exercise programs.

The fact that physical activity may improve social functioning (social vulnerability) in addition to frailty (health vulnerability) is critical. Indeed, many older adults may fall into more than one vulnerability category (Johnson & Wiener, 2006; Lee et al., 2018). Given the interrelationship and potential negative interactions between health, social and financial vulnerability, the interventions that target only one vulnerability category may fail to address its underlying determinants and may not be effective in the long‐term. Therefore, physical activity could be an essential component of multimodal health promotion interventions that aim to address more than one vulnerability category. Nevertheless, more primary research is needed to identify other effective strategies to address more than one vulnerability category within the same study population.

Regarding technology interventions, a previous review of systematic reviews found that Internet‐supported interpersonal communication such as videoconferencing shown evidence of effectiveness in reducing loneliness (Chipps et al., 2017). This is in line with our results that suggest that Internet use may improve social isolation and loneliness. A recently published evidence and gap map documented the many intervention categories used in digital interventions (Campbell Collaboration; Welch et al., 2022). This review again documented the lack of equity considerations in this field of study.

This umbrella review revealed additional areas of knowledge gaps. First, published reviews did not identify many large‐scale community‐based real‐world interventions that engaged communities and adapted programs to local needs. One review (Hagan et al., 2014) reported a complex intervention, the “Community Connections,” a community‐based program designed to engage and mobilize resources for older adults in effective interactions with younger populations. However, the authors of the review commented that this primary study was done on a small scale and was not able to adequately evaluate the program's potential impact on loneliness. Second, there was no comment on the location of primary studies within individual reviews. Indeed, our scan of primary studies within reviews appeared to all be from middle‐ and high‐income countries and no studies were conducted in low‐income countries. We did identify one published trial of a peer‐to‐peer support pilot intervention to improve quality of life among highly vulnerable, low‐income older adults in Cape Town, South Africa. This study demonstrated an increase of social interactions and physical activities and a decrease of loneliness after volunteers visits with clients. This very recent primary study was not included in the reviews we selected but does provide evidence that these interventions are feasible in low and middle income settings (Geffen et al., 2019).

Due to methodological flaws, the overall quality of the reviews ranges from low to critically low. This is primarily due to the absence of prespecified protocol, the absence of a list of excluded studies with justification of their exclusion and the lack of satisfactory technique for assessing the risk of bias (RoB) of included observational studies. Although reporting guidelines and quality assessment tools exist, adherence to reporting guidelines and quality assessment tools are not consistent across systematic reviews (Moher et al., 2015; Pussegoda et al., 2017; Shamseer et al., 2015). Some evidence is emerging that biases within systematic reviews could influence results and quality of overviews of systematic reviews (Page et al., 2014). Therefore, mechanisms to improve adherence to established reporting guidelines and methodological assessment tools are needed to improve the quality of systematic reviews.

Regarding generalizability, for the majority of the reviews, populations included in the primary studies' suffered from several health and/or social issues such as prefrailty, frailty, receiving home care services, having disability, cancer, geriatric syndromes, chronic diseases, anxiety, depression, mental health conditions, social conditions (social isolation, loneliness), or diverse health conditions. However, participants' sociodemographic were not captured or reported within individual reviews with one exception. Only one review aimed to identify effective interventions to address social isolation and loneliness in community‐dwelling older adults of ethnic minority groups. This review found that volunteering activity, educational activity, physical activity and physical activity combined with educational activity may improve social isolation and loneliness. However, these results were based on a small number of studies and all the included studies were done in the United States (Pool et al., 2017). In addition, none of the included reviews addressed the impact of the interventions on gender. While there are a number of studies and reviews that focus on loneliness and/or social isolation among older adults in the general population as well as known male and female differences in loneliness, there appear to be few sex/gender sensitive interventions designed to target it (Maes et al., 2019; Rapid Response Service, 2020). Because of the victimization and discrimination toward them, many lesbians, gay, bisexual, and transgender, queer and 2‐spirit (LGBTQ2+) older adults are vulnerable to social isolation or loneliness. LGBTQ2 + older adults are more likely to live alone and less likely to have children as their non‐LGBTQ2+ peers. This further puts this population at a higher risk of loneliness and social isolation and requires specific attention (Disparities, 2011; Goldsen, 2018). Therefore, the results of this umbrella review could apply to community‐dwelling older adults presenting health and social conditions, but more research is needed to identify what strategies are effective among, financially vulnerable older adults, ethnic and gender minorities.

The strengths of this umbrella review include the comprehensiveness of the search strategy, the recency of the reviews and the large number of primary studies and participants representing the effects interventions on frailty, social isolation and loneliness in community‐dwelling older adults. It is also the first umbrella review that was fully designed to address more than one area of vulnerability. A limitation of this umbrella review is restriction of inclusion to English and French languages. The Four studies that were excluded might have contained relevant evidence. It is possible that there are other recent reviews published in other languages that were not captured (Naito et al., 2021). However, we believe it would be unlikely given that social isolation and loneliness are primarily observed in high‐income countries and manuscripts are usually published in English language. Another limitation is the lack of consensus in the definition of frailty and the use of various criteria to define frailty. Given the growing body of research in this topic there is an urgent need for standardizing the measurement of frailty using frailty assessment scales. Finally, the umbrella review did not capture reviews that identified large‐scale community‐based real‐world interventions with community engagement and adaptation of programs to local needs. This may be explained by the fact that we addressed vulnerabilities rather than healthy aging outcomes and we focused on peer‐reviewed publications.

### 5.1 Implications for practice

For decision makers, behavior change communication messages need to emphasize the importance of physical activity and nutritional supplementation. Given the interplay between loneliness, social isolation and frailty, and the possible role of social isolation and loneliness in the risk of sedentary behaviors (Hawkley et al., 2009; Newall et al., 2013; Schrempft et al., 2019) and malnutrition (Eskelinen et al., 2016; Ramic et al., 2011) among older adults, interventions addressing frailty should be combined with strategies targeting social isolation and loneliness. For healthcare professionals, our review suggests that recommendations of lifestyle interventions including diet and physical activity may be worthwhile. Such interventions may be implemented within rapidly expanding social prescribing programs, assuming systems are in place to support the delivery of social and health promotion activities. We did not seek out the term “social prescribing.” We also did not review core elements of such programs to ensure that they are successfully deliver on improved health and social outcomes.

### 5.2 Recommendations for future research

In this umbrella review, we identified several knowledge gaps. Specifically, studies aiming to mitigate multiple risk factors, community‐based interventions designed by and for older adults, studies in LMICs, interventions aiming to mitigate vulnerabilities in equity seeking groups including the LGBTQ2+ and ethnic minorities would also be very important. Furthermore, the Internet can be used in a variety of ways such as visiting social network websites, interpersonal communication, vlogs, sit‐down videos. Therefore, it is important that future research evaluating the impact of Internet use on social isolation and loneliness define what specific activity they focus on.

## 6 CONCLUSION

Overall, our umbrella review documented that under controlled conditions, interventions including diets, physical activity and technology may improve frailty, social isolation or loneliness. Even though reviews had some methodological limitations, most of them rated the quality of the primary studies as moderate to high. Diet and exercise programs are worth considering for implementation and support. Further research and evaluation should focus on real world program development with community engagement foci, consider effects on gender and ethnic minorities, longer term client or system‐based outcomes and seek to assess interventions in rural and remote areas as well as under‐resourced geographies.

## AUTHOR CONTRIBUTIONS

P. Léon, J. Jbilou, J. Kaczorowski, M. Girard and P. Hébert designed the Connection‐NB Project. R. Adekpedjou, P. Léon, J. Jbilou, J. Kaczorowski, M. Girard, and P. Hébert conceptualized and designed the review protocol. P. Léon, C. Sauvé, and P. Hébert developed the search strategy. R. Adekpedjou, P. Léon, Omar Dewidar, Ali‐Alzubaidi, and Tarannum Hussain conducted the study selection. R. Adekpedjou, Omar Dewidar, Ali‐Alzubaidi, M. Conde, C. L. Calderon Ramirez conducted the data extraction. Omar Dewidar, Ali‐Alzubaidi, M. Conde, and C. L. Calderon Ramirez performed the quality assessment. R. Adekpedjou and Omar Dewidar conducted the data synthesis which was revised by P. Hébert. All authors contributed to data interpretation, revised the manuscript critically for key intellectual content, and gave their consent on the version to be published.

## Supporting information

Supporting information.

Supporting information.

Supporting information.

Supporting information.

Supporting information.

Supporting information.

Supporting information.

Supporting information.

## References

[cl21323-bib-0001] Adepoju, O. E. , Chae, M. , Woodard, L. , Smith, K. L. , Herrera, L. , Han, D. , Howard, D. L. , Dobbins, J. , & Ory, M. (2021). Correlates of social isolation among Community‐Dwelling older adults during the COVID‐19 pandemic. Frontiers in Public Health, 9, 702965. 10.3389/fpubh.2021.702965 34956998 PMC8702646

[cl21323-bib-0002] Anton, S. D. , Hida, A. , Mankowski, R. , Layne, A. , Solberg, L. M. , Mainous, A. G. , & Buford, T. (2018). Nutrition and exercise in sarcopenia. Current Protein & Peptide Science, 19(7), 649–667. 10.2174/1389203717666161227144349 28029078

[cl21323-bib-0003] Arantes, P. M. M. , Alencar, M. A. , Dias, R. C. , Dias, J. M. D. , & Pereira, L. S. M. (2009). Physical therapy treatment on frailty syndrome: Systematic review. Brazilian Journal of Physical Therapy/Revista Brasileira de Fisioterapia, 13(5), 365–375.

[cl21323-bib-0004] Aromataris, E. , Fernandez, R. , Godfrey, C. M. , Holly, C. , Khalil, H. , & Tungpunkom, P. (2015). Summarizing systematic reviews: Methodological development, conduct and reporting of an umbrella review approach. International Journal of Evidence‐Based Healthcare, 13(3), 132–140. 26360830 10.1097/XEB.0000000000000055

[cl21323-bib-0005] Brady, S. , D'Ambrosio, L. A. , Felts, A. , Rula, E. Y. , Kell, K. P. , & Coughlin, J. F. (2020). Reducing isolation and loneliness through membership in a fitness program for older adults: Implications for health. Journal of Applied Gerontology, 39(3), 301–310. 10.1177/0733464818807820 30392420 PMC7005930

[cl21323-bib-0006] Burton, E. , Farrier, K. , Galvin, R. , Johnson, S. , Horgan, N. F. , Warters, A. , & HIll, K. D. (2019). Physical activity programs for older people in the community receiving home care services: Systematic review and meta‐analysis. Clinical Interventions in Aging, 14, 1045–1064. 10.2147/CIA.S205019 31239654 PMC6559239

[cl21323-bib-0007] Cattan, M. , White, M. , Bond, J. , & Learmouth, A. (2005). Preventing social isolation and loneliness among older people: A systematic review of health promotion interventions. Ageing and Society, 25(1), 41–67. 10.1017/S0144686X04002594 27736564

[cl21323-bib-0008] Chen, Y.‐R. R. , & Schulz, P. J. (2016). The effect of information communication technology interventions on reducing social isolation in the elderly: A systematic review. Journal of Medical Internet Research, 18(1), e18. 26822073 10.2196/jmir.4596 PMC4751336

[cl21323-bib-0009] Chipps, J. , Jarvis, M. A. , & Ramlall, S. (2017). The effectiveness of e‐Interventions on reducing social isolation in older persons: A systematic review of systematic reviews. Journal of Telemedicine and Telecare, 23(10), 817–827. 28958209 10.1177/1357633X17733773

[cl21323-bib-0010] Cohen‐Mansfield, J. , & Perach, R. (2015). Interventions for alleviating loneliness among older persons: A critical review. American Journal of Health Promotion, 29(3), e109–e125. 10.4278/ajhp.130418-LIT-182 24575725

[cl21323-bib-0011] Campbell Collaboration . (2022). *Digital interventions to reduce social isolation and loneliness in older adults*. World Health Organization. Retrieved January 30, 2023, from https://www.who.int/initiatives/decade-of-healthy-ageing/evidence-gap-map/sil-digital

[cl21323-bib-0012] Coll‐Planas, L. , Nyqvist, F. , Puig, T. , Urrutia, G. , Sola, I. , & Monteserin, R. (2017). Social capital interventions targeting older people and their impact on health: A systematic review. Journal of Epidemiology & Community Health, 71(7), 663–672. 10.1136/jech-2016-208131 27834223

[cl21323-bib-0013] Conneely, M. , Leahy, S. , Dore, L. , Trépel, D. , Robinson, K. , Jordan, F. , & Galvin, R. (2022). The effectiveness of interventions to reduce adverse outcomes among older adults following Emergency Department discharge: Umbrella review. BMC Geriatrics, 22(1), 462. 10.1186/s12877-022-03007-5 35643453 PMC9145107

[cl21323-bib-0014] Dedeyne, L. , Deschodt, M. , Verschueren, S. , Tournoy, J. , & Gielen, E. (2017). Effects of multi‐domain interventions in (pre)frail elderly on frailty, functional, and cognitive status: A systematic review. Clinical Interventions in Aging, 12, 873–896. 10.2147/CIA.S130794 28579766 PMC5448695

[cl21323-bib-0015] Department of Economic and Social Affairs programme on ageing . (2020). The focal point on ageing in the United Nations system. *Income poverty in old age: An emerging development priority*. https://www.un.org/esa/socdev/ageing/documents/PovertyIssuePaperAgeing.pdf

[cl21323-bib-0016] Di Lorito, C. , Long, A. , Byrne, A. , Harwood, R. H. , Gladman, J. R. F. , Schneider, S. , Logan, P. , Bosco, A. , & van der Wardt, V. (2021). Exercise interventions for older adults: A systematic review of meta‐analyses. Journal of Sport and Health Science, 10(1), 29–47. 10.1016/j.jshs.2020.06.003 32525097 PMC7858023

[cl21323-bib-0017] Dickens, A. P. , Richards, S. H. , Greaves, C. J. , & Campbell, J. L. (2011). Interventions targeting social isolation in older people: A systematic review. BMC Public Health, 11(1), 647. 10.1186/1471-2458-11-647 21843337 PMC3170621

[cl21323-bib-0018] Disparities, P. (2011). The diverse elders coalition and LGBT aging: Connecting communities, issues, and resources in a historic moment. Public Policy & Aging Report, 8.

[cl21323-bib-0019] Ekwall, A. K. , Sivberg, B. , & Hallberg, I. R. (2005). Loneliness as a predictor of quality of life among older caregivers. Journal of Advanced Nursing, 49(1), 23–32. 10.1111/j.1365-2648.2004.03260.x 15610378

[cl21323-bib-0020] Eskelinen, K. , Hartikainen, S. , & Nykänen, I. (2016). Is loneliness associated with malnutrition in older people? International Journal of Gerontology, 10(1), 43–45.

[cl21323-bib-0021] Fancourt, D. , & Steptoe, A. (2018). Community group membership and multidimensional subjective well‐being in older age. Journal of Epidemiology and Community Health, 72(5), 376–382. 10.1136/jech-2017-210260 29440307 PMC5909739

[cl21323-bib-0022] Franck, L. , Molyneux, N. , & Parkinson, L. (2016). Systematic review of interventions addressing social isolation and depression in aged care clients. Quality of Life Research, 25(6), 1395–1407. 10.1007/s11136-015-1197-y 26646806

[cl21323-bib-0023] Fried, L. P. , Tangen, C. M. , Walston, J. , Newman, A. B. , Hirsch, C. , Gottdiener, J. , Seeman, T. , Tracy, R. , Kop, W. J. , Burke, G. , & McBurnie, M. A. (2001). Frailty in older adults: Evidence for a phenotype. The Journals of Gerontology Series A: Biological Sciences and Medical Sciences, 56(3), M146–M157. 11253156 10.1093/gerona/56.3.m146

[cl21323-bib-0024] Frost, R. , Belk, C. , Jovicic, A. , Ricciardi, F. , Kharicha, K. , Gardner, B. , Iliffe, S. , Goodman, C. , Manthorpe, J. , Drennan, V. M. , & Walters, K. (2017). Health promotion interventions for community‐dwelling older people with mild or pre‐frailty: A systematic review and meta‐analysis. BMC Geriatrics, 17(1), 157. 10.1186/s12877-017-0547-8 28728570 PMC5520298

[cl21323-bib-0025] Fu, Z. , Yan, M. , & Meng, C. (2022). The effectiveness of remote delivered intervention for loneliness reduction in older adults: A systematic review and meta‐analysis. Frontiers in Psychology, 13, 935544. 10.3389/fpsyg.2022.935544 35967719 PMC9372715

[cl21323-bib-0026] Gale, C. R. , Westbury, L. , & Cooper, C. (2018). Social isolation and loneliness as risk factors for the progression of frailty: The English Longitudinal Study of Ageing. Age and Ageing, 47(3), 392–397. 29309502 10.1093/ageing/afx188 PMC5920346

[cl21323-bib-0027] Geffen, L. N. , Kelly, G. , Morris, J. N. , & Howard, E. P. (2019). Peer‐to‐peer support model to improve quality of life among highly vulnerable, low‐income older adults in Cape Town, South Africa. BMC Geriatrics, 19(1), 279. 31640576 10.1186/s12877-019-1310-0 PMC6805367

[cl21323-bib-0028] Goldsen, K. F. (2018). Shifting social context in the lives of LGBTQ older adults. The Public Policy and Aging Report, 28(1), 24–28. 29881245 10.1093/ppar/pry003 PMC5972452

[cl21323-bib-0029] Hagan, R. , Manktelow, R. , Taylor, B. J. , & Mallett, J. (2014). Reducing loneliness amongst older people: A systematic search and narrative review. Aging & Mental Health, 18(6), 683–693. 10.1080/13607863.2013.875122 24437736

[cl21323-bib-0030] Hawkley, L. C. , Thisted, R. A. , & Cacioppo, J. T. (2009). Loneliness predicts reduced physical activity: Cross‐sectional & longitudinal analyses. Health Psychology, 28(3), 354–363. 19450042 10.1037/a0014400 PMC2791498

[cl21323-bib-0031] Hayajneh, A. A. , & Rababa, M. (2021). The association of frailty with poverty in older adults: A systematic review. Dementia and Geriatric Cognitive Disorders, 5, 407–413. 10.1159/000520486 34929708

[cl21323-bib-0032] Heins, P. , Boots, L. M. M. , Koh, W. Q. , Neven, A. , Verhey, F. R. J. , & de Vugt, M. E. (2021). The effects of technological interventions on social participation of community‐dwelling older adults with and without dementia: A systematic review. Journal of Clinical Medicine, 10(11), 2308. 10.3390/jcm10112308 34070660 PMC8198527

[cl21323-bib-0033] Hoogendijk, E. O. , Afilalo, J. , Ensrud, K. E. , Kowal, P. , Onder, G. , & Fried, L. P. (2019). Frailty: Implications for clinical practice and public health. The Lancet, 394(10206), 1365–1375. 10.1016/S0140-6736(19)31786-6 31609228

[cl21323-bib-0034] Ibrahim, A. F. , Tan, M. P. , Teoh, G. K. , Muda, S. M. , & Chong, M. C. (2022). Health benefits of social participation interventions among community‐dwelling older persons: A review article. Experimental Aging Research, 48(3), 234–260. 10.1080/0361073x.2021.1939563 34229584

[cl21323-bib-0035] Jadczak, A. D. , Makwana, N. , Luscombe‐Marsh, N. , Visvanathan, R. , & Schultz, T. J. (2018). Effectiveness of exercise interventions on physical function in community‐dwelling frail older people: an umbrella review of systematic reviews. JBI Database of Systematic Reviews and Implementation Reports, 16(3), 752–775. 29521871 10.11124/JBISRIR-2017-003551

[cl21323-bib-0036] Johnson, R. W. , & Wiener, J. M. (2006). *A profile of frail older Americans and their caregivers*. Urban Institute.

[cl21323-bib-0037] Kaplan, D. , & Berkman, B. (2019). Older adults living alone. Merck Manual, Professional Version Merck & Co, Inc.

[cl21323-bib-0038] Kelaiditi, E. , van Kan, G. A. , & Cesari, M. (2014). Frailty: Role of nutrition and exercise. Current Opinion in Clinical Nutrition & Metabolic Care, 17(1), 32–39. 10.1097/MCO.0000000000000008 24281373

[cl21323-bib-0039] Khosravi, P. , Rezvani, A. , & Wiewiora, A. (2016). The impact of technology on older adults' social isolation. Computers in Human Behavior, 63, 594–603. 10.1016/j.chb.2016.05.092

[cl21323-bib-0040] Krnic Martinic, M. , Pieper, D. , Glatt, A. , & Puljak, L. (2019). Definition of a systematic review used in overviews of systematic reviews, meta‐epidemiological studies and textbooks. BMC Medical Research Methodology, 19(1), 203. 10.1186/s12874-019-0855-0 31684874 PMC6829801

[cl21323-bib-0041] Larsen, R. T. , Turcotte, L. A. , Westendorp, R. , Langberg, H. , & Hirdes, J. P. (2020). Frailty Index status of Canadian home care clients improves with exercise therapy and declines in the presence of polypharmacy. Journal of the American Medical Directors Association, 21(6), 766–771. 32165063 10.1016/j.jamda.2020.01.004

[cl21323-bib-0042] Lee, D. R. , Santo, E. C. , Lo, J. C. , Ritterman Weintraub, M. L. , Patton, M. , & Gordon, N. P. (2018). Understanding functional and social risk characteristics of frail older adults: A cross‐sectional survey study. BMC Family Practice, 19(1), 170. 30340530 10.1186/s12875-018-0851-1 PMC6195739

[cl21323-bib-0043] Li, J. , Zhou, X. , & Wang, Q. (2022). Interventions to reduce loneliness among Chinese older adults: A network meta‐analysis of randomized controlled trials and quasi‐experimental studies. Applied Psychology: Health and Well‐Being, 15, 238–258. 10.1111/aphw.12375 35621111

[cl21323-bib-0044] Liao, C. D. , Lee, P. H. , Hsiao, D. J. , Huang, S. W. , Tsauo, J. Y. , Chen, H. C. , & Liou, T. H. (2018). Effects of protein supplementation combined with exercise intervention on frailty indices, body composition, and physical function in frail older adults. Nutrients, 10(12), 1916. 10.3390/nu10121916 30518122 PMC6315527

[cl21323-bib-0045] Looman, W. M. , Huijsman, R. , & Fabbricotti, I. N. (2019). The (cost‐)effectiveness of preventive, integrated care for community‐dwelling frail older people: A systematic review. Health & Social Care in the Community, 27(1), 1–30. 10.1111/hsc.12571 29667259 PMC7379491

[cl21323-bib-0046] Lozano‐Montoya, I. , Correa‐Pérez, A. , Abraha, I. , et al. (2017). Nonpharmacological interventions to treat physical frailty and sarcopenia in older patients: a systematic overview–the SENATOR Project ONTOP Series. Clinical Interventions in Aging, 12, 721. 28490866 10.2147/CIA.S132496 PMC5413484

[cl21323-bib-0047] Luanaigh, C. Ó. , & Lawlor, B. A. (2008). Loneliness and the health of older people. International Journal of Geriatric Psychiatry, 23(12), 1213–1221. 18537197 10.1002/gps.2054

[cl21323-bib-0048] Maes, M. , Qualter, P. , Vanhalst, J. , Van den Noortgate, W. , & Goossens, L. (2019). Gender differences in loneliness across the lifespan: A meta–analysis. European Journal of Personality, 33(6), 642–654.

[cl21323-bib-0049] Makizako, H. , Shimada, H. , Doi, T. , Tsutsumimoto, K. , Hotta, R. , Nakakubo, S. , Makino, K. , & Lee, S. (2018). Social frailty leads to the development of physical frailty among physically non‐frail adults: A four‐year follow‐up longitudinal cohort study. International Journal of Environmental Research and Public Health, 15(3), 490. 29534470 10.3390/ijerph15030490 PMC5877035

[cl21323-bib-0050] Mehrabi, F. , & Béland, F. (2020). Effects of social isolation, loneliness and frailty on health outcomes and their possible mediators and moderators in community‐dwelling older adults: A scoping review. Archives of Gerontology and Geriatrics, 90, 104119. 32562956 10.1016/j.archger.2020.104119

[cl21323-bib-0051] Moher, D. , Shamseer, L. , Clarke, M. , Ghersi, D. , Liberati, A. , Petticrew, M. , Shekelle, P. , & Stewart, L. A. (2015). Preferred reporting items for systematic review and meta‐analysis protocols (PRISMA‐P) 2015 statement. Systematic Reviews, 4(1), 1. 25554246 10.1186/2046-4053-4-1 PMC4320440

[cl21323-bib-0052] Naito, R. , Leong, D. P. , Bangdiwala, S. I. , McKee, M. , Subramanian, S. V. , Rangarajan, S. , Islam, S. , Avezum, A. , Yeates, K. E. , Lear, S. A. , Gupta, R. , Yusufali, A. , Dans, A. L. , Szuba, A. , Alhabib, K. F. , Kaur, M. , Rahman, O. , Seron, P. , Diaz, R. , … Yusuf, S. (2021). Impact of social isolation on mortality and morbidity in 20 high‐income, middle‐income and low‐income countries in five continents. BMJ Global Health, 6(3), e004124. 10.1136/bmjgh-2020-004124 33753400 PMC7986654

[cl21323-bib-0053] Newall, N. E. G. , Chipperfield, J. G. , Bailis, D. S. , & Stewart, T. L. (2013). Consequences of loneliness on physical activity and mortality in older adults and the power of positive emotions. Health Psychology, 32(8), 921–924. 22888819 10.1037/a0029413

[cl21323-bib-0054] Noice, T. , Noice, H. , & Kramer, A. F. (2014). Participatory arts for older adults: A review of benefits and challenges. The Gerontologist, 54(5), 741–753. 10.1093/geront/gnt138 24336875 PMC4229893

[cl21323-bib-0055] Page, M. J. , McKenzie, J. E. , Kirkham, J. , Dwan, K. , Kramer, S. , Green, S. , & Forbes, A. (2014). Bias due to selective inclusion and reporting of outcomes and analyses in systematic reviews of randomised trials of healthcare interventions. Cochrane Database of Systematic Reviews, 2014(10), MR000035. 25271098 10.1002/14651858.MR000035.pub2 PMC8191366

[cl21323-bib-0056] Pool, M. S. , Agyemang, C. O. , & Smalbrugge, M. (2017). Interventions to improve social determinants of health among elderly ethnic minority groups: A review. European Journal of Public Health, 27(6), 1048–1054. 10.1093/eurpub/ckx178 29095995

[cl21323-bib-0057] Prohaska, T. , Burholt, V. , Burns, A. , Golden, J. , Hawkley, L. , Lawlor, B. , Leavey, G. , Lubben, J. , O'Sullivan, R. , Perissinotto, C. , van Tilburg, T. , Tully, M. , Victor, C. , & Fried, L. (2020). Consensus statement: Loneliness in older adults, the 21st century social determinant of health? BMJ Open, 10(8), e034967. 10.1136/bmjopen-2019-034967 PMC7422633 32788184

[cl21323-bib-0058] Pussegoda, K. , Turner, L. , Garritty, C. , Mayhew, A. , Skidmore, B. , Stevens, A. , Boutron, I. , Sarkis‐Onofre, R. , Bjerre, L. M. , Hróbjartsson, A. , Altman, D. G. , & Moher, D. (2017). Systematic review adherence to methodological or reporting quality. Systematic Reviews, 6(1), 131. 28720117 10.1186/s13643-017-0527-2 PMC5516390

[cl21323-bib-0059] Puts, M. T. E. , Toubasi, S. , Andrew, M. K. , Ashe, M. C. , Ploeg, J. , Atkinson, E. , Ayala, A. P. , Roy, A. , Rodríguez Monforte, M. , Bergman, H. , & McGilton, K. (2017). Interventions to prevent or reduce the level of frailty in community‐dwelling older adults: A scoping review of the literature and international policies. Age and Ageing, 46(3), 383–392. 10.1093/ageing/afw247 28064173 PMC5405756

[cl21323-bib-0060] Ramic, E. , Pranjic, N. , Batic‐Mujanovic, O. , Karic, E. , Alibasic, E. , & Alic, A. (2011). The effect of loneliness on malnutrition in elderly population. Medicinski Arhiv, 65(2), 92–95. 21585182

[cl21323-bib-0061] Rapid Response Service . (2020). Interventions to reduce social isolation and loneliness among men who have sex with men. Ontario HIV Treatment Network.

[cl21323-bib-0062] Rockwood, K. , & Mitnitski, A. (2007). Frailty in relation to the accumulation of deficits. The Journals of Gerontology Series A: Biological Sciences and Medical Sciences, 62(7), 722–727. 10.1093/gerona/62.7.722 17634318

[cl21323-bib-0063] Schrempft, S. , Jackowska, M. , Hamer, M. , & Steptoe, A. (2019). Associations between social isolation, loneliness, and objective physical activity in older men and women. BMC Public Health, 19(1), 74. 30651092 10.1186/s12889-019-6424-y PMC6335852

[cl21323-bib-0064] Sepúlveda‐Loyola, W. , Rodríguez‐Sánchez, I. , Pérez‐Rodríguez, P. , Ganz, F. , Torralba, R. , Oliveira, D. V. , & Rodríguez‐Mañas, L. (2020). Impact of social isolation due to COVID‐19 on health in older people: Mental and physical effects and recommendations. The Journal of Nutrition, Health & Aging, 24(9), 938–947. 10.1007/s12603-020-1469-2 PMC7597423 33155618

[cl21323-bib-0065] Shamseer, L. , Moher, D. , Clarke, M. , Ghersi, D. , Liberati, A. , Petticrew, M. , Shekelle, P. , & Stewart, L. A. (2015). Preferred reporting items for systematic review and meta‐analysis protocols (PRISMA‐P) 2015: Elaboration and explanation. BMJ, 349, g7647. 10.1136/bmj.g7647 25555855

[cl21323-bib-0066] Shea, B. J. , Reeves, B. C. , Wells, G. , Thuku, M. , Hamel, C. , Moran, J. , Moher, D. , Tugwell, P. , Welch, V. , Kristjansson, E. , & Henry, D. A. (2017). AMSTAR 2: A critical appraisal tool for systematic reviews that include randomised or non‐randomised studies of healthcare interventions, or both. BMJ, 358, j4008. 28935701 10.1136/bmj.j4008 PMC5833365

[cl21323-bib-0067] Shvedko, A. , Whittaker, A. C. , Thompson, J. L. , & Greig, C. A. (2018). Physical activity interventions for treatment of social isolation, loneliness or low social support in older adults: A systematic review and meta‐analysis of randomised controlled trials. Literature Review; Systematic Review; Meta Analysis. Psychology of Sport and Exercise, 34, 128–137. 10.1016/j.psychsport.2017.10.003

[cl21323-bib-0068] Sims‐Gould, J. , Tong, C. E. , Wallis‐Mayer, L. , & Ashe, M. C. (2017). Reablement, reactivation, rehabilitation and restorative interventions with older adults in receipt of home care: A systematic review. Journal of the American Medical Directors Association, 18(8), 653–663. 10.1016/j.jamda.2016.12.070 28214234

[cl21323-bib-0069] Smith, T. O. , Jimoh, O. F. , Cross, J. , Allan, L. , Corbett, A. , Sadler, E. , Khondoker, M. , Whitty, J. , Valderas, J. M. , & Fox, C. (2019). Social prescribing programmes to prevent or delay frailty in community‐dwelling older adults. Geriatrics, 4(4):65. 10.3390/geriatrics4040065 31783654 PMC6960851

[cl21323-bib-0070] Snowden, M. B. , Steinman, L. E. , Carlson, W. L. , Mochan, K. N. , Abraido‐Lanza, A. F. , Bryant, L. L. , Duffy, M. , Knight, B. G. , Jeste, D. V. , Leith, K. H. , Lenze, E. J. , Logsdon, R. G. , Satariano, W. A. , Zweiback, D. J. , & Anderson, L. A. (2014). Effect of physical activity, social support, and skills training on late‐life emotional health: A systematic literature review and implications for public health research. Frontiers in Public Health, 2, 213. 10.3389/fpubh.2014.00213 25964921 PMC4410348

[cl21323-bib-0071] Stolz, E. , Mayerl, H. , Waxenegger, A. , & Freidl, W. (2017). Explaining the impact of poverty on old‐age frailty in Europe: Material, psychosocial and behavioural factors. European Journal of Public Health, 27(6), 1003–1009. 29020312 10.1093/eurpub/ckx079 PMC5881693

[cl21323-bib-0072] Theou, O. , Stathokostas, L. , Roland, K. P. , Jakobi, J. M. , Patterson, C. , Vandervoort, A. A. , & Jones, G. R. (2011). The effectiveness of exercise interventions for the management of frailty: A systematic review. Journal of Aging Research, 2011, 569194. 10.4061/2011/569194 21584244 PMC3092602

[cl21323-bib-0073] Thomson, L. J. , Lockyer, B. , Camic, P. M. , & Chatterjee, H. J. (2018). Effects of a museum‐based social prescription intervention on quantitative measures of psychological wellbeing in older adults. Perspectives in Public Health, 138(1), 28–38. 10.1177/1757913917737563 29130869

[cl21323-bib-0074] Tricco, A. C. , Thomas, S. M. , Radhakrishnan, A. , Ramkissoon, N. , Mitchell, G. , Fortune, J. , Jiang, Y. , de Groh, M. , Anderson, K. , Barker, J. , Gauthier‐Beaupré, A. , Watt, J. , & Straus, S. E. (2022). Interventions for social isolation in older adults who have experienced a fall: A systematic review. BMJ Open, 12(3), e056540. 10.1136/bmjopen-2021-056540 PMC8915346 35264363

[cl21323-bib-0075] Waddell, K. , Panchal, P. , & Wilson, M. G. (2018). Rapid synthesis: Identifying indicators and rates of poverty among older adults. McMaster Health Forum.

[cl21323-bib-0076] Walters, K. , Frost, R. , Kharicha, K. , Avgerinou, C. , Gardner, B. , Ricciardi, F. , Hunter, R. , Liljas, A. , Manthorpe, J. , Drennan, V. , Wood, J. , Goodman, C. , Jovicic, A. , & Iliffe, S. (2017). Home‐based health promotion for older people with mild frailty: The HomeHealth intervention development and feasibility RCT. Health Technology Assessment, 21(73), 1–128. 10.3310/hta21730 PMC5742456 29214975

[cl21323-bib-0077] Watts, P. N. , Blane, D. , & Netuveli, G. (2019). Minimum income for healthy living and frailty in adults over 65 years old in the English Longitudinal Study of Ageing: A population‐based cohort study. BMJ Open, 9(2), e025334. 10.1136/bmjopen-2018-025334 PMC6398705 30819709

[cl21323-bib-0078] Welch, V. , Ghogomu, E. T. , Barbeau, V. I. , Boulton, E. , Boutin, S. , Haitas, N. , Kneale, D. , Salzwedel, D. M. , Simard, R. , Herbert, P. , & Mikton, C. (2022). PROTOCOL: Digital interventions to reduce social isolation and loneliness in older adults: An evidence and gap map. Campbell Systematic Reviews, 18(3), e1260. 10.1002/cl2.1260 36909878 PMC9233308

[cl21323-bib-0079] WHO . *Ageing and health*. Retrieved January 5, 2022, from https://www.who.int/news-room/fact-sheets/detail/ageing-and-health#:~:text=Common%20health%20conditions%20associated%20with,%2C%20diabetes%2C%20depression%20and%20dementia

[cl21323-bib-0080] World Health Organization . (2023). Social isolation and loneliness among older people: Advocacy brief. https://www.who.int/publications/i/item/9789240030749

[cl21323-bib-0081] World Health Organization . *Decade of healthy ageing baseline report*. https://www.who.int/publications/i/item/9789240017900

[cl21323-bib-0082] Wister, A. , Fyffe, I. , & O'Dea, E. (2021). Technological interventions for loneliness and social isolation among older adults: A scoping review protocol. Systematic Reviews, 10(1), 217. 10.1186/s13643-021-01775-6 34362447 PMC8346339

[cl21323-bib-0083] Yanguas, J. , Pinazo‐Henandis, S. , & Tarazona‐Santabalbina, F. J. (2018). The complexity of loneliness. Acta bio‐medica: Atenei Parmensis, 89(2), 302–314. 10.23750/abm.v89i2.7404 29957768 PMC6179015

[cl21323-bib-0084] Youn, H. M. , Lee, H. J. , Lee, D. W. , & Park, E.‐C. (2020). The impact of poverty transitions on frailty among older adults in South Korea: Findings from the Korean longitudinal study of ageing. BMC Geriatrics, 20, 139. 32293296 10.1186/s12877-020-01522-x PMC7161157

