# Supplementary material for: Effectiveness of interventions to address different types of vulnerabilities in community‐dwelling older adults: An umbrella review
Source: Campbell Syst Rev. 2023 May 9;19(2):e1323. doi: 10.1002/cl2.1323 (PMC10168691; doi:10.1002/cl2.1323)
Supplement: Supplementary file 1 — Supporting information. [file CL2-19-e1323-s003.docx]

**Search strategy**

## Pubmed

"Community Integration"[Mesh] OR "Community Health Planning"[Mesh] OR "Therapeutic Community"[Mesh] OR "Community Psychiatry"[Mesh:NoExp] OR "Community Mental Health Services"[Mesh] OR "Community Medicine"[Mesh] OR "Community Health Services"[Mesh:NoExp] OR "Community Health Nursing"[Mesh] OR "Home Health Nursing"[Mesh] OR "Parish Nursing"[Mesh] OR "Community Pharmacy Services"[Mesh] OR "Home Care Services"[Mesh] OR community integration[tiab] OR community integration[ot] OR community navigation[tiab] OR community navigation[ot] OR community outreach[tiab] OR community outreach[ot] OR community health[tiab] OR community health[ot OR home care[tiab] OR home care[ot] OR intervention*[tiab] OR intervention*[ot] OR "Primary Health Care"[Mesh] OR integrated primary care[tiab] OR integrated primary care[ot] OR integrated care[tiab] OR integrated care[ot] OR intervention* OR citizen assistance[tiab] OR citizen assistance[ot] OR befriending[tiab] OR befriending[ot] OR assistance[tiab] OR assistance[ot] OR public health[tiab] OR public health[ot]

AND

poverty[tiab] OR poverty[ot] OR lonel*[tiab] OR lonel*[ot] OR "Frailty"[Mesh] OR frail*[tiab] OR frail*[ot] OR "Frailty"[Mesh] OR frail*[tiab] OR frail*[ot] OR "Loneliness"[Mesh] OR "Poverty"[Mesh:NoExp] OR "Social Isolation"[Mesh] OR "Social Alienation"[Mesh] OR social isolation[tiab] OR social isolation[ot] OR social alienation[tiab] OR social alienation[ot]

AND

older people[tiab] OR older people[ot] OR older adult*[tiab] OR older adult*[ot] OR "Aged"[Mesh] OR "Frail Elderly"[Mesh] OR "Aged, 80 AND over"[Mesh] OR aged person*[tiab] OR aged people*[tiab] OR elder[tiab] OR elders[tiab] OR "late life"[tiab] OR "80 AND over"[tiab] OR "65 year*"[tiab] OR "66 year*"[tiab] OR "67 year*"[tiab] OR "68 year*"[tiab] OR "69 year*"[tiab] OR "70 year*"[tiab] OR "71 year*"[tiab] OR "72 year*"[tiab] OR "73 year*"[tiab] OR "74 year*"[tiab] OR "75 year*"[tiab] OR "76 year*"[tiab] OR "77 year*"[tiab] OR "78 year*"[tiab] OR "79 year*"[tiab] OR "80 year*"[tiab] OR "81 year* "[tiab] OR "82 year*"[tiab] OR "83 year*"[tiab] OR "84 year*"[tiab] OR "85 year*"[tiab] OR "86 year*"[tiab] OR "87 year*"[tiab] OR "88 year*"[tiab] OR "89 year*"[tiab] OR "90 year*"[tiab] OR "91 year*"[tiab] OR "92 year*"[tiab] OR "93 year*"[tiab] OR "94 year*"[tiab] OR "95 year*"[tiab] OR "96 year*"[tiab] OR "97 year*"[tiab] OR "98 year*"[tiab] OR "99 year*"[tiab] OR "100 year*"[tiab] OR "101 year*"[tiab] OR "102 year*"[tiab] OR "103 year*"[tiab] OR "104 year*"[tiab] OR "105 year*"[tiab] OR "106 year*"[tiab] OR "107 year*"[tiab] OR "108 year*"[tiab] OR "109 year*"[tiab] OR "110 year*"[tiab] OR aged person*[ot] OR aged people*[ot] OR elder[ot] OR elders[ot] OR "late life"[ot] OR “80 AND over”[ot] OR "65 year* "[ot] OR "66 year* "[ot] OR "67 year*"[ot] OR "68 year*"[ot] OR "69 year*"[ot] OR "70 year*"[ot] OR "71 year*"[ot] OR "72 year*"[ot] OR "73 year*"[ot] OR "74 year*"[ot] OR "75 year*"[ot] OR "76 year*"[ot] OR "77 year*"[ot] OR "78 year*"[ot] OR "79 year*"[ot] OR "80 year*"[ot] OR "81 year*"[ot] OR "82 year*"[ot] OR "83 year*"[ot] OR "84 year*"[ot] OR "85 year*"[ot] OR "86 year*"[ot] OR "87 year*"[ot] OR "88 year*"[ot] OR "89 year*"[ot] OR "90 year*"[ot] OR "91 year*"[ot] OR "92 year*"[ot] OR "93 year*"[ot] OR "94 year*"[ot] OR "95 year*"[ot] OR "96 year*"[ot] OR "97 year*"[ot] OR "98 year*"[ot] OR "99 year*"[ot] OR "100 year*"[ot] OR "101 year*"[ot] OR "102 year*"[ot] OR "103 year*"[ot] OR "104 year*"[ot] OR "105 year*"[ot] OR "106 year*"[ot] OR "107 year*"[ot] OR "108 year*"[ot] OR "109 year*"[ot] OR "110 year*"[ot]

AND

systematic[sb] OR "Review" [Publication Type] OR systematic review*[tiab] OR systematic review*[ot] OR rapid review*[tiab] OR rapid review*[ot] OR scoping review*[tiab] OR scoping review*[ot] OR review*[tiab] AND "2009"[Date - Publication] : "2022"[Date - Publication]

**Résultats: 2281**

## MEDLINE

1. older people.ti,ab. or older people.kw. or older adult*.ti,ab. or older adult*.kw. or exp Aged/ or exp Frail Elderly/ or "Aged, 80 and over"/ or aged person*.ti,ab. or aged people*.ti,ab. or elder.ti,ab. or elders.ti,ab. or late life.ti,ab. or "80 and over".ti,ab. or 65 year*.ti,ab. or 66 year*.ti,ab. or 67 year*.ti,ab. or 68 year*.ti,ab. or 69 year*.ti,ab. or 70 year*.ti,ab. or 71 year*.ti,ab. or 72 year*.ti,ab. or 73 year*.ti,ab. or 74 year*.ti,ab. or 75 year*.ti,ab. or 76 year*.ti,ab. or 77 year*.ti,ab. or 78 year*.ti,ab. or 79 year*.ti,ab. or 80 year*.ti,ab. or 81 year*.ti,ab. or 82 year*.ti,ab. or 83 year*.ti,ab. or 84 year*.ti,ab. or 85 year*.ti,ab. or 86 year*.ti,ab. or 87 year*.ti,ab. or 88 year*.ti,ab. or 89 year*.ti,ab. or 90 year*.ti,ab. or 91 year*.ti,ab. or 92 year*.ti,ab. or 93 year*.ti,ab. or 94 year*.ti,ab. or 95 year*.ti,ab. or 96 year*.ti,ab. or 97 year*.ti,ab. or 98 year*.ti,ab. or 99 year*.ti,ab. or 100 year*.ti,ab. or 101 year*.ti,ab. or 102 year*.ti,ab. or 103 year*.ti,ab. or 104 year*.ti,ab. or 105 year*.ti,ab. or 106 year*.ti,ab. or 107 year*.ti,ab. or 108 year*.ti,ab. or 109 year*.ti,ab. or 110 year*.ti,ab. or aged person*.kw. or aged people*.kw. or elder.kw. or elders.kw. or late life.kw. or "80 and over".kw. or 65 year*.kw. or 66 year*.kw. or 67 year*.kw. or 68 year*.kw. or 69 year*.kw. or 70 year*.kw. or 71 year*.kw. or 72 year*.kw. or 73 year*.kw. or 74 year*.kw. or 75 year*.kw. or 76 year*.kw. or 77 year*.kw. or 78 year*.kw. or 79 year*.kw. or 80 year*.kw. or 81 year*.kw. or 82 year*.kw. or 83 year*.kw. or 84 year*.kw. or 85 year*.kw. or 86 year*.kw. or 87 year*.kw. or 88 year*.kw. or 89 year*.kw. or 90 year*.kw. or 91 year*.kw. or 92 year*.kw. or 93 year*.kw. or 94 year*.kw. or 95 year*.kw. or 96 year*.kw. or 97 year*.kw. or 98 year*.kw. or 99 year*.kw. or 100 year*.kw. or 101 year*.kw. or 102 year*.kw. or 103 year*.kw. or 104 year*.kw. or 105 year*.kw. or 106 year*.kw. or 107 year*.kw. or 108 year*.kw. or 109 year*.kw. or 110 year*.kw.
2. poverty.ti,ab. or poverty.kw. or lonel*.ti,ab. or lonel*.kw. or exp Frailty/ or frail*.ti,ab. or frail*.kw. or exp Frailty/ or frail*.ti,ab. or frail*.kw. or exp Loneliness/ or Poverty/ or exp Social Isolation/ or exp Social Alienation/ or social isolation.ti,ab. or social isolation.kw. or social alienation.ti,ab. or social alienation.kw. or functional impairment.ti,ab. or functional impairment.kw. or prefrail*.ti,ab. or prefrail*.kw.
3. exp Community Integration/ or exp Community Health Planning/ or exp Therapeutic Community/ or Community Psychiatry/ or exp Community Mental Health Services/ or exp Community Medicine/ or Community Health Services/ or exp Community Health Nursing/ or exp Home Health Nursing/ or exp Parish Nursing/ or exp Community Pharmacy Services/ or exp Home Care Services/ or community integration.ti,ab. or community integration.kw. or community navigation.ti,ab. or community navigation.kw. or community outreach.ti,ab. or community outreach.kw. or community health.ti,ab. or community health.kw. or home care.ti,ab. or home care.kw. or intervention*.ti,ab. or intervention*.kw. or exp Primary Health Care/ or integrated primary care.ti,ab. or integrated primary care.kw. or integrated care.ti,ab. or integrated care.kw. or citizen assistance.ti,ab. or citizen assistance.kw. or befriending.ti,ab. or befriending.kw. or assistance.ti,ab. or assistance.kw. or public health.ti,ab. or public health.kw.
4. "Review".pt. or systematic review*.ti,ab. or systematic review*.kw. or rapid review*.ti,ab. or rapid review*.kw. or scoping review*.ti,ab. or scoping review*.kw. or review*.ti,ab.
5. (2009* or 2010* or 2011* or 2012* or 2013* or 2014* or 2015* or 2016* or 2017* or 2018* or 2019* or 2020* or 2021* or 2022*).dp.
6. 1 and 2 and 3 and 4 and 5

**Résultats: 2404**

## EMBASE

1. aged/ or very elderly/ or frail elderly/ or (aged person* or aged people* or elder* or "late life" or senior* or older adult* or "65 years" or "66 years" or "67 years" or "68 years" or "69 years" or "70 years" or "71 years" or "72 years" or "73 years" or "74 years" or "75 years" or "76 years" or "77 years" or "78 years" or "79 years" or "80 years" or "81 years" or "82 years" or "83 years" or "84 years" or "85 years" or "86 years" or "87 years" or "88 years" or "89 years" or "90 years" or "91 years" or "92 years" or "93 years" or "94 years" or "95 years" or "96 years" or "97 years" or "98 years" or "99 years" or "100 years" or "101 years" or "102 years" or "103 years" or "104 years" or "105 years" or "106 years" or "107 years" or "108 years" or "109 years" or "110 years").tw,kw.
2. (poverty or lonel* or frail* or "social isolation" or "social alienation" or "functional impairment" or prefrail*).kw,tw. or exp Frailty/ or exp Loneliness/ or Poverty/ or exp Social Isolation/ or exp Social Alienation/
3. ("community integration" or "community navigation" or "community outreach" or "community health" or "home care" or intervention* or "integrated primary care" or "integrated care" or "citizen assistance" or befriending or assistance or "public health").kw,tw. or exp Community Integration/ or exp Community Health Planning/ or exp Therapeutic Community/ or exp Community Psychiatry/ or exp Mental Health Services/ or exp Community Medicine/ or Community Care/ or exp Community Health Nursing/ or exp Home Care/ or exp Parish Nursing/ or exp Primary Health Care/
4. "review"/ or "systematic review"/ or systematic review*.ti,ab. or systematic review*.kw. or rapid review*.ti,ab. or rapid review*.kw. or scoping review*.ti,ab. or scoping review*.kw. or review*.ti,ab.
5. (2009* or 2010* or 2011* or 2012* or 2013* or 2014* or 2015* or 2016* or 2017* or 2018* or 2019* or 2020* or 2021* or 2022*).dp.
6. 1 and 2 and 3 and 4 and 5

**Résultats: 504**

## EBM-Reviews

1. ("aged person" or "aged people" or elder* or elderly or "late life" or senior* or "older adult" or older adults).ti,ab. or ("aged person" or "aged people" or elder* or elderly or "late life" or senior* or "older adult" or older adults).kw.
2. (poverty or lonel* or frail* or "social isolation" or "social alienation" or "functional impairment" or prefrail*).ti,ab. or (poverty or lonel* or frail* or "social isolation" or "social alienation" or "functional impairment" or prefrail*).kw.
3. ("community integration" or "community navigation" or "community outreach" or "community health" or "home care" or intervention* or "integrated primary care" or "integrated care" or "citizen assistance" or befriending or assistance or "public health").ti,ab. or ("community integration" or "community navigation" or "community outreach" or "community health" or "home care" or intervention* or "integrated primary care" or "integrated care" or "citizen assistance" or befriending or assistance or "public health").kw.
4. systematic review*.ti,ab. or systematic review*.kw. or rapid review*.ti,ab. or rapid review*.kw. or scoping review*.ti,ab. or scoping review*.kw. or review*.ti,ab.
5. (2009* or 2010* or 2011* or 2012* or 2013* or 2014* or 2015* or 2016* or 2017* or 2018* or 2019* or 2020* or 2021* or 2022*).dp.
6. 1 and 2 and 3 and 4 and 5

**Résultats: 17**

## CINAHL

| **#** | **Question** |
| --- | --- |
| S1 | MH(“aged” or "aged, 80 and over" or “frail elderly”) OR (aged person* OR aged people* OR elder* OR "late life" OR senior* or "80 and over" or "65 years" OR "66 years" OR "67 years" OR "68 years" OR "69 years" OR "70 years" OR "71 years" OR "72 years" OR "73 years" OR "74 years" OR "75 years" OR "76 years" OR "77 years" OR "78 years" OR "79 years" OR "80 years" OR "81 years" OR "82 years" OR "83 years" OR "84 years" OR "85 years" OR "86 years" OR "87 years" OR "88 years" OR "89 years" OR "90 years" OR "91 years" OR "92 years" OR "93 years" OR "94 years" OR "95 years" OR "96 years" OR "97 years" OR "98 years" OR "99 years" OR "100 years" OR "101 years" OR "102 years" OR "103 years" OR "104 years" OR "105 years" OR "106 years" OR "107 years" OR "108 years" OR "109 years" OR "110 years") |
| S2 | Poverty OR lonel* OR OR frail* OR OR social isolation OR social alienation OR functional impairment OR prefrail* OR MH("Frailty Syndrome" OR "Loneliness" OR "Poverty" OR "Social Isolation+" OR "Social Alienation") |
| S3 | community integration OR community navigation OR community outreach OR community health OR home care OR intervention* OR integrated primary care OR integrated care OR intervention* OR citizen assistance OR befriending OR assistance OR public health OR MH("Community Mental Health Nursing" OR "Community Mental Health Services" OR "Health Care Delivery, Integrated" OR "Community Health Nursing" OR "Community Medicine" OR "Community Health Services" OR "Home Health Aides" OR "Parish Nursing" OR "Home Health Care" OR "Primary Health Care") |
| S4 | (MH "Systematic Review") OR systematic review* or rapid review* or scoping review* or review* |
| S5 | PY 2009-2022 |
| S6 | S1 AND S2 AND S3 AND S4 AND S5 |

**Résultats: 536**

## PsycInfo

1. (older people or older adult* or aged person* or aged people* or elder or elders or late life or "80 and over" or 65 year* or 66 year* or 67 year* or 68 year* or 69 year* or 70 year* or 71 year* or 72 year* or 73 year* or 74 year* or 75 year* or 76 year* or 77 year* or 78 year* or 79 year* or 80 year* or 81 year* or 82 year* or 83 year* or 84 year* or 85 year* or 86 year* or 87 year* or 88 year* or 89 year* or 90 year* or 91 year* or 92 year* or 93 year* or 94 year* or 95 year* or 96 year* or 97 year* or 98 year* or 99 year* or 100 year* or 101 year* or 102 year* or 103 year* or 104 year* or 105 year* or 106 year* or 107 year* or 108 year* or 109 year* or 110 year*).ti,ab.
2. (poverty or lonel*).ti,ab. or "Health Impairments"/ or frail*.ti,ab. or frail*.ti,ab. or exp Loneliness/ or exp Poverty/ or exp Social Isolation/ or social isolation.ti,ab. or social alienation.ti,ab. or functional impairment.ti,ab. or prefrail*.ti,ab.
3. exp Community Involvement/ or exp Therapeutic Community/ or Community Psychiatry/ or exp Community Mental Health Services/ or Community Health/ or community integration.ti,ab. or community navigation.ti,ab. or community outreach.ti,ab. or community health.ti,ab. or home care.ti,ab. or intervention*.ti,ab. or exp Primary Health Care/ or integrated primary care.ti,ab. or integrated care.ti,ab. or citizen assistance.ti,ab. or befriending.ti,ab. or assistance.ti,ab. or public health.ti,ab.
4. exp "Systematic Review"/ or systematic review*.ti,ab. or rapid review*.ti,ab. or scoping review*.ti,ab. or review*.ti,ab.
5. (2009* or 2010* or 2011* or 2012* or 2013* or 2014* or 2015* or 2016* or 2017* or 2018* or 2019* or 2020* or 2021* or 2022*).dp.
6. 1 and 2 and 3 and 4 and 5

**Résultats: 255**

## 1.7 Cochrane Library

#1 MeSH descriptor: [Community Integration] explode all trees 14

#2 MeSH descriptor: [Community Health Planning] explode all trees 50

#3 MeSH descriptor: [Therapeutic Community] explode all trees 53

#4 MeSH descriptor: [Community Psychiatry] explode all trees 21

#5 MeSH descriptor: [Community Mental Health Services] explode all trees 754

#6 MeSH descriptor: [Community Medicine] explode all trees 39

#7 MeSH descriptor: [Community Health Services] explode all trees 15216

#8 MeSH descriptor: [Community Health Nursing] explode all trees 353

#9 MeSH descriptor: [Home Health Nursing] explode all trees 9

#10 MeSH descriptor: [Parish Nursing] explode all trees 1

#11 MeSH descriptor: [Community Pharmacy Services] explode all trees 290

#12 MeSH descriptor: [Home Care Services] explode all trees 2573

#13 ((community) N5 (integration or navigation or outreach or care or health or intervention or assistance)):ti,ab,kw 4

#14 ((primary) N5 (integration or navigation or outreach or care or health or intervention or assistance)):ti,ab,kw 10

#15 ((public) N5 (integration or navigation or outreach or care or health or intervention or assistance)):ti,ab,kw 0

#16 befriend*:ti,ab,kw 145

#17 citizen*:ti,ab,kw 2992

#18 #1 OR #2 OR #3 OR #4 OR #5 OR #6 OR #7 OR #8 OR #9 Or #10 or #11 OR #12 OR #13 OR #14 OR #15 OR #16 OR #17 18470

#19 MeSH descriptor: [Frailty] explode all trees 334

#20 MeSH descriptor: [Poverty] explode all trees 1942

#21 MeSH descriptor: [Loneliness] explode all trees 153

#22 MeSH descriptor: [Social Isolation] explode all trees 353

#23 MeSH descriptor: [Social Alienation] explode all trees 20

#24 (poverty or frail* or lonel* or isolated or alienated):ti,ab,kw 23421

#25 #19 OR #20 OR #21 OR #22 OR #23 OR #24 23596

#26 MeSH descriptor: [Aged] explode all trees 221552

#27 MeSH descriptor: [Frail Elderly] explode all trees 804

#28 MeSH descriptor: [Aged, 80 and over] explode all trees 56150

#29 (old* or aged or late-life or elder):ti,ab,kw 639564

#30 #26 OR #27 OR #28 OR #29 639565

#31 #18 AND #25 AND #30 with Cochrane Library publication date Between Jan 2009 and Dec 2022

**Résultats: 345**
