# Supplementary material for: Effectiveness of interventions to address different types of vulnerabilities in community‐dwelling older adults: An umbrella review
Source: Campbell Syst Rev. 2023 May 9;19(2):e1323. doi: 10.1002/cl2.1323 (PMC10168691; doi:10.1002/cl2.1323)
Supplement: Supplementary file 2 — Supporting information. [file CL2-19-e1323-s001.docx]

| **Interventions**  Effect of non-pharmacological strategies to improve walking speed among community-dwelling older adults | **Author and year** | **Effect on walking speed** | **Magnitude of the effect** |
| --- | --- | --- | --- |
| **Systematic reviews** | | | |
| **Physical activity interventions** | Theou 2011 | 9 trials (n=686)  - 7 RCTs: **significant between group differences**  - 2 RCTs: no significant between groups differences | Not reported  - |
|  | Frost 2017 | 3 trials (n=188): no significant between groups differences | Mean Difference: -0.06 95% CI: -0.49 to 0.37, p=0.79, I^2^ = 50% |
|  | Burton 2019 | 2 RCTs (n=312): Effect in favor of the control group | Mean Difference: 0.02 95% CI: 0.02 to 0.02, p<0.00001, I^2^ = 0% |
| **Dietary and physical activity interventions** | Dedeyne 2017 | 3 trials (n=305-354)  - 1 RCT:  * **The % improvement in walking speed was significantly higher with [Exercise + nutritional supplementation of milk fat globule membrane] compared to nutritional supplementation of milk fat globule membrane**.  * **The % improvement in walking speed was significantly higher with [Exercise + nutritional supplementation of milk fat globule membrane] compared to nutritional supplementation of proteins**.  - 1 RCT: **[Exercise + nutritional supplementation of proteins + nutritional supplementation of vitamins and minerals]: significantly improved walking speed compared to [nutritional supplementation of proteins + nutritional supplementation of vitamins and minerals] or control group**.  - 1 RCT: no significant between groups differences | *****(% change =14.7±4.1, CI =6.4; 23.1) vs (% change =2.1±1.9, CI =-1.8; 5.9) (p<0.05)  *****(% change =14.7±4.1, CI =6.4; 23.1) vs (% change =3.6±2.7, CI =-1.9; 9.1) (p<0.05)  (0.06±0.1) vs (0.0±0.04) (p<0.01) |
|  | Liao et al. (2018) | 8 RCTs **: no significant difference between intervention and control group following protein supplementation and exercise training | SMD = 0.32, 95% CI =  -0.08; 0.73, I^2^ = 80%, |
| **Physical therapy interventions** | Arantes 2009 | 6 trials (n=735)  - 1 clinical trial/CT/RCT: **Strength gain was associated to change in gait speed.**  - 1 clinical trial/CT/RCT: **Intervention group exhibited significant improvement in gait**  **speed (p<0.001)**.  - 1 clinical trial/CT/RCT: no between group difference at 3 months. **Gait speed improved in the intervention group at 9 months (p=0.022).**  - 3 clinical trials/CT/RCT: no significant between groups differences | β=0.8 (p=0.02)  Not reported  Not reported  - |
| **Reablement, Reactivation, Rehabilitation and Restorative**  **Interventions** | Sims Gould 2017 | 1 RCT (n=205): **Significant improvement in favor of the intervention group** | Not reported |
| **Dietary and physical activity and cognitive interventions** | Dedeyne 2017 | 1 RCT (n=246): no significant between group differences | **-** |
| **Physical activity and nutritional advise** | Dedeyne 2017 | 2 RCTs (n=185): no significant between group differences | **-** |
| **Dietary and physical activity and hormones** | Dedeyne 2017 | 1 RCT (n=99): no significant between group differences | **-** |
| **Other reviews** | | | |
| **Dietary interventions** | Anton 2017 | 8 trials (n=864).  - 1 controlled clinical trial: **protein supplementation maintained walking speed in frail older adults compared to a reduction in the control group over a 12-week period.**  - 7 controlled clinical trials: no significant between group differences. | (-0.004) vs (- 0.043) (p<0.05)  - |
|  | Kelaiditi 2014 | 1 RCT (n=87): **Reduction of usual gait speed was significantly lower in the intervention group compared to the control group.** | (-1.0%) vs (-11.3%)  (p=0.04) |
| **Physical activity interventions** | Anton 2017 | 10 trials (n=705)  - 1 controlled clinical trials: **significant improvements in gait speed in the intervention group (functional circuit training) compared to the control group (health education meeting)**  - 1 controlled clinical trials:  **significant improvements in gait speed in the intervention group (resistance exercise training + balance) compared to the control group**  - 1 controlled clinical trials:  **significant improvements in gait speed in the intervention group (resistance exercise training + balance) compared to the control group**  - 7 controlled clinical trials: no significant between group differences | 0.12 vs (-0.02) (p<0.05)  0.07 vs 0 (p<0.05)  0.14 vs 0.10 (p<0.05)  - |
|  | Kelaiditi 2014 | 2 trials (n=264)  - 1 RCT: **Faster 4-m gait speed in the intervention group compared to control at the end of follow-up**  - 1 RCT: **significant improvements at 6-min walk test in intervention group compared to control** | +0.05 m/s, 95% CI=0.0004–0.1  Not reported |
|  | Puts 2017 | 1 RCT (n=51): **the intervention had greater improvements in rapid gait speed tests and those results were maintained at week 36 and significant** | Not reported |
| **Dietary and physical activity interventions** | Anton 2017 | 7 trials (n=422)  - 1 controlled clinical trials: **significant improvements in gait speed in the intervention group (aerobic exercise training + resistance exercise training + amino-acid supplementation) compared to the control group (health education)**  - 1 controlled clinical trials:  **significant improvements in gait speed in the intervention group (aerobic exercise training + resistance exercise training + flexibility exercise training + calorie restriction) compared to the control group**  - 5 controlled clinical trials: no significant between group differences | 0.16 vs 0.03 (p<0.05)  0.08 vs (-0.02) (p<0.05)  - |
|  | Puts 2017 | 1 RCT (n=89): no significant between group differences | - |

**Abbreviations**: CI: confidence interval, RCT: randomized controlled trial, SMD: standardized mean difference.

**Note:** Significant between-group differences are shown in bold, **Sample size not reported

Effect of non-pharmacological strategies to improve grip strength among community-dwelling older adults

| **Interventions** | **Author and year** | **Effect on grip strength** | **Magnitude of the effect** |
| --- | --- | --- | --- |
| **Systematic reviews** | | | |
| **Physical activity interventions** | Burton 2019 | 3 RCTs (n=363): no significant between group differences | Mean Difference: 0.07 95% CI: -1.52 to 1.65, p=0.93, I^2^ = 0% |
|  | Liam 2020 | 1 RCT (n=127): improvement in mean difference of hand grip strength at 6 months and at 12 months, favoring the resistance exercise group. | Mean Difference (6 months): 1.6 kg (95% CI 0.6–2.5)  Mean Difference (12 months): 3.1 kg (95% CI 1.9–4.4) |
| **Physical therapy interventions** | Arantes 2009 | 1 clinical trial/CT/RCT (n=87): no significant between group differences | - |
| **Dietary and physical activity interventions** | Frost 2017 | 1 RCT (n=89): no significant between group differences | **-** |
|  | Liao 2018 | 7 RCTs**: **significant difference in favor of the intervention group following protein supplementation and exercise training.** | SMD = 0.18, 95% CI = 0.01; 0.36, p=0.04, I^2^=24% |
| **Reablement, Reactivation, Rehabilitation and Restorative**  **Interventions** | Sims Gould 2017 | 1 RCT (n=61): no significant between group differences | **-** |
| **Other reviews** | | | |
| **Dietary interventions** | Anton 2017 | 3 controlled clinical trials (n=552): no significant between group differences | **-** |
|  | Kelaiditi 2014 | 1 RCT (n=87): no significant between group differences | - |
| **Dietary and physical activity interventions** | Anton 2017 | 2 controlled clinical trials (n=116): no significant between group differences | - |
|  | Puts 2017 | 1 RCT (n=89): no significant between group differences | - |

**Abbreviations**: CI: confidence interval, RCT: randomized controlled trial, SMD: standardized mean difference.

**Note:** Significant between-group differences are shown in bold, **Sample size not reported

Effect of non-pharmacological strategies to improve body weight among community-dwelling older adults

| **Interventions** | **Author and year** | **Effect on body weight** | **Magnitude of the effect** |
| --- | --- | --- | --- |
| **Systematic reviews** | | | |
| **Physical activity interventions** | Theou 2011 | 4 trials (n=389)  - 1 RCT: **significant between group difference**  - 3 RCTs: no significant between group differences | Direction and magnitude not reported |
|  | Arantes 2009 | 3 trials (n=480)  - 1 clinical trial/CT/RCT (n=217): **Increase in total body weight in intervention group over control group (p=0.041)**  -2 clinical trials/CT/RCT: no significant between group differences | Not reported |
| **Dietary and physical activity interventions** | Liao 2018 | 6 RCTs**: **significant difference in favor of the intervention group following protein supplementation and exercise training.** | SMD = 0.58, 95% CI = 0.25; 0.91, p=0.0006, I^2^=65% |
| **Other reviews** | | | |
| **Dietary interventions** | Anton 2017 | 7 controlled clinical trials (n=440): no significant between group differences | **-** |
| **Dietary and physical activity interventions** | Anton 2017 | 3 trials (n=436)  - 1 controlled clinical trials: **significant increase in body weight in the intervention group (resistance exercise training + proteins supplementation) compared to the control group (resistance exercise training + placebo)**  - 1 controlled clinical trials:  **significant increase in body weight in the intervention group (aerobic exercise training + resistance exercise training + balance + flexibility exercise training + calorie restriction) compared to the control group**  - 4 controlled clinical trial: no significant between group differences | 1.12 vs (-0.89) (p<0.05)  8.2±5.7 vs 0.7±2.7 (p<0.05)  - |

**Abbreviations**: RCT: randomized controlled trial, SMD: standardized mean difference. **Note:** Significant between-group differences are shown in bold, **Sample size not reported

Effect of non-pharmacological strategies to improve physical activity among community-dwelling older adults

| **Interventions** | **Author and year** | **Effect on physical activity** | **Magnitude of the effect** |
| --- | --- | --- | --- |
| **Systematic reviews** | | | |
| **Physical activity interventions** | Theou 2011 | 2 trials (n=501): significant between group differences  - 1 RCT: **the mean increase in physical activity was higher in the intervention group (Physical Activity) compared to the control group (Successful Aging) from baseline to 6 months and from baseline to 12 months**  - 1 RCT: **the mean weekly number of outdoor walks was higher in the combined training group (group trainings with functional balance and strength exercise) compared to the home training group (functional balance and strength exercise + group meetings) at 9 months** | *At 6months*:  112.66 minutes vs 18.94 minutes (p<0.001)  *At 12 months*:  63.23 minutes vs 5.76 minutes (p<0.005)  4.6 (SD 3.0) vs 3.3 (SD 3.0) (p=0.016) |
|  | Frost 2017 | 1 RCT (n=23): No statistical comparison between groups (physical activity increased in Wii group). | - |
|  | Burton 2019 | 1 RCT (n=23): no significant between group differences | **-** |
| **Dietary and physical activity interventions** | Dedeyne 2017 | 2 trials (n=148)  - 1 RCT:  ***At post-intervention, mean increase in physical activity was higher in the [nutritional supplementation of proteins + nutritional supplementation of vitamins and minerals] group compared to the control group.**    ***At 6 months follow-up, mean increase in physical activity was higher in the [nutritional supplementation of proteins + nutritional supplementation of vitamins and minerals] group compared to the control group.**  - 1 RCT: no significant between group differences | *(mean change =96.2, CI =57.8; 134.7) vs (mean change =20.5, CI =-17.0; 58.1) (p<0.01)  *(mean change =110.1, CI =71.9; 148.2) vs (mean change =34.8, CI =-2.99; 72.6) (p<0.01)  - |
|  | Liao 2018 | 4 RCTs**: no significant difference between intervention and control group following protein supplementation and exercise training | SMD = 0.21, 95% CI =  -0.38; 0.80, I^2^ = 85% |
| **Physical activity and nutritional advise** | Dedeyne 2017 | 1 RCT (n=96): **[Exercise + nutritional advise] significantly improved physical activity compared to control group; p<0.05.** | Not reported |
| **Dietary and physical activity and nutritional advise** | Dedeyne 2017 | 1 RCT (n=100): **[Exercise + nutritional advise + nutritional supplementation of vitamins and minerals] significantly improved physical activity compared to [Nutritional advise + nutritional supplementation of vitamins and minerals].** | 485.6±98.1 vs 265.8±46.1 (p<0.001) |
| **Dietary and physical activity and hormones** | Dedeyne 2017 | 1 RCT (n=99): no significant between group differences | - |

**Abbreviations**: CI: confidence interval, RCT: randomized controlled trial, SMD: standardized mean difference. **Note:** Significant between-group differences are shown in bold, **Sample size not reported.
