# Supplementary material for: Effectiveness of interventions to address different types of vulnerabilities in community‐dwelling older adults: An umbrella review
Source: Campbell Syst Rev. 2023 May 9;19(2):e1323. doi: 10.1002/cl2.1323 (PMC10168691; doi:10.1002/cl2.1323)
Supplement: Supplementary file 3 — Supporting information. [file CL2-19-e1323-s007.docx]

**Supplementary material 2:** Interventions components

| **Interventions** | **Components** |
| --- | --- |
| Anton et al. (2017)  Diet and physical activity | Single interventions:   - Dietary interventions: dietary supplementation / dietary education / calorie restriction - Exercise interventions : resistance exercise training / functional circuit training   Multicomponent interventions:   - Multicomponent exercise interventions: resistance exercise training + balance + flexibility exercise training / aerobic exercise training + resistance exercise training + balance / aerobic exercise training + resistance exercise training + balance + flexibility exercise training / resistance exercise training + balance / walk + resistance exercise training + balance - Combined dietary and exercise interventions: aerobic exercise training + resistance exercise training + balance + dietary education / resistance exercise training + dietary supplementation / aerobic exercise training + resistance exercise training + balance + dietary supplementation / resistance exercise training + dietary supplementation / resistance exercise training + balance + dietary supplementation / aerobic exercise training + resistance exercise training + balance + flexibility exercise training + calorie restriction / walk + resistance exercise training + balance + dietary supplementation / resistance exercise training + placebo |
| Arantes et al. (2009)  Physical activity | Single interventions   - muscle strengthening - functional training - water exercise   Multicomponent interventions   - exercises for muscle strengthening, balance, coordination, flexibility, reaction time and aerobic training - physical therapy (balance exercises + coordination + flexibility + strengthening + reaction time) - at-home physical therapy (environment adaptation + prescription of assistive devices + exercise) - environment adaptation and prescription of assistive device |
| Burton et al. (2019)  Physical activity for older adults receiving home care services | Single interventions   - Repetitive ADL exercise - Supervised progressive strength training programme   Multicomponent interventions   - Multicomponent exercise interventions: balance + lower limb strength exercises/ 35-minute DVD that includes a warm-up + upper and lower extremity exercises using therabands for resistance + cool down/ balance and strength exercises/ arm curls 2 times (holding a 1-pound weight, or a soup can or water bottle) + ankle point and flex up to 30 seconds on each foot + seated step-in-place - Home care services including domestic tasks, personal care, shopping, and individualized activities (walking or exercise programs/other activities aimed at improving functional ability) - Progressive multicomponent physical therapy intervention consisting of ADL training (bathroom transfers, bed mobility, and car transfers), evidence-based mobility training (indoor walking, gait training on flat ground and stairs, and outdoor walking transitioning to an independent daily walking program) and progressive strength training (3 sets 8 repetitions max supine leg press, standing hip extension, body-weight resisted plantar flexion, seated press and seated row using the Shuttle Mini Press). Participants were given a home exercise program to continue basic exercises after discharge - Individualized reablement program including training in daily activities (dressing, food preparation, vacuuming, bus transport), adaptations to the environment or the activity to simplify activity performance, exercise programs (indoor or outdoor walking with or without walking aids, climbing stairs, transferring, and performing exercises to improve strength, balance or fine motor skills) and a manual explaining each of the exercises for self-training - MAHC-10 fall risk assessment tool, medication review process and management plan, home safety assessment, patient- and family-specific education, individualized home-based exercise regime - Individualized home exercise program designed to address impairments and home safety/mobility adaptation - Flexibility, strength, balance, and aerobic exercise program with pain management |
| Franck et al. (2016) Interventions addressing social isolation and depression | Single intervention   - Reminiscence therapy - Gender-based club meetings and activities - Playing a Wii game of choice - Listening to the radio program   Multicomponent interventions   - Gardening activities and education |
| Frost et al. (2017) Home and community-based health promotion interventions | Single interventions   - Resistance exercise - Telemonitoring - Seated exercise   Multicomponent interventions   - Multicomponent exercise interventions: Balance and strength exercises/ Power training + strength training/ Strength and balance training alone - Nintendo Wii basic games + weight vest carried out - Strength + balance training + nutrition |
| Hagan et al. (2014)  Social therapeutic interventions | Components of the interventions were not reported   - Community Connections’ group (older people and international students) - MBSR group programme - LUSTRE six-week group programme - Friendship Enrichment 12-week group programme - Nurse led 12-week psychosicial group work - Senior Companion Program befriending scheme - Community-based mentoring service - Mentoring service + community activities |
| Kelaiditi et al. (2014)  Diet and exercise | Single interventions   - Dietary interventions: dietary supplementation - Exercise interventions: seated exercise   Multicomponent interventions   - Multifactorial, personalized home exercise program against sedentariness (8 physical therapy sessions) - Wii-fit: electronic games + exercise group sessions (45 min/ 3 times per week/15 Weeks) - Combined dietary, exercise interventions and psychotherapy: Nutritional consultation + exercise (brisk walks, resistance, postural, and balance training) + problem-solving therapy |
| Khoshravi et al. (2016) Technological interventions | Single interventions   - General ICTs: computer and/or internet training and use - Use of social network sites - Robotics: robot, relational agents/ pet robot/ mobile presence system/ computer conversational agent-based system - Video games - Personal Reminder Information and Social Management System (special software designed for seniors) Asynchronous peer support chat rooms, - Tele-Care: video-telephone nursing care/ care TV - 3D virtual environments |
| Liao et al. (2018) Diet and exercise | Single interventions   - Protein Supplementation with different protocols: Regarding the amount of protein ranging from 4.1 to 40.8 g/day; with extra protein ranging from 6.0 to 41.4 g and supplementation of milk-based beverages, fortified milk, and milk protein concentrate or a combination of whey protein, leucine, and essential amino acids. - Resistance Exercise Training   Multicomponent interventions   - Exercise Training with different protocols: Multicomponent exercise training with moderate to high intensity - Combination of protein supplementation and exercise training |
| Looman et al. (2019) Preventive, integrated care interventions | Details about components of the interventions were not reported |
| Pool et al. (2017) Physical, educational and volunteering activities | Single interventions   - Educational activity (15 lessons on topics) - Physical activity: Tai Chi exercise intervention   Multicomponent interventions   - Volunteering activity: Volunteering on elementary school classrooms + training of volunteers + volunteers team meetings (for discussion, problem-solving, planning, socializing, refresher training) - Physical activity + educational activity: home-based exercise + advice/education group sessions + one to one feedback - Physical activity + educational activity: exercise + goal setting + discussions about ways to modify environment |
| Shvedko et al. (2018)  Physical activity interventions | Single interventions   - Exercise interventions: strength exercise/ aerobic exercise/ functional tasks exercise/ resistance exercise/ Home exercise programme (isometric and isotonic quadriceps exercise)/ walking exercise/ single isometric back strength exercise/ Dietary weight-loss: consultations with the dietitian/ Exercises with elastic bands/ Tai Chi/ balance training/ Yoga   Multicomponent interventions   - Multicomponent exercise interventions: Aerobic + muscle strength + stretching / Tai Chi Qigong + advise on individual practice at home/ breathing + walking exercise/ aerobic exercise + resistance training/ aerobic + strengthening + flexibility exercises/ balance + resistance + strengthening exercises + walking/ walking + cycling + strength exercises - Exercise interventions with social interactions: Water-based and mixed land-based exercise + education/ exercise + social support/ exercise + health education/ Exercise + cognitive behavioural therapy/ community-based group-exercise programme/ Exercise + recreational activity/ Exercise + sleep education/ Exercise + education/ Exercise + support/ Exercise + telephone calls - Diet + exercise   Not clear: Home-based personalised exercise programme (preferred mode e.g. swimming, cycling) |
| Sims- Gould et al. (2017)  4R interventions (rehabiltiation, restorative, reablement, reactivation) | Multicomponent interventions   - Geriatric assessment, coordination with the primary care system, regular interdisciplinary geriatric care team - Combination of a center-based physical activity program, a home-based program focused on functional tasks (eg, cooking, cleaning), and a brief educational component regarding falls prevention, medication management, community services, etc - Personalized exercise plan, development of health-promoting strategies, home visit, and regular phone calls - Early discharge and rehabilitation service (EDRS). Assessment and monitoring, rehabilitation therapy, and care assistance - In-home rehabilitation, assistance with routines. Training and support for the carers at home. Creation of a support network that the client would require on completion of the intervention - OT or PT worked with client to develop rehabilitation plan that reflects goals of client. Training in daily activities, exercise plan, and at least 1 h/ wk of physical or occupational therapy - Home Independence Program. Task redesign, home modification, exercises and various types of self-care/ self-management - Home treatment team (HTT). Up to 3 daily visits for 6 wk, with an emphasis on home-based rehabilitation - Dutch EASYcare. Individualized, integrated treatment plan. Plans focused on cognition, mood, behavior, nutrition, and mobility - Tailored to individual. Could include exercise, behavioral change, environmental modifications, training for clients and carers, and medication adjustments   Not clear:   - Assessor and client used a goal facilitation tool to develop short- and long-term goals. The goals then informed a support plan, executed by home care, following a restorative home care model - Promoting Independence Programmes (PIP) offer case-managed restorative home care, delivered by a multidisciplinary team in home and residential care |
| Puts et al. (2017) Multiple interventions | Single interventions   - Exercise interventions - home modifications   Multicomponent interventions   - Exercise + nutrition interventions - Combination intervention (exercise, nutrition and cognition training) - Prehabilitation therapy (PT) + exercise + home modifications and geriatric assessment |
| Dedeyne et al. (2017)  Multiple  interventions | Multicomponent interventions   - Nutritional + physical activity intervention - Combined hormone therapy with physical activity intervention - Combined exercise, nutritional, and hormone intervention - Exercise and nutritional intervention with psychotherapy - Combined exercise, nutritional, and cognitive interventions |
| Social capital interventions  Coll-Planas et al. (2017) | The authors were not explicit about the definition and the components of social capital interventions   - only existing social relationships - only new social relationships - both new and existing social relationships - only structural social capital - only cognitive social capital - both structural and cognitive - total structural social capital - total cognitive social capital - total bonding - total linking - total bridging |
| Snodwen et al. (2015)  Physical activity, social support and skill training | The authors did not provide enough details about the components of some interventions   - Skills training: included self-management [Chronic Disease Self-Management Program (CDSMP)], psychoeducation, anger management and stress management interventions - Social support: included interventions targeting direct or indirect provision of social support (interventions designed to improve ability to obtain support)   Single interventions:   - Motivation/counseling - Physical activity interventions: aerobic activity/strength training/balance/flexibility interventions   Multicomponent interventions:   - Social support + skills training - Physical activity interventions: combination of exercise types. |
| Cohen- Mansfield & Perach (2015)  Interventions for Alleviating Loneliness Among  Older Persons | Single and multicomponent interventions   - Group interventions: Educational focus with or without psychosocial element (involving a geriatric rehabilitation program, a focus on self-management abilities, coping skill education and computer training); Shared activities (visual art discussions, forms of aerobic activity, choral participation and foster grand-parenting). - One on one interventions: Educational focus with psychosocial element (focusing on caregiver relationship, personal mentoring, telephone crisis program), with technological focus (computer training and computerized relational agent and visits from occupational therapist); Specific Therapy Techniques and Sensory technological aids (hearing aids use). |
| Theou et al. (2011)  Exercise interventions | Single interventions   - Home-based resistance training - Resistance training - Progressive resistance training - (mobility task specific and one component at the fastest possible velocity) - Horse riding simulator training.   Multicomponent interventions   - Multicomponent training (resistance, balance, and flexibility) - Supervised group functional multicomponent training (aerobic, resistance, flexibility, speed, coordination, and skills training) - Class-based multicomponent training (functional aerobic, resistance, and flexibility) - Multicomponent (physical therapy, aerobic, and resistance) - Multicomponent training (aerobic, resistance, and Qiqong) - Low intensity supervised multicomponent training (resistance, balance, flexibility, body handling skills, speed of reaction, and coordination) - Functional multicomponent training (resistance, balance) - Home-based multicomponent training (aerobic, resistance, flexibility, and dynamic balance) - Multicomponent training (aerobic, resistance, balance, flexibility, and walking) - Multicomponent training (resistance, balance, and walking) - Multicomponent and comprehensive training (aerobic, resistance, balance, flexibility, rhythm, and reaction). |
| Tricco et al. (2022)  Intervention for social isolation and loneliness | Chorale intervention   - attendance at weekly singing rehearsals and several public performances   ElderHelp Concierge Club intervention   - Community-based care model that provided different tiers of services to their members including information and referrals, transportation or in-house assessments.   Urban Health Centres Europe (UHCE)   - preventative multidimensional health assess- ment, which informed the coordination of specific care pathways targeting the individual’s needs (such as fall risk, appropriate medication use, loneliness and frailty).   Health Teams Advancing Patient Experience: Strengthening Quality (Health TAPESTRY) intervention   - Collection of information on patients’ health goals and needs by - Trained volunteers summarised these findings in a report for the interprofessional primary care team - The primary care team act on plans of care - community agencies and volunteers could help address each patient’s goal |
| Li et al. (2022)  Interventions in reducing loneliness | Single interventions   - Exercise interventions: Tai chi qigong practice, Baduanjin qigong, Multisensory stimulation exercise therapy for pain management - Health education, exercise - Psychological group counseling - Project-based learning - Self-efficacy therapy - Family doctor program - Psychological counseling - Satir Therapy - Social work intervention of elderly loneliness - Reminiscence group therapy   Multicomponent interventions   - Healthy ecology therapy: three-level comprehensive intervention of family, community and society - Nurse-led multidisciplinary home visiting program - Comprehensive intervention: a health service model of urban community with a family doctor team as the main line and their clinics as the platform |
| Smith et al. (2019)  Social prescribing interventions to reduce frailty | No interventions found |
| Ibrahim et al. (2022)  Social interventions | Single interventions   - Group singing: included repertoires of cultural and religious songs, singing rehearsals and public performances - Group training: Computer training on basic usage and access to the internet was friendship enrichment program, a self-management course and a peer-led health education program, applied emotional support through memories of personal belongings, training on resourcefulness in exploring opportunities to engage in social and cultural activities within their living areas and sharing the information they gathered within the community - Arts and crafts activities - Writing - Gardening - Technology based activities   Multicomponent interventions   - Group activities and personal/group monitoring - Technology based activities and group activities |
| Heins et al. (2021)  Technological interventions | Single interventions   - Computer training programs - Mobile reminiscing therapy app - Social network - Video games   Multicomponent interventions   - Personal Reminder Information and Social Management (PRISM) system - Technology-assisted self-monitoring of physical activity |
| Wister et al. (2021)  Technological interventions | Single interventions   - Hearing aids and cochlear implants - At-home nursing telecare program - At-home tablet-based exercise program - Relational agents (artificial intelligence) - In-person or At-home group computer or tablet competence training (learning basic computer and tablet skills(turning the computer or tablet on and off, using a keyboard and mouse, become familiar with certain ap- plications, how to use a touch screen), how to write documents, how to use the internet and search for information, how to use email, online safety, online shopping, social media instruction, as well as communicating with others via applications such as FaceTime and Skype) - Online support community for caregivers - At-home video-conferencing behavioural activation - Personal Reminder Information and Social Management (PRISM) system - Mobile app - Video games - Virtual reality support group - Social internet -based activities (SBA) - Animatronic pet - Online cognitive behavioural intervention for depression |
| Fu et al. (2022)  Remotely delivered interventions for loneliness | Single interventions   - Telephone - Internet |
