# Supplementary material for: Effectiveness of interventions to address different types of vulnerabilities in community‐dwelling older adults: An umbrella review
Source: Campbell Syst Rev. 2023 May 9;19(2):e1323. doi: 10.1002/cl2.1323 (PMC10168691; doi:10.1002/cl2.1323)
Supplement: Supplementary file 4 — Supporting information. [file CL2-19-e1323-s004.docx]

**Supplementary material 3**: Instruments used to measure primary outcomes

| **Authors** | **Outcomes** |
| --- | --- |
| Anton et al. (2017) | Frailty components (Fried phenotype): weight loss, gait/walking speed, grip strength |
| Arantes et al. (2009) | Frailty components (Fried phenotype): weight loss, gait speed, grip strength |
| Burton et al. (2019) | Frailty status (SPPB, Fried phenotype),  frailty components (Fried phenotype): grip strength, gait speed |
| Franck et al. (2016) | Social isolation (Lubben Social Network Scale (LSNS), two questions on social isolation  developed by the authors)  Loneliness (Revised University of California Los Angeles Loneliness Scale (RULS-V3), UCLA Loneliness Scale, single-question item adapted from Victor et al.) |
| Frost et al. (2017) | Frailty status: SPPB, frailty state transitions (which scale?),  Frailty components (Fried phenotype): handgrip strength, gait speed |
| Hagan et al. (2014) | Social isolation (Internally developed social support scale, MOS Social Support Survey, social provisions scale)  Loneliness (Self-developed satisfaction survey, UCLA, De Jong Gierveld) |
| Kelaiditi et al. (2014) | Frailty status (Fried phenotype and modified Fried phenotype) |
| Khoshravi et al. (2016) | Social isolation (Survey for social isolation in Social network Sites and depth interviews)  Loneliness (University of California Los Angeles (UCLA) Loneliness scale, de Jong Gierveld and Havens’Loneliness Scale) |
| Liao et al. (2018) | Frailty status (frailty index, global frailty score using the Fried phenotype, SPPB)  Frailty components (Fried phenotype): body weight, handgrip strength, gait speed, physical activity, exhaustion |
| Looman et al. (2019) | Frailty status (measurement method not reported),  Social isolation: social functioning, social support (measurement method not reported)  Adverse health outcomes: pain, fall, mortality, healthcare utilization, costs |
| Pool et al. (2017) | Social isolation: social support (The Multidimensional Scale of Perceived Social Support), social provision (Social Provision Scale), social activities (Likert scale, social activities. Questions regarding social activity/emotional support after 8 months, Lifestyle Activity Questionnaire. Interviews (social activity)),  loneliness (UCLA scale) |
| Shvedko et al. (2018) | Social isolation: social isolation (The Turkish version of the Nottingham Health Profile questionnaire), social support (revised social support questionnaire, Multidimensional Scale of Perceived Social Support (MSPSS), short version of the Medical Outcomes Study (MOS) Social Support Survey, Chinese version of the Inventory of Social Supportive Behaviours), social functioning (Short Form (SF-36) Health Survey (as a subdomain of health-related quality of life)), social networks (6-item Lubben's Social Network Scale (LSNS))  Loneliness (1-item question: “Do you feel lonely?”, Russel's UCLA loneliness scale and De Jong Gierveld Loneliness scale) |
| Sims- Gould et al. (2017) | Frailty status (SPPB)  Social isolation: social support (Duke Social Support Index),  Loneliness (De Jong and Kamphuis Loneliness Scale) |
| Puts et al. (2017) | Frailty status (Fried phenotype, adjusted Fried phenotype, Tinetti/Gill criteria, Japanese Frailty checklist, Frailty based on 3 measures: (i) the PPT, (ii) activities of daily living and (iii) measurement of peak oxygen uptake, Chinese Canadian Study of Health and Aging Clinical Frailty Scale Telephone Version, Frailty = a change in of care level, Frailty score >10 on the frailty checklist items 1–20, Physically frail which as determined by two tests (rapid gait speed) and single chair stand, Edmonton Frailty Scale, Barthel index) |
| Dedeyne et al. (2017) | Frailty status (Fried frailty phenotype, Physical performance test, Modified Fried frailty phenotype, Modified Chin A Paw frailty definition, Frailty Instrument for Primary Care of the Survey of Health, Ageing, and Retirement in (SHARE-FI), The Chinese Canadian Study of Health and Aging, Clinical Frailty Scale Telephone).  Social involvement |
| Coll-Planas et al. (2017) | Loneliness (UCLA Loneliness Scale (short version and version 3), Loneliness (Stroebe et al., 1996), 3-item Loneliness scale developed by Hughes, 7-item loneliness scale from Paloutzian and Ellison, De Jong Gierveld Loneliness Scale, Ando-Osada-Kodama (AOK) loneliness scale (revised version of the UCLA Loneliness Scale)) |
| Snodwen et al. (2015) | Loneliness (measurement method not reported) |
| Cohen- Mansfield & Perach (2015) | Social isolation (Social activities from the RAND Social Health Battery (one item on social participation), Items from the OARS social resource rating scale (custom item on satisfaction with socialization), Items tapping daily activities and social contact, Self-report of social Interactions)  Loneliness (UCLA-LS (Version 3 and revised), Philadelphia Geriatric Center Morale Scale (PGCMS) Lonely Dissatisfaction subscale, de Jong-Gierveld and Kamphuis’s questionnaire, ‘Do you feel yourself lonely?’’ and 2 additional items, Participants self-report of the impact of the intervention on their loneliness, Emotional/Social Loneliness Inventory (15 paired items)) |
| Theou et al. (2011) | - Frailty status (SPPB), - Frailty components: body weight, gait speed, physical activity |
| Walters et al. (2017) | Frailty status (Fried phenotype) |
| Wister et al. (2021) | Revised UCLA Loneliness Scale (Russell et al., 1980), the UCLA Loneli- ness Scale (Version 3) (Russell, 1996), and the Three-Item UCLA Loneliness Scale (Hughes et al., 2004). The de Jong Gierveld Loneliness Scale (de Jong Gierveld & Kamphuis, 1985), 6-item de Jong Gierveld Loneliness Scale (de Jong Gierveld & Van Tilburg, 2006), the 8-item PROMIS (Patient-Reported Outcomes Measurement Information System) Social Isolation Scale (PROMIS- L) (Riley et al., 2011).  4-item Social Interaction Subscale of the Duke Social Support Index (DSSI-I) (Landerman, George, Campbell, & Blazer, 1989), the Patient Reported Outcome Measurement Information System (PROMIS)- Social Isolation (6-item) (Riley et al., 2011.). |
| Tricco et al. (2022) | University of California, Los Angeles (UCLA) Loneliness Scale III  short De Jong Gierveld Loneliness Scale  Unnamed 3-item survey  Duke Social Support Index |
| Li et al. (2022) | Self-measured questionnaire to measure loneliness |
| Ibrahim et al. (2022) | NR |
| Smith et al. (2019) | No tools given that no interventions were identified |
| Heins et al. (2021) | UCLA Loneliness Scale, Medical Outcomes Study Social Support Survey, Three-item loneliness scale and Medical Outcome Social Support Survey (MOSS), De Jong Gierveld Loneliness Scale  Self-reported social network data, Lubben Social Network Scale, and Social Provisions Scale, Multidimensional Scale of Perceived Social Support (MSPSS), Duke Social Support Index (DSSI) |
| Fu et al. (2022) | UCLA Loneliness Scale, De Jong Gierveld Loneliness Scale (DJGLS), 3-point Likert scale |
