# Supplementary material for: Effectiveness of interventions to address different types of vulnerabilities in community‐dwelling older adults: An umbrella review
Source: Campbell Syst Rev. 2023 May 9;19(2):e1323. doi: 10.1002/cl2.1323 (PMC10168691; doi:10.1002/cl2.1323)
Supplement: Supplementary file 5 — Supporting information. [file CL2-19-e1323-s005.docx]

Methodological quality and overall confidence in the results of the reviews

| **Authors** | **1** | **2** | **3** | **4** | **5** | **6** | **7** | **8** | **9** | | **10** | **11** | | **12** | **13** | **14** | **15** | **16** | **Overall confidence** |
| --- | --- | --- | --- | --- | --- | --- | --- | --- | --- | --- | --- | --- | --- | --- | --- | --- | --- | --- | --- |
|  |  |  |  |  |  |  |  |  | RCT* | NRS |  | RCT | NRS |  |  |  |  |  |  |
| **Systematic reviews** | | | | | | | | | | | | | | | | | | | |
| Arantes et al. (2009) | Yes | No | Yes | Yes | Yes | Not clear | No | Yes | Yes | Include only trials | No | N/A | N/A | N/A | Yes | No | N/A | No | Critically low |
| Burton et al. (2019) | Yes | No | Yes | Yes | Yes | Not clear | No | Yes | Yes | Yes | No | Yes | N/A | Yes | Yes | Yes | No | Yes | Critically low |
| Frank et al.  (2019) | Yes | No | Yes | Yes | Yes | Yes | No | Yes | Yes | Yes | No | N/A | N/A | N/A | Yes | No | N/A | No | Critically low |
| Frost et al.  (2019) | Yes | Yes | Yes | Yes | Yes | No | No | Yes | Yes | Include only trials | No | Yes | N/A | Yes | Yes | Yes | N/A | Yes | Low |
| Khosravi et al. (2016) | Yes | No | Yes | Yes | Yes | Yes | No | Yes | No | No | No | N/A | N/A | N/A | No | No | N/A | No | Critically low |
| Liao et al.  (2018) | Yes | Yes | Yes | Yes | Yes | Yes | No | Yes | Yes | Include only trials | No | Yes | N/A | Yes | Yes | Yes | Yes | Yes | Low |
| Looman et al. (2019) | Yes | No | Yes | Yes | Yes | No | No | Yes | Partial yes | Partial yes | No | N/A | N/A | N/A | Yes | No | N/A | No | Critically low |
| Shvdeko et al. (2018) | Yes | Yes | Yes | Yes | Yes | Not clear | No | Yes | Yes | Include only trials | No | Yes | N/A | Yes | Yes | Yes | Yes | Yes | Low |
| Sims-Gould et al. (2017) | Yes | Yes | Yes | Yes | Yes | Yes | No | Yes | Yes | Include only trials | No | N/A | N/A | N/A | Yes | No | N/A | No | Low |
| Dedeyne et al. (2017) | Yes | No | Yes | Yes | Yes | Yes | No | Yes | Partial yes | Partial yes | No | N/A | N/A | N/A | Yes | No | N/A | Yes | Critically low |
| Coll-Planas et al. (2017) | Yes | Yes | Yes | Yes | Yes | Yes | No | Yes | Yes | Include only trials | No | N/A | N/A | N/A | Yes | No | N/A | Yes | Low |
| Snowden et al. (2015) | No | No | Yes | Partial yes | Not clear | Not clear | No | No | Partial yes | Partial yes | No | N/A | N/A | N/A | No | No | N/A | Yes | Critically low |
| Cohen- Mansfield & Perach et al. (2015) | Yes | No | Yes | Yes | Not clear | Not clear | No | Yes | No | No | No | N/A | N/A | N/A | No | No | N/A | No | Critically low |
| Theou et al. (2011) | Yes | No | Yes | Yes | Yes | Yes | No | No | No | No | No | N/A | N/A | N/A | No | No | N/A | Yes | Critically low |
| Walters et al. (2017) | Yes | Yes | Yes | Yes | Yes | No | No | Yes | Yes | No | No | Yes | N/A | Yes | Yes | No | No | Yes | Critically low |
| Fu et al. (2022) | Yes | Yes | No | Yes | Yes | Yes | No | Yes | Yes | Include only trials | No | Yes | N/A | No | No | No | Yes | Yes | Critically low |
| Heins et al. (2021) | Yes | Yes | Yes | Yes | Yes | Yes | No | Yes | Yes | Yes | No | N/A | N/A | N/A | No | No | N/A | Yes | Critically low |
| Li et al. (2022) | Yes | No | Yes | Yes | Yes | Yes | No | Yes | Yes | No | No | Yes | No | No | No | No | No | No | Critically low |
| Smith et al. (2019) | No studies were included in this review | | | | | | | | | | | | | | | | | | |
| Tricco et al. (2022) | Yes | Yes | Yes | Yes | Yes | Yes | No | Yes | Yes | Yes | Yes | N/A | N/A | N/A | No | No | N/A | Yes | Critically low |
| **Other reviews** | | | | | | | | | | | | | | | | | | | |
| Anton et al. (2017) | Yes | No | Yes | Yes | Not clear | Not clear | No | Yes | No | Include only trials | No | N/A | N/A | N/A | No | No | N/A | Yes | Critically low |
| Hagan et al. (2014) | Yes | No | Yes | Partial yes | Not clear | Not clear | No | Yes | No | No | No | N/A | N/A | N/A | No | No | N/A | No | Critically low |
| Kelaiditi et al. (2014) | Yes | No | Yes | Yes | Not clear | Not clear | No | Partial yes | No | No | No | N/A | N/A | N/A | No | Yes | N/A | Yes | Critically low |
| Pool et al.  (2017) | Yes | No | Yes | Yes | Yes | Not clear | No | Yes | Yes | Yes | No | N/A | N/A | N/A | Yes | No | N/A | Yes | Critically low |
| Puts et al.  (2017) | Yes | Yes | Yes | Yes | Yes | Yes | No | Yes | Yes | Yes | No | N/A | N/A | N/A | Yes | No | N/A | Yes | Low |
| Wister et al. (2021) | Yes | Yes | Yes | Yes | Yes | Yes | No | Yes | No | No | No | N/A | N/A | N/A | No | No | N/A | Yes | Critically low |
| Ibrahim et al. (2022) | Yes | No | Yes | No | Yes | Yes | No | Partially Yes | No | No | No | N/A | N/A | N/A | No | No | N/A | Yes | Critically low |

*RCT: Randomized Controlled Trials, NRS: non-randomized studies, N/A: Not Applicable.
