# Supplementary material for: Effectiveness of interventions to address different types of vulnerabilities in community‐dwelling older adults: An umbrella review
Source: Campbell Syst Rev. 2023 May 9;19(2):e1323. doi: 10.1002/cl2.1323 (PMC10168691; doi:10.1002/cl2.1323)
Supplement: Supplementary file 6 — Supporting information. [file CL2-19-e1323-s006.docx]

# Methodological quality/risk of bias assessment and overall quality of the evidence of included studies as reported by the review authors

| **Authors** | **Methodological quality or risk of bias assessment** | **Overall quality of the evidence** |
| --- | --- | --- |
|  |  |  |
| **Systematic reviews** | | |
| Arantes et al. (2009) | **Most of the articles (56%) had scores ≥5 on the PEDro scale, and thus were considered of high quality** (the scale has a total score of 10 points and score ranged from 2 to 8 points). | Not assessed |
| Burton et al. (2019) | Study quality for the RCTs was assessed using the Cochrane’s Risk of Bias Tool. In general, **the quality of the ten RCTs studies was rated “medium” to “high”, suggesting low risk of bias** (sequence generation: 9/10 studies; allocation concealment: 5/10 studies; blinding: 6/10 studies; low risk of incomplete outcome data: 9/10 studies; low risk of selective outcome reporting: 6/10 studies).  Study quality for all other included papers was reviewed using the National Institute for Health and Care Excellence (NICE) guidelines appraisal checklist. Similar to the RCTs, **the quality of the eight non-RCTs was between ++** (all or most of the checklist criteria have been satisfied, where they have not, it was deemed unlikely to alter the conclusions) **and +** (some of the checklist criteria have been satisfied, where they have not, it is unlikely these will alter the conclusions) **on the assessments, where it was deemed that most had satisfied the criteria for internal and external validity** (internal validity: ++ for 1/8 studies and + for 5/8 studies; external validity: ++ for 2/8 studies and + for 6/8 studies). It must be noted however, that all but one study did not have a comparison group, which meant a number of criteria were consequently not reported. | Not assessed |
| Frank et al.  (2019) | This review excluded all low quality studies in order to increase confidence in the findings of the reviewed interventions.  **Of the five discrete studies, only one was rated as high quality, with the remainder being of moderate quality** using the Downs and Black checklist for methodological quality assessment of randomized and nonrandomized studies of healthcare interventions (Items 1–26 on this checklist were considered in the  quality review. A high-quality study was quantitatively considered as having low risk of bias with a score of 19 or higher on the 26 items considered. A low-quality study was quantitatively considered as having a high risk of bias with a score of 13 or below). | Not assessed |
| Frost et al.  (2019) | Assessment of risk of bias was done using the Cochrane Risk of Bias tool.  **One study was at low risk of bias across all key domains. For two studies, the overall risk of bias was unclear, with some domains rated as low risk. In one study all key domains were of unclear bias risk due**  **to poor reporting, whilst three studies were at overall high risk due to high risk of bias in one or two**  **or more key domains**. | Not assessed |
| Khosravi et al. (2016) | Not clear | Not assessed |
| Liao et al.  (2018) | On the basis of the PEDro score, the methodological quality of the included RCTs was rated as high (≥7/10), medium (4–6/10), and low (≤3/10). **Of the 22 included RCTs, the methodological quality of 11 was classified as high and that of the other 11 as medium, with a median (range) PEDro score of 7/10 (4/10 to 9/10)**. | Level of evidence:  - Global frailty score: moderate  - Walking speed: conflicting  - grip strength: strong  - Body weight: moderate  - Physical activity: conflicting |
| Looman et al. (2019) | The quality of the included studies was assessed with the Effective Practice and Organization of Care (EPOC) risk of bias tool for studies with a separate control group (EPOC, 2015). The nine criteria are assessed in three categories: low risk (1 point), high risk (0 point) and unclear risk (0 point) and the total quality score ranges from 0 to 9. **Overall, the quality of the evidence was moderate ranging from 2 to 9 on the EPOC risk of bias scale with an average score of 5.3**. | Not assessed |
| Shvedko et al. (2018) | The risk of bias in individual studies was using the 12-criteria Cochrane Review Book Group (CRBG) risk of bias assessment tool using responses: yes, no, or unclear. Interventions meeting a minimum six criteria and above (scored as “Yes”using CRBG tool) and without serious flaws (e.g., over 20% drop-out rate in one intervention group) were rated as having a low risk of bias. Interventions with fewer than six criteria met were considered to have a high risk of bias.  **Risk of bias in individual studies revealed that 26 had a low risk of bias and 12 had a high risk of bias**. The most common biases were associated with inadequate allocation concealment and not described or non-acceptable compliance and drop-out rates. The intention-to-treat analysis was not reported in 24 interventions which limits the interpretation of the results in the included RCTs due to the low methodological rigour associated with the inadequate randomisation procedures. Detection bias in included studies was mainly high due to the absence of information about adequacy of outcome assessor blinding. Another source of bias was inadequate blinding of the care provider (fitness or medical healthcare provider) but it is likely this is not possible for this type of intervention. | Not assessed |
| Sims-Gould et al. (2017) | Risk of bias assessment was done using Cochrane Collaboration’s Risk of Bias criteria. The review authors did not comment on the risk of bias in individual studies when interpreting the results of the review. However, **45% of the studies had low risk of bias regarding sequence generation and allocation concealment, 60% of the studies had low risk of bias regarding blinding of participants and personnel and incomplete data, more than 90% of the studies had low risk of bias regarding selective reporting, and more than 70% were free of other bias**. | Not assessed |
| Dedeyne et al. (2017) | Risk of bias in the individual studies was assessed by the methodological index for nonrandomized studies (MINORS). Each of the 12 criteria was scored 0 (not reported), 1 (reported but inadequate), or 2 points (reported and adequate), resulting in a total quality score ranging from 0 (low quality) to 24 (high quality).  **The total methodological quality scores of the included studies ranged from 16 “moderate” to 23 “excellent”.** | Not assessed |
| Coll-Planas et al. (2017) | The Cochrane risk of bias tool was adapted to assess the internal validity rating random sequence generation, allocation concealment, blinding and incomplete outcome data. Blinding was excluded from the summary risk of bias due to its difficulty to be implemented in social capital interventions**. Over the 8 intervention studies targeting loneliness, 1 had a low risk of bias, 1 had unclear risk of bias and 6 had high risk of bias. The 2 studies with statistically significant effects favoring the social capital intervention had a high risk of bias**. | Not assessed |
| Snowden et al. (2015) | Over the 3 studies targeting loneliness, **the quality was rated as “limited” for 2 studies (social support intervention) and “at least fair” for 1 study (physical activity intervention)**. | The effectiveness was rated as insufficient because of the lack of studies. |
| Cohen- Mansfield & Perach et al. (2015) | Not clear | Not assessed |
| Theou et al. (2011) | The methodological quality of the included studies was evaluated by two reviewers using the Jadad methodological quality criteria scale. The double blinding criterion for this scale was modified due to the inability to blind allocation of study participants to an exercise intervention. A study could receive a Jadad score of zero to five.  The total Jadad methodological quality score of the studies ranged from 1 to 5**. Twenty-one studies had perfect scores, four scored 4, 13 scored 3, eight scored 2, and one scored 1.** No studies were excluded on the basis of their quality score since one of the criteria of the scale was modified as described in the methods section. | Not assessed |
| Walters et al. (2017) | Quality of the observational studies was assessed by one reviewer using the Newcastle–Ottawa Scale, which assesses the rigour of participant selection (up to a maximum of four stars), comparability (out of a maximum of two stars) and outcome assessments (out of a maximum of three stars). **Study quality was good across all studies.** | Not assessed |
| Fu et al. (2022) | Adequate random allocation sequences were used in Mountain et al., 2014. The randomization methods of the other studies were unclear because the authors only mentioned that allocation was randomized in their studies. No study mentioned allocation concealment. No study blinded the participants or the outcome assessments. One study did not use correct blinding methods (Mountain et al., 2014). | Not assessed |
| Heins et al. (2021) | Three quantitative studies were rated as strong (Selgers 2008, Czaja 2018, Yu 2019) [55,63,78]; three were rated as moderate (Matz-Costa 2018, Myhre 2017, Vanoh 2019) [71,73,80]; and six were rated as weak (Bickmore 2005, Woodward 2011, Kahlbaugh 2011, Goumopoulos 2017, Jansen-Kosternik 2020, Neil-Saztramko 2020) [46,52,56,65,67,74]. From the data in Table 2, it is apparent that the quality of most study designs was strong. Eight of the quantitative studies were at first considered as Randomized Controlled Trials (RCTs) (Bickmore 2005, Kahlbaugh 2011, Slegers 2008, Woodward 2011, Czaja 2018, Matz-Costa 2018, Yu 2019, Vanoh 2019) [46,52,55,56,63,71,78,80]. However, three studies did not describe the randomization procedure in their study methodology (Bickmore 2005, Kahlbaugh 2011, Woodward 2011)[46,52,56]. According to the EPHPP guidelines, these studies were classified as Clinical Controlled Trials (CCTs). One additional study was classified as a CCT because the randomized allocation of study participants conflicted with participants’ availability. The remaining quantitative studies were classified as single-group cohort studies (Goumopoulos 2017, Jansen-Kosternik 2020, Neil-Saztramko 2020) [65,67,74]. | Not assessed |
| Li et al. (2022) | We appraised the eligible studies according to the critical appraisal tool: The Joanna Briggs Institute Critical Appraisal Checklist for Randomized Controlled Trials. There were 13 items in the checklist rated by yes (score as 1), no (score as 1), and not applicable due to the study design/not reported by the original study (score as 0). Scores ranged from 6 to 10 out of 13. 70% score was classified as low risk, 60%-70% as moderate risk, and below 60% as high risk. 1/18 studies was low risk, 2 were moderate risk and 15 were classified as high risk. | Not assessed |
| Smith et al. (2019) | Not assessed due to absence of studies | Not assessed due to absence of studies |
| Tricco et al. (2022) | **All studies had low risk of bias for baseline outcome measurements** (100% low, 0% unclear, 0% high) and other bias (mainly funding bias; 100% low, 0% unclear, 0% high). **Two of the studies had low risk and two had unclear risk of bias for selective reporting** (50% low, 50% unclear, 0% high). **One study had high risk, two studies had unclear risk and one had low risk of bias for incomplete outcome data** (25% low, 50% unclear, 25% high). However, **three of four studies had high risk of bias for blinding of outcome assessment** (25% low, 0% unclear, 75% high), blinding of participants and personnel (25% low, 0% unclear, 75% high), random sequence generation (25% low, 0% unclear, 75% high) and allocation concealment (25% low, 0% unclear, 75% high). | Not assessed |
| **Other reviews** | | |
| Anton et al. (2017) | Not assessed | Not assessed |
| Hagan et al. (2014) | Not assessed | Not assessed |
| Kelaiditi et al. (2014) | Not assessed | Not assessed |
| Pool et al.  (2017) | The quality of the studies was assessed by using the Cochrane Collaboration‘s tool for assessing risk of bias  for randomized trials and the Newcastle-Ottawa scale for the non-controlled intervention studies. **Risk of bias was moderate in most studies, and high in one study**. | Not assessed |
| Puts et al.  (2017) | The quality of the included studies was assessed using the Mixed Methods Appraisal Tool (MMAT). **The quality was moderate to good for most studies.** | Not assessed |
| Wister et al. (2021) | Not assessed | Not assessed |
| Ibrahim et al. (2022) | Not assessed | Not assessed |
