# Supplementary material for: Effectiveness of interventions to address different types of vulnerabilities in community‐dwelling older adults: An umbrella review
Source: Campbell Syst Rev. 2023 May 9;19(2):e1323. doi: 10.1002/cl2.1323 (PMC10168691; doi:10.1002/cl2.1323)
Supplement: Supplementary file 7 — Supporting information. [file CL2-19-e1323-s008.docx]

rAdverse outcomes

| **Interventions** |  | **Adverse health outcomes** | **Magnitude of the effect** |
| --- | --- | --- | --- |
| **Systematic reviews** | | | |
| **Physical activity interventions** | Burton et al. (2019) | **Falls** → 6 trials (n=283)  -1 quasi-experimental single group study pre and post-test: **Reduction of falls and major Injuries**  -1 quasi-experimental pre- and post-test: **center-based participants had significantly less falls compared to home-based participants**  - 2 RCTs: no significant between groups differences  - 2 single group studies pre- and post-test: no significant between groups differences  **Pain** → 3 trials (n=146)  - 1 RCT: **Effect was in favor of the intervention group. Effect sizes were moderate**  -1 quasi-experimental pre- and post-test: **center-based participants had significantly improved pain compared to home-based participants**  - 1 single group study pre- and post-test: no significant between groups differences | Not reported  Not reported  -  -  Not reported  Not reported  - |
| **Reminiscence therapy** | Franck et al. (2016) | **Depression** → 1 RCT (n=130): **significant difference between groups on post-test and follow-up, with experimental group showing improvement in depressive symptoms** | Posttest: z= -7.09, p<0.0001;  Follow-up: z= -7.82, p<0.0001 |
| **Gender-based social clubs** | Franck et al. (2016) | **Depression** → 1 quasi-experimental pre- and post-test without control group (n=30): **depression decreased significantly over time for males (p=0.03)**, no significant difference for females (p=0.62)  **Anxiety** → 1 quasi-experimental pre- and post-test without control group (n=30): **anxiety decreased significantly over time for males (p=0.09)**, no significant difference for females (p=0.39) | Not reported  Not reported |
| **Radio program** | Franck et al. (2016) | **Depression** → 1 quasi-experimental pre- and post-test without control group (n=154): **depression decreased significantly (t(112)=2.99, p=0.003)** | Not reported |
| **Preventive, integrated care interventions** | Looman et al. (2019) | **Pain** → 4 trials (n=1926)  - 3 RCTs: no significant between groups differences  - 1 controlled before-and-after study: no significant between groups differences  **Falls** → 5 trials (n= 1884):  - 4 RCTs: no significant between groups differences  - 1 controlled before-and-after study: no significant between groups differences  **Falls** → 1 case-control study (n= 390): **significant outcome in favor of the experimental group**  **Mortality** → 15 trials (n=8388)  - 3 RCTs: **significant outcome in favor of the intervention**  - 8 RCTs: no significant between groups differences  - 1 cluster RCT: no significant between groups differences  - 3 controlled before-and-after studies: no significant between groups differences | -  -  -  -  Not reported  Not reported  -  -  - |
| **Dietary and physical activity and cognitive interventions** | (Dedeyne 2017) | **Falls** → 1 RCT (n=246): no significant between groups differences | - |
| **Dietary and physical activity interventions and nutritional advise** | (Dedeyne 2017) | **Falls** → 1 RCT (n=100): no significant between groups differences | - |
| **Social capital interventions** | Coll-Planas et al. (2017) | **Depression and anxiety** → 17 trials (n=2895)  - 5 RCTs: **statistically significant effects (p<0.05) favoring the social capital intervention**  - 12 RCTs: no significant between groups differences  **Mortality** → 6 trials (n=1433)  - 1 RCT: **statistically significant effects (p<0.05) favoring the social capital intervention**  - 5 RCTs: no significant between groups differences | Not reported  -  Not reported  - |
| **Shared activities (group interventions)** | Ibrahim et al. (2022) | Depression → 4 trials (n=602)  - 1 RCT (n=390): No difference in depressive symptoms.  - 1 RCT (n=46): Significant decrease in the experimental group.  - 2 NRS (n=166): Significant decrease in depressive symptoms. | - |
| **Other reviews** | | | |
| **Physical activity interventions** | Arantes et al. (2009) | **Depression** → 1 clinical trial/controlled trial/RCT (n=115): no significant between groups differences  **Falls** → 2 trials (n=254)  - 1 clinical trial/ controlled trial/RCT: Significant improvement in the risk of falling in the intervention and control groups, but the intervention group improved more  - 1 clinical trial/controlled trial/RCT: no significant between groups differences  **Institutionalization** → 1 clinical trial/controlled trial/RCT (n=188): Lower number of institutionalizations in IG (not significant)  **Pain** → 1 clinical trial/controlled trial/RCT (n=104): **Increase in pain after 18 months (p=0.05) in the control group** | -  Not reported  -  -  Not reported |

**Abbreviations**: RCT: randomized controlled trial, OR: odds ratio, CI: confidence interval

**Note:** Significant between-group differences are shown in bold

Health services use

| **Interventions** |  | **Health services use** | **Magnitude of the effect** |
| --- | --- | --- | --- |
| **Systematic reviews** | | | |
| **Physical activity interventions** | Burton et al. (2019) | 1 single group study pre- and post-test (n=50): **reduction in health care service access of 47% across the intervention** | Not reported |
|  | Theou et al. (2011) | **Nursing home admission** → 1 RCT (n=188): no significant between groups differences | - |
|  | Liam et al. (2020) | Long-term care admission → 1 NRS (n=1620): Over a four-year follow-up period, 15.2% of the intervention group participants were issued LTCI certifications as compared to 20.6% in the control group | Hazard Ratio: 0.73  (95% CI 0.62–0.86). |
| **Preventive, integrated care interventions** | Looman et al. (2019) | **Hospital admission** → 18 trials (n=15088)  - 1 RCT: **significant outcome in favor of the intervention**  - 1 controlled before-and-after study: **significant outcome in favor of the intervention**  - 9 RCTs: no significant between groups differences  - 2 cluster RCT: no significant between groups differences  - 1 stepped-wedge cluster RCT: no significant between groups differences  - 3 controlled before-and-after studies: no significant between groups differences  - 1 controlled before-and-after study: significant outcome both in favour of the intervention and the control group within one category (i.e. both decrease and increase in healthcare utilisation within one category)  **Emergency department admission** → 13 trials (n=12048)  - 2 RCTs: **significant outcome in favor of the intervention**  - 1 controlled before-and-after study: **significant outcome in favor of the intervention**  - 1 controlled before-and-after study: significant outcome in favor of the control group  - 3 RCTs: no significant between groups differences  - 2 cluster RCT: no significant between groups differences  - 1 RCT: significant outcome in favor of the control group  - 2 controlled before-and-after studies: no significant between groups differences  - 1 controlled before-and-after study: significant outcome both in favour of the intervention and the control group within one category (i.e. both decrease and increase in healthcare utilization within one category)  **Nursing home admission** → 20 trials (n=15310)  - 2 RCTs: **significant outcome in favor of the intervention**  - 9 RCTs: no significant between groups differences  - 1 RCT: significant outcome in favor of the control group  - 1 cluster RCT: no significant between groups differences  - 1 cluster RCT: significant outcome in favor of the control group  - 1 cluster RCT: not tested for significance  - 1 stepped-wedge cluster RCT: not tested for significance  - 4 controlled before-and-after studies: no significant between groups differences | Not reported  Not reported  -  -  -  -  Not reported  Not reported  Not reported  Not reported  -  -  Not reported  -  Not reported  Not reported  -  Not reported  -  Not reported  -  -  - |
| **Shared activities (group interventions)** | Ibrahim et al. (2022) | Doctor visits → 1 NRS (n=128): Significant improvement | - |

**Abbreviations**: RCT: randomized controlled trial

**Note:** Significant between-group differences are shown in bold

Costs

| **Interventions** |  | **Cost** | **Magnitude of the effect** |
| --- | --- | --- | --- |
| **Systematic reviews** | | | |
| **Physical activity interventions** | Arantes et a. (2009) | 1 clinical trial/controlled trial/RCT (n=104):  **Intervention group: greater expenditures on home modifications and purchase of gait assistance devices (p<0.001)**. **Control group: greater expenditures on institutionalization (p<0.01) and medical visits (p<0.01)**. No significant differences in overall expenditures | Not reported |
|  | Burton et al. (2019) | 1 RCT (n=186): **A significantly higher number of older people in the intervention group were identified for reduced paid caregiver support** | Not reported |
| **Preventive, integrated care interventions** | Looman et al. (2019) | **Costs** → 17 trials (n=10778)  - 1 RCT: **significant outcome in favor of the intervention**  - 8 RCTs: no significant between groups differences  - 2 RCTs: significant outcome in favor of the control group  - 2 cluster RCT: no significant between groups differences  - 1 stepped-wedge cluster RCT: no significant between groups differences  - 3 controlled before-and-after studies: no significant between groups differences | Not reported  -  Not reported  -  -  - |
| **Shared activities (group interventions)** | Ibrahim et al. (2022) | Healthcare cost → 1 RCT (n=390): Significant improvement in healthcare costs. | - |

**Abbreviations**: RCT: randomized controlled trial

**Note:** Significant between-group differences are shown in bold
